# Implication of transcription factor FOXD2 dysfunction in syndromic congenital anomalies of the kidney and urinary tract (CAKUT)

**DOI:** 10.1016/j.kint.2023.11.032

**Published:** 2024-04

**Authors:** Korbinian M. Riedhammer, Thanh-Minh T. Nguyen, Can Koşukcu, Julia Calzada-Wack, Yong Li, Nurit Assia Batzir, Seha Saygılı, Vera Wimmers, Gwang-Jin Kim, Marialena Chrysanthou, Zeineb Bakey, Efrat Sofrin-Drucker, Markus Kraiger, Adrián Sanz-Moreno, Oana V. Amarie, Birgit Rathkolb, Tanja Klein-Rodewald, Lillian Garrett, Sabine M. Hölter, Claudia Seisenberger, Stefan Haug, Pascal Schlosser, Susan Marschall, Wolfgang Wurst, Helmut Fuchs, Valerie Gailus-Durner, Matthias Wuttke, Martin Hrabe de Angelis, Jasmina Ćomić, Özlem Akgün Doğan, Yasemin Özlük, Mehmet Taşdemir, Ayşe Ağbaş, Nur Canpolat, Naama Orenstein, Salim Çalışkan, Ruthild G. Weber, Carsten Bergmann, Cecile Jeanpierre, Sophie Saunier, Tze Y. Lim, Friedhelm Hildebrandt, Bader Alhaddad, Lina Basel-Salmon, Yael Borovitz, Kaman Wu, Dinu Antony, Julia Matschkal, Christian W. Schaaf, Lutz Renders, Christoph Schmaderer, Manuel Rogg, Christoph Schell, Thomas Meitinger, Uwe Heemann, Anna Köttgen, Sebastian J. Arnold, Fatih Ozaltin, Miriam Schmidts, Julia Hoefele

**Affiliations:** 1 Institute of Human Genetics, Klinikum rechts der Isar, Technical University of Munich, TUM School of Medicine and Health, Munich, Germany; 2 Department of Nephrology, Klinikum rechts der Isar, Technical University of Munich, TUM School of Medicine and Health, Munich, Germany; 3 Department of Human Genetics, Radboud Institute for Molecular Life Sciences, Radboud University Medical Center, Nijmegen, The Netherlands; 4 Department of Bioinformatics, Hacettepe University Institute of Health Sciences, Ankara, Türkiye; 5 Institute of Experimental Genetics, German Mouse Clinic, Helmholtz Zentrum München, German Research Center for Environmental Health, Neuherberg, Germany; 6 Institute of Genetic Epidemiology, Medical Center – University of Freiburg, Faculty of Medicine, University of Freiburg, Germany; 7 Pediatric Genetics Unit, Schneider Children’s Medical Center of Israel, Petah Tikva, Israel; 8 Department of Pediatric Nephrology, Istanbul University-Cerrahpasa, Cerrahpasa Faculty of Medicine, Istanbul, Türkiye; 9 Institute of Experimental and Clinical Pharmacology and Toxicology, Faculty of Medicine, University of Freiburg, Germany; 10 Center for Pediatrics and Adolescent Medicine, Medical Center – University of Freiburg, Faculty of Medicine, University of Freiburg, Germany; 11 Institute of Molecular Animal Breeding and Biotechnology, Gene Center, Ludwig-Maximilians-University Munich, Munich, Germany; 12 German Center for Diabetes Research (DZD), Neuherberg, Germany; 13 Institute of Developmental Genetics, Helmholtz Zentrum München, German Research Center for Environmental Health, Neuherberg, Germany; 14 Chair of Developmental Genetics, TUM School of Life Sciences (SoLS), Technical University of Munich, Freising, Germany; 15 Department of Epidemiology, Johns Hopkins University Bloomberg School of Public Health, Baltimore, Maryland, USA; 16 Deutsches Institut für Neurodegenerative Erkrankungen (DZNE) Site Munich, Munich, Germany; 17 Munich Cluster for Systems Neurology (SyNergy), Adolf-Butenandt-Institut, Ludwig-Maximilians-University Munich, Munich, Germany; 18 Chair of Experimental Genetics, TUM School of Life Sciences (SoLS), Technical University of Munich, Freising, Germany; 19 Department of Pediatrics, Division of Pediatric Genetics, Acibadem Mehmet Ali Aydinlar University, School of Medicine, Istanbul, Türkiye; 20 Department of Pathology, Istanbul University, Istanbul Faculty of Medicine, Istanbul, Türkiye; 21 Department of Pediatric Nephrology, Istinye University Faculty of Medicine, Istanbul, Türkiye; 22 Faculty of Medicine, Tel Aviv University, Tel Aviv, Israel; 23 Department of Human Genetics, Hannover Medical School, Hannover, Germany; 24 Medizinische Genetik Mainz, Limbach Genetics, Mainz, Germany; 25 Department of Medicine IV, Medical Center – University of Freiburg, Faculty of Medicine, University of Freiburg, Germany; 26 Laboratoire des Maladies Rénales Héréditaires, Institut Imagine, Université Paris Cité, INSERM UMR 1163, Paris, France; 27 Department of Medicine, Division of Nephrology, Columbia University, New York, New York, USA; 28 Division of Nephrology, Boston Children’s Hospital, Harvard Medical School, Boston, Massachusetts, USA; 29 Raphael Recanati Genetics Institute, Rabin Medical Center, Petah Tikva, Israel; 30 Felsenstein Medical Research Center, Petah Tikva, Israel; 31 Institute of Nephrology, Schneider Children’s Medical Center of Israel, Petah Tikva, Israel; 32 Institute of Surgical Pathology, Medical Center – University of Freiburg, Faculty of Medicine, University of Freiburg, Germany; 33 CIBSS – Center for Integrative Biological Signaling Studies, University of Freiburg, Freiburg, Germany; 34 Department of Pediatric Nephrology, Hacettepe University Faculty of Medicine, Sihhiye, Ankara, Türkiye; 35 Nephrogenetics Laboratory, Hacettepe University Faculty of Medicine, Sihhiye, Ankara, Türkiye; 36 Center for Genomics and Rare Diseases, Hacettepe University, Sihhiye, Ankara, Türkiye

**Keywords:** CAKUT, chronic kidney disease, *FOXD2*, *PAX2*, renal hypoplasia, *WNT4*, urinary albumin-to-creatinine ratio (UACR)

## Abstract

Congenital anomalies of the kidney and urinary tract (CAKUT) are the predominant cause for chronic kidney disease below age 30 years. Many monogenic forms have been discovered due to comprehensive genetic testing like exome sequencing. However, disease-causing variants in known disease-associated genes only explain a proportion of cases. Here, we aim to unravel underlying molecular mechanisms of syndromic CAKUT in three unrelated multiplex families with presumed autosomal recessive inheritance. Exome sequencing in the index individuals revealed three different rare homozygous variants in *FOXD2*, encoding a transcription factor not previously implicated in CAKUT in humans: a frameshift in the Arabic and a missense variant each in the Turkish and the Israeli family with segregation patterns consistent with autosomal recessive inheritance. CRISPR/Cas9-derived *Foxd2* knockout mice presented with a bilateral dilated kidney pelvis accompanied by atrophy of the kidney papilla and mandibular, ophthalmologic, and behavioral anomalies, recapitulating the human phenotype. In a complementary approach to study pathomechanisms of FOXD2-dysfunction–mediated developmental kidney defects, we generated CRISPR/Cas9-mediated knockout of *Foxd2* in ureteric bud–induced mouse metanephric mesenchyme cells. Transcriptomic analyses revealed enrichment of numerous differentially expressed genes important for kidney/urogenital development, including *Pax2* and *Wnt4* as well as gene expression changes indicating a shift toward a stromal cell identity. Histology of *Foxd*2 knockout mouse kidneys confirmed increased fibrosis. Further, genome-wide association studies suggest that *FOXD2* could play a role for maintenance of podocyte integrity during adulthood. Thus, our studies help in genetic diagnostics of monogenic CAKUT and in understanding of monogenic and multifactorial kidney diseases.

## Graphical abstract

**Figure:**
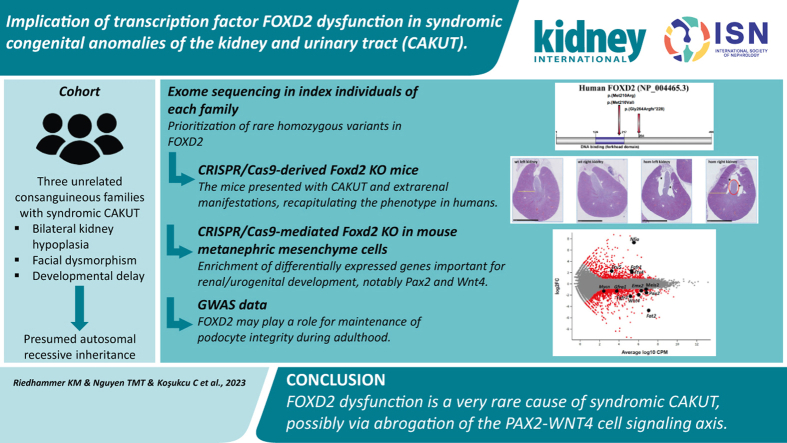

**Lay Summary**

Congenital anomalies of the kidney and urinary tract are the predominant cause of impaired kidney function in infants, children, and adolescents. The symptoms are diverse, ranging from relatively mild manifestations such as vesicoureteral reflux (backward flow of urine to the kidneys) to severe forms such as renal agenesis (absent kidneys). So far, in only ∼10% of the affected individuals, disease-causing variants in known disease-associated genes can be identified. Within this study, in 3 families with children affected by congenital anomalies of the kidney and urinary tract, rare variants in the gene *FOXD2* (forkhead box D2) could be identified. Further analyses, for example, of mice and renal cells, suggested that *FOXD2* could play a role in the renal and urogenital development and seems to be important for maintenance of the filtering function of the kidneys. The focus of this study was therefore on the characterization of *FOXD2* as a gene associated with the development of congenital anomalies of the kidney and urinary tract and its underlying pathomechanisms.

Congenital anomalies of the kidney and urinary tract (CAKUT) are the most important cause of renal replacement therapy in children aged 0 to 14 years in Europe (41.3%) and the most frequent cause of chronic kidney disease (CKD) up to the age of 30 years.^1^^,^^2^ CAKUT comprises a broad spectrum of malformations of the kidney and urinary tract, ranging from vesicoureteral reflux to renal agenesis leading to kidney failure requiring dialysis and kidney transplantation.^3^ CAKUT can occur in either isolated or syndromic forms.^4^^,^^5^

Much progress has been made over the last years concerning disease-associated gene identification by using next-generation sequencing approaches. Several monogenic forms of CAKUT have been identified, mostly inherited in an autosomal dominant, but also autosomal recessive, fashion. More than 45 genes associated with isolated monogenic CAKUT and >135 genes associated with syndromic CAKUT are known.^6^^,^^7^ Furthermore, copy number variants play an important role in CAKUT.^4^ Nevertheless, only ∼10% of CAKUT cases can be genetically solved, and incomplete penetrance and variable expressivity are often observed. Monogenic CAKUT is more frequent if severe kidney affection occurs (renal agenesis/dysplasia) in syndromic/familial cases and if there is parental consanguinity.^3^^,^^8^ Judging from familial clustering of CAKUT and a large number of CAKUT manifestations in monogenic mouse models, there is evidence for not yet described monogenic causes of CAKUT in humans.^3^^,^^9^

Among the genes implicated in CAKUT by mouse models are 2 encoding transcription factors of the forkhead box (*FOX*) gene family: *Foxd1* and *Foxd2*. Both genes are highly similar in structure and sequence and likely have partially redundant functions.^10^ No human individuals with CAKUT resulting from *FOXD1* or *FOXD2* disease-causing variants have been described to date. Here, we report the identification of a homozygous frameshift variant and 2 homozygous missense variants in *FOXD2* in 3 unrelated families implicated in autosomal recessive syndromic CAKUT with renal hypoplasia, facial dysmorphies, and proteinuria. Systematic phenotyping of *Foxd2* knockout (KO) mice did not only confirm the renal anomalies previously reported in the literature but also recapitulated extrarenal features observed in affected individuals.^10^ Transcriptome analyses in a *Foxd2* mutant mouse metanephric mesenchyme cell line suggests *Pax2* and *Wnt4* as *Foxd2* downstream targets, providing a putative pathomechanism potentially involving diversion of the lineage identity toward renal stroma cells.

## Methods

Detailed methods can be found in the Supplementary Methods.

### Human genetics

**Figure 1:**
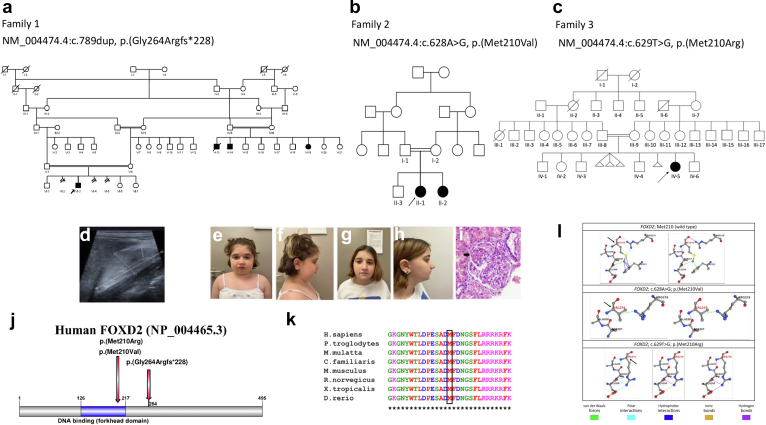
**Pedigree of (a) the Arabic family, (b) the Turkish family, and (c) the Israeli family.** The solid symbols indicate affected individuals; circles, females; squares, males; arrows, index and also affected cousin. The double horizontal bars indicate consanguinity. (**d**) Renal ultrasound of the Arabic index individual (right kidney). (**e–h**) Facial photographs of the siblings of the Turkish family (downslanting palpebral fissures, deeply set eyes, laterally extended eyebrows, micro-retrognathia, and mild ptosis on the left eye). (**i**) Renal biopsy of individual II-2 of the Turkish family. Note glomerular hypertrophy, mesangial increase, and segmental sclerosis (arrow; hematoxylin and eosin stain). (**j**) Illustration of the FOXD2 (forkhead box D2) protein showing location of the variants. (**k**) Multiple alignment analysis using Clustal X software (http://www.clustal.org/clustal2) demonstrating that methionine (M) in position 210 of *FOXD2* is evolutionary conserved. (**l**) Three-dimensional structural analyses showing the noncovalent interactions between the mutated site and its surrounding residues for the p.(Met210Val) and p.(Met210Arg) variants in *FOXD2*. (i) Wild-type structure of residue MET210 having 4 polar interactions (sky blue), 2 van der Waals interactions (green), 4 hydrophobic interactions (blue), and 2 hydrogen bonds (purple). (ii) Mutant-type residue 210VAL (family 2) having 2 polar interactions (sky), 1 van der Waals interaction (green), and 1 hydrogen bond (purple). (iii) Mutant-type residue 210ARG (family 3) having 5 polar interactions (sky blue), 3 hydrogen bonds (purple), and 1 ionic bond (gold). Duplicate/triplicate structures are illustrated to make overlapping interactions or bonds visible. To optimize viewing of this image, please see the online version of this article at www.kidney-international.org.

In case of family 1 (Figure 1a), the study was approved by the local ethics committee of the Technical University of Munich (521/16 S) and performed according to the standard of the Helsinki Declaration of 2013. Written informed consent was obtained from all participants or their legal guardians. The family described in this study is part of a larger hereditary kidney disease cohort (“NephroGen”; >1000 families), including 313 CAKUT families, located at the Institute of Human Genetics, Technical University of Munich, Munich, Germany.

A DNA sample of the index case (VI-3) was analyzed by exome sequencing (ES) using the SureSelect Human All Exon 60 Mb V6 kit (Agilent) and the HiSeq 4000 (Illumina) sequencer as previously described.^11^ Other family members were analyzed by targeted Sanger sequencing using the ABI capillary sequencer 3730 (Applied Biosystems). To identify additional individuals affected by CAKUT and carrying biallelic damaging *FOXD2* variants, a number of collaborating laboratories (total of 5000 cases analyzed) were contacted and a *GeneMatcher* search was conducted.^12^

Family 2 (Figure 1b) was identified through *GeneMatcher*,^12^ and all individuals participating in this study were enrolled after obtaining signed informed consent in accordance with human subject research protocols approved by the Ethics Committee of Istanbul University – Cerrahpasa, Cerrahpasa Medical Faculty (No.: 139896, Date: October 22, 2020).

Microarray studies were performed in affected individuals (II-1 and II-2) using the Illumina Infinium HumanCytoSNP-12 v2.1 (300K) microarray chip and Illumina BlueFuse Multi v4.5 Software. ES was performed in the 2 affected girls, 1 unaffected boy, and their parents (i.e., II-1, II-2, II-3, I-1, and I-2; Figure 1b) using the Human Core Exome Panel v3.0 (Twist Bioscience) and the MGI-T7 (MGI Tech) sequencer. Homozygosity mapping was performed using ES data via HomSI software (a homozygous stretch identifier from next-generation sequencing data), which was developed by the Advanced Genomics and Bioinformatics Research Center in the Scientific and Technological Research Council of Türkiye.^13^ Sanger sequencing was performed in all individuals of the family to validate the sequence variation prioritized by ES using the ABI 3130 genetic analyzer.

**Figure 2:**
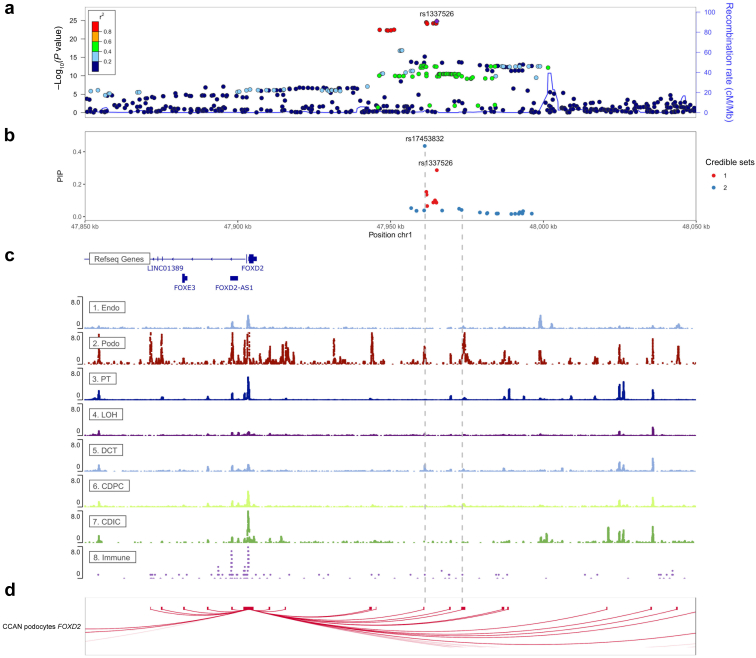
**From top to bottom: (a) regional association plot, (b) posterior inclusion probability (PIP) plot, (c) RefSeq gene track and 8 snATAC-seq peak tracks, and (d) podocyte cis-coaccessibility network (CCAN) at the *FOXD2* (forkhead boxD2) locus.** In the regional association plot, the association *P* values from a genome-wide association study of the urine albumin-to-creatinine ratio (UACR) from the UK Biobank were plotted on the −log_10_ scale. The correlation (*r*^2^) of genetic variants in the region with the lead single nucleotide polymorphism (SNP) rs1337526 is indicated as color gradients. The *r*^2^ was based on 1000 Genomes EUR genotype data phase 3, November 2014 release. In the PIP plot, only SNPs in 95% credible sets (i.e., the set of SNPs that contains a variant with effect on UACR with probability ≥ 95%) are shown. The color indicates the credible set membership. The vertical axis indicates the PIP value of the SNPs. The 8 ATAC-seq open chromatin tracks were retrieved from http://www.susztaklab.com/human_kidney/igv/. Two gray vertical dashed lines were placed at positions where credible set SNPs overlap with open chromatin peaks in a podocyte, a relevant cell type for albuminuria. The CCAN plot shows all connections with a coaccessibility score of >0.25 targeting the *FOXD2* promoter as red curved lines. CDIC, collecting duct intercalated cell; CDPC, collecting duct principal cell; chr1, chromosome 1; DCT, distal convoluted tubule; Endo, endothelial cell; Immune, immune cell; LOH, loop of Henle; Podo, podocyte; PT, proximal tubule.

**Figure 3:**
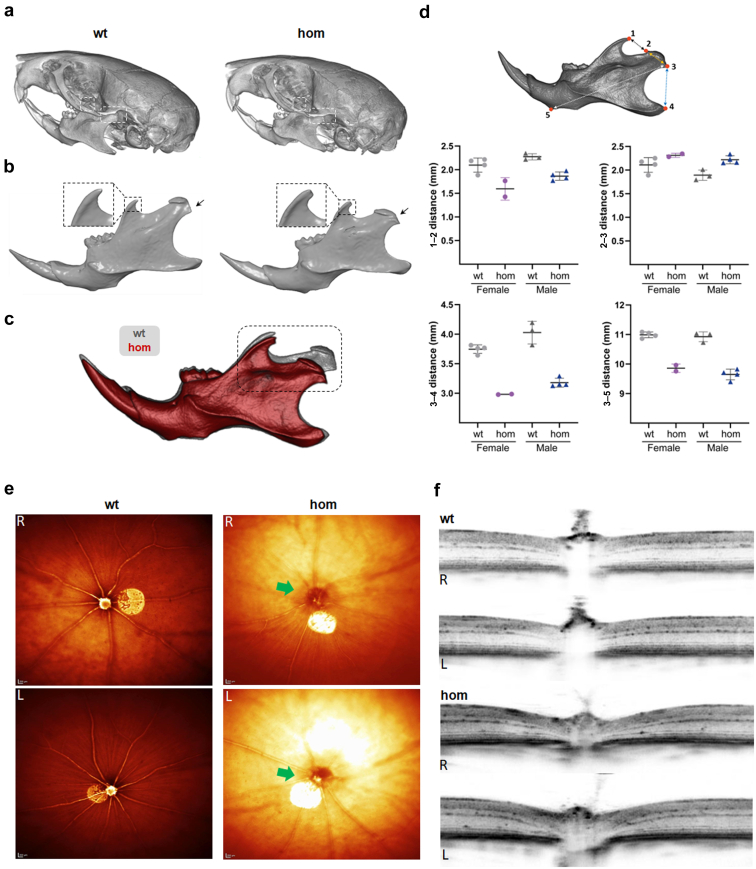
**Mandibular alterations and defective optic disc in homozygous (hom) *Foxd2* (forkhead boxD2) knockout (KO) mice.** Micro–computed tomography representative images of the (**a**) skull and (**b**) mandible from control (wild-type [wt]) and hom *Foxd2* KO male mice. In (**a**), the coronoid (left side) and condylar (right side) processes are highlighted by dash line rectangles; and in (**b**), by a magnified inset and arrows, respectively. (**c**) Volume matching of male control (gray) and mutant (red) mandibles at an identical scale, allowing easy qualitative comparison of the described morphological changes. (**d**) Mandibular morphometric analysis comparing distance measurements between several anatomical landmarks: (1) dorsal-most point of the coronoid process; (2) anterodorsal side of the condylar process; (3) ventral-most point of the condylar process; (4) posterior-most point of the angular process; and (5) ventral-most point of the front lower part of the mandible. The condylar process is shorter (distances 1–2 and 3–4) and slightly wider (distance 2–3) in mutants than in control animals. These landmarks have already been described in rodents.^44^*n* = 4 wt and *n* = 2 homozygous females and *n* = 4 wt and *n* = 4 homozygous males. Single values and mean ± SD values are shown. Test for statistical significance was not performed because of the low animal number. (**e**) Representative images of eyes of 16-week-old hom *Foxd2* KO mice and age-matched wt littermates using the *en face* optical coherence tomography modality. *Foxd2*^−/−^ fundus appearance around the optic nerve displays a darker signal, indicating alterations of the optic disc (green arrow). (**f**) Spectral-domain optical coherence tomography images through the optic nerve showed altered optic nerve morphology. Eleven of 17 hom *Foxd2* KO mice showed clear optic disc alterations. To optimize viewing of this image, please see the online version of this article at www.kidney-international.org.

**Figure 4:**
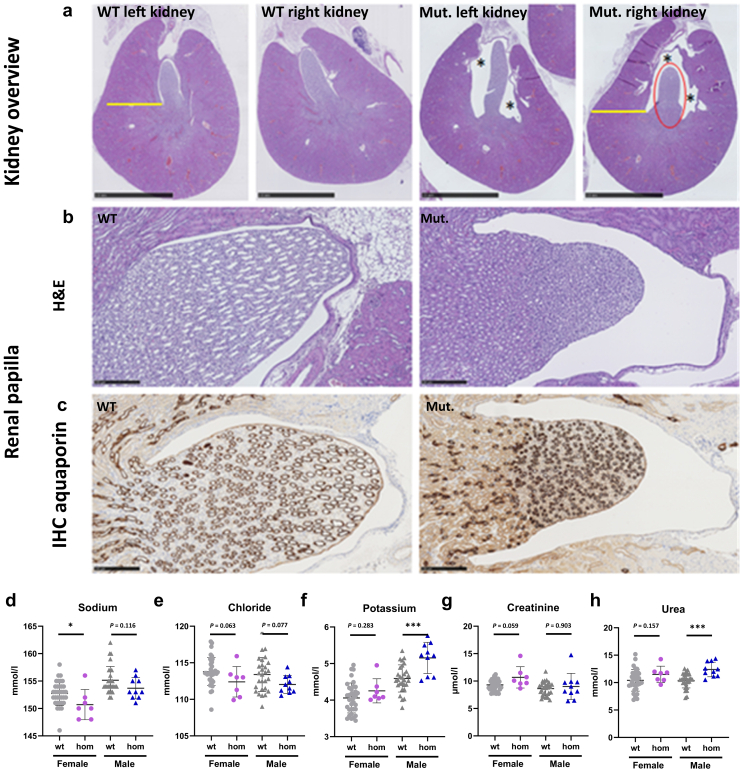
**Histopathological renal alterations in 16-week-old mice.** (**a**) Representative overview pictures of hematoxylin and eosin (H&E) staining from the left and right kidneys of wild-type (wt) and homozygous (hom) *Foxd2* (forkhead box D2) knockout (KO) mice. Note the normal kidney morphology observed in the wt animal compared with bilateral mild renal pelvis dilation (marked with an asterisk) in the hom KO mouse. Note the reduced size of the renal papilla (marked in red) and the cortex, which is clearly narrowed (yellow). (**b**) H&E stains show a higher magnification of the renal papilla. (**c**) Immunohistochemistry (IHC) aquaporin-2 clearly shows a reduction in diameter of the collecting ducts in the mutant mouse. (**d–h**) Plasma clinical chemistry analyses of parameters frequently altered in the case of renal dysfunction, including plasma levels of (**d–f**) electrolytes, (**g**) creatinine, (**h**) and urea. Significance according to the Mann-Whitney test: ∗*P* < 0.05, ∗∗∗*P* < 0.001. Mut., mutant; NS, not significant. To optimize viewing of this image, please see the online version of this article at www.kidney-international.org.

**Figure 5:**
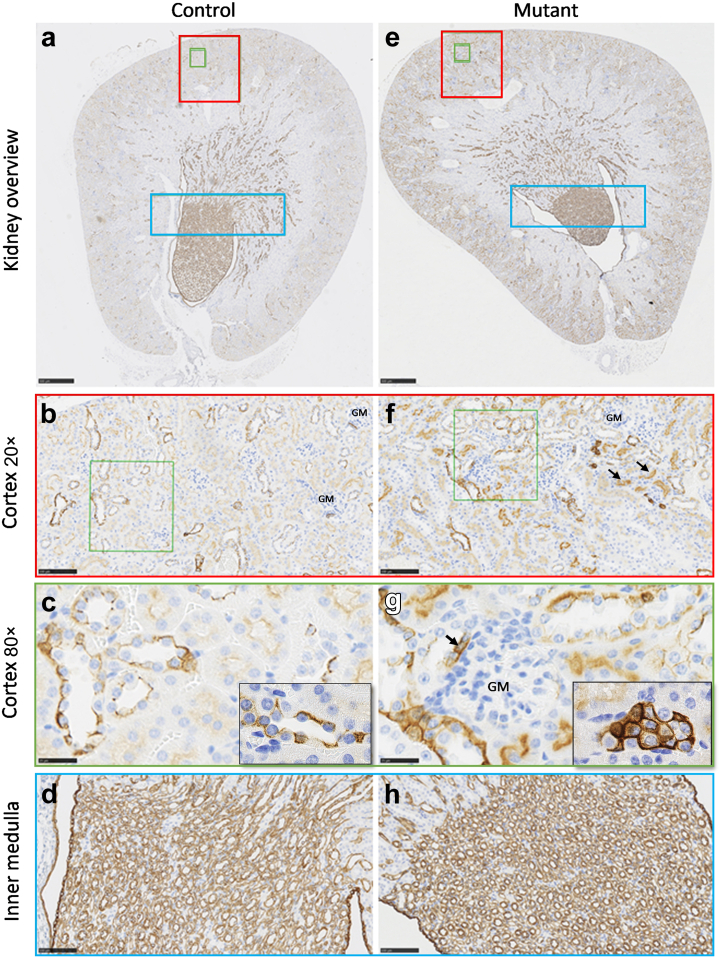
**Homozygous *Foxd2* (forkhead boxD2) knockout (KO) increases cortical cytokeratin 8 (CK8) expression.** Representative examples of CK8 staining tissue image analysis of the renal cortex. Kidney overview with the marked cortex and inner medulla of (**a**) a control (wild type) and (**e**) a mutant (homozygous KO) animal. The magnified renal cortex of (**b,c**) a control and (**f,g**) a mutant kidney. The magnified inner medulla of (**d**) a control and (**h**) a mutant kidney. Brown indicates CK8 H-DAB (hematoxylin and 3,3'-diaminobenzidine) immunohistochemistry (see also Supplementary Figure S5). GM, glomerulus. To optimize viewing of this image, please see the online version of this article at www.kidney-international.org.

In case of family 3 (Figure 1c), the study was approved by the Rabin Medical Center Institutional Review Board (6826). Signed informed consent for participation in the study was received from the family. Chromosomal microarray analysis was performed using the CytoScan 750K Array (Applied Biosystems). The index case underwent commercial multigene panel testing (Blueprint Genetics). Subsequently, duo ES for the proband (Figure 1, Figure 2, Figure 3, Figure 4, Figure 5) and her mother (Figure 1c, III-9) was performed at the Genetics Institute in the Tel Aviv Sourasky Medical Center. Targeted capture of protein-coding regions was performed using xGen Exome Hyb Panel v2 (Integrated DNA Technologies). Paired-end libraries were sequenced on the NovaSeq 6000 system (Illumina). At least 97% of target bases were covered at ≥20× (95% at >100×). Data analysis was performed at the Raphael Recanati Genetics Institute, Petah Tikva, Israel, using the Emedgene platform (Emedgene Technologies) and the Franklin genetic analysis and variant classification platform (Franklin by genoox; https://franklin.genoox.com). Because no disease-causing variant was identified, data were subsequently reanalyzed for suspicious, rare homozygous variants, and a variant in *FOXD2* was prioritized. While this manuscript was in preparation, a preprint version describing the findings in families 1 and 2 was published in *medRxiv*.^14^ Upon reanalysis, the striking clinical and molecular similarities between the affected individuals in these families and the index case in family 3 were noted.

The GeneBank (National Center for Biotechnology Information) sequence *FOXD2* NM_004474.4 (corresponding to the canonical Ensembl transcript ENST00000334793.6) was used as reference. NP_004465.3 protein sequence was also selected for further bioinformatic analyses.

#### *In silico* prediction and multiple sequence alignment for missense variants, and protein modeling for *FOXD2* p.(Met210Val) and p.(Met210Arg) missense variants (families 2 and 3)

*In silico* predictions were performed using Sorting Intolerant From Tolerant (SIFT; https://sift.bii.a-star.edu.sg/), Protein Variation Effect Analyzer (PROVEAN; http://provean.jcvi.org/index.php/), Polymorphism Phenotyping v2 (PolyPhen-2; http://genetics.bwh.harvard.edu/pph2/), Mutation Taster (https://www.mutationtaster.org/), Combined Annotation Dependent Depletion (CADD; https://cadd.gs.washington.edu/), Rare Exome Variant Ensemble Learner (REVEL; https://sites.google.com/site/revelgenomics/?pli=1),^15^ ClinPred (https://sites.google.com/site/clinpred/),^16^ and MutPred2 (http://mutpred.mutdb.org/).^17^

Multiple sequence protein alignment was performed using Clustal Omega version 1.2.2 (https://www.ebi.ac.uk/Tools/msa/clustalo/).

FOXD2 protein structure and Protein Data Bank (PDB) files were absent in the RCSB Protein Data Bank (https://www.rcsb.org/). Therefore, the preexisting model based in the AlphaFold database (https://alphafold.ebi.ac.uk/entry/O60548) was used. Gibbs free energy minimization of the wild-type (wt) protein structure was performed with the RepairPDB command of FoldX software^18^ with the following syntax:

foldx --command=RepairPDB --pdb= AF-O60548-F1-model_v2.pdb --water=CRYSTAL.

Four *in silico* tools, which estimate the impact of missense variants on protein stability and calculate the changes in unfolding Gibbs free energy, ΔΔ*G*, were used. Protein stability predictions were calculated with DynaMut2,^19^ INPS-3D,^20^ FoldX,^18^ and PremPS^21^ using the AlphaFold structure of FOXD2 as an input PDB file.

The ΔΔ*G* values between the wt protein and results of altered amino acid substitution were calculated for the p.Met210Val and p.Met210Arg variants in *FOXD2*. Finally, 3-dimensional (3D) structures of the wt and altered FOXD2 protein structures were generated with DynaMut2. The ΔΔ*G* values (in kilocalorie per mole) for both variants were interpreted as follows: Predicted ΔΔ*G* < 0 is destabilizing in the case of DynaMut2 and INPS-3D. Predicted ΔΔ*G* > 0 is destabilizing in the case of FoldX 5.0 and PremPS.

### Clustered regularly interspaced short palindromic repeats (CRISPR)/Cas9 gene editing in mice and mouse phenotyping

Mice were maintained in individually ventilated cages with water and standard mouse chow according to the Directive 2010/63/EU, German laws, and German Mouse Clinic (GMC) housing conditions. All tests were approved by the responsible authority of the district government of Upper Bavaria, Germany.

The *Foxd2* KO mouse model was derived using CRISPR/Cas9 technology using the web-based CRISPOR design tool^22^ and systematically characterized in the GMC phenotyping screen as previously described.^23^^,^^24^ Homozygous *Foxd2* KO mice (10 males, 7 females), heterozygous *Foxd2* KO mice (8 males, 10 females), and wt controls (33 males, 36 females) were analyzed by the GMC at Helmholtz Zentrum München, Neuherberg, Germany (http://www.mouseclinic.de).^23^, ^24^, ^25^ The phenotypic tests were part of the GMC screen and performed according to standardized protocols as previously described.^26^, ^27^, ^28^, ^29^ More details and the description of the immunohistochemistry and image analysis can be found in the Supplementary Methods.

### Cell culture, *Foxd2*-deficient metanephric mesenchyme cell model generation using CRSPR/Cas9 technology, and transcriptome analyses

CRISPR/Cas9 gene targeting on mk4 metanephric mouse cells was performed as previously described.^30^^,^^31^ Bioinformatics analysis was performed using the Galaxy platform (https://usegalaxy.org/) as previously described.^32^ Quantitative polymerase chain reaction (qPCR) in different mk4 clones carrying different homozygous frameshift alleles to exclude clonal effects was performed using the GoTaq qPCR Master Mix (Promega; catalog number A6001). Immunofluorescence analyses were performed as previously described (anti-Pax2 antibody: ab79389 [Abcam], 1:200).^33^ Gel electrophoresis and Western blotting were likewise performed as previously described (anti-Pax2 antibody: ab150391, EPR8586 [Abcam]).^34^

### 3D cell culture

CMUB-1 and mK cells were cultured in Dulbecco’s modified Eagle’s medium with GlutaMax medium with addition of 10% fetal bovine serum, 1% penicillin/streptomycin, and 1% sodium pyruvate at 37 °C under 5% CO_2_. To obtain the mK3 cell supernatant, mK3 cells were cultured 48 hours in serum-free medium before medium collection. Thirty milliliters of medium were then filtered using a 0.2 μm filter and added to a centrifugation concentration filter system with cutoff pore sizes of 3 kDa and centrifugation performed at 4000*g* for 30 minutes, resulting in an ∼50-times concentration. For CMUB-1 3D Matrigel cell cultures, Lab-Tek II Chamber Slides (Thermo Fisher) were coated with a Matrigel–cell suspension and 150 μl of medium was added after solidification.

### *FOXD2* genome-wide association study locus fine mapping

A previous genome-wide association study (GWAS) meta-analysis of the urinary albumin-to-creatinine ratio (UACR) has identified the *FOXD2* locus.^35^ We therefore attempted to identify the responsible variants underlying the association signal by using the statistical fine-mapping method SuSiE.^36^ SuSiE selects single nucleotide polymorphisms (SNPs) with a high probability to causally affect a given trait—here the UACR—even if a genetic locus contains many highly correlated genetic variants and/or multiple SNPs with a causal effect. We used the R package susieR (version 0.12.27) to fine-map the 1 MB genomic region centered at the SNP showing the strongest statistical association with UACR, rs1337526. For linkage disequilibrium matrix calculation, we used a genotype set of 15,000 randomly selected participants of European ancestry from the UK Biobank as in Teumer *et al.*^35^ To match the linkage disequilibrium reference as closely as possible, GWAS summary statistics of UACR based on data from 436,392 participants of the UK Biobank (application number 20272) were used as input. We used the default parameters in susieR functions, except for setting var_y to 1 and max_iter to 100000. The identified credible set SNPs were screened for their association with the UACR in up to 127,862 independent participants of the CKDGen Consortium.^35^ For visualization, credible set SNPs were positioned in the IGV browser of the human kidney snATAC-seq open chromatin peaks (http://www.susztaklab.com/human_kidney/igv/) from Sheng *et al.*^37^ to examine their position with respect to cell type–specific open chromatin peaks. The regional association plot was created by LocusZoom version 1.4.^38^ The podocyte cis-coaccessibility network was kindly provided by Muto *et al.*^39^ Only connections with a coaccessibility score of >0.25 targeting the *FOXD2* promoter were displayed.

We performed phenome-wide association studies of the 2 most likely causal fine-mapped variants using GWAS summary statistics of human traits and diseases from the UK Biobank^40^ (TOPMed-imputed PheWeb at https://pheweb.org/UKB-TOPMed/) and FinnGen^41^ (release DF9 data at https://r9.finngen.fi/). Phenome-wide association study queries were based on the rs identifier of the variants.

### Kidney gene expression profiles

To determine the localization of *FOXD2* gene expression within the adult human kidney, we leveraged publicly available single-cell RNA sequencing data from 47 samples from the Kidney Precision Medicine Project (accessible through https://atlas.kpmp.org/). Lake *et al.* provided a detailed description of the experimental setup and cell type identification processes.^42^

### Code availability

Codes for transcriptome analysis and plotting are available under https://github.com/gwangjinkim/foxd2_analysis.

### Ethics approval and consent to participate

This project has received approval from the local ethics committee of the Technical University of Munich (521/16 S), from the Ethics Committee of Istanbul University – Cerrahpasa, Cerrahpasa Medical Faculty (No: 139896, Date: October 10, 2020), as well as from the Rabin Medical Center Institutional Review Board in Petah Tikva (6826). Human samples collected as part of the Kidney Precision Medicine Project (KPMP) consortium (https://KPMP.org) were obtained with informed consent and approved under a protocol by the KPMP single institutional review board of the University of Washington Institutional Review Board (IRB 20190213). All participants provided informed consent, and the research conformed to the principles of the Helsinki Declaration.

## Results

### Clinical case

#### Family 1 (Arabian Peninsula)

In this consanguineous family, the index individual (VI-3) had facial dysmorphism (low-set ears, hypertelorism, downslanting palpebral fissures, and retrognathia), developmental delay, and congenital bilateral hypoplastic kidneys (Figure 1d) and underwent allogenic kidney transplantation at 11 years of age. At 7 years of age, he had CKD G4 (estimated glomerular filtration rate 26 ml/min per 1.73 m^2^ [modified Schwartz formula]^43^) and proteinuria of 2.7 g/24 h. His parents are first-degree cousins. Three first cousins once removed—individuals V-13, V-14, and V-19 (see Figure 1a for a detailed pedigree of the family)—were similarly affected. V-19 had bilateral hypoplastic kidneys, developmental delay, and retrognathia. At 6 years of age, she had CKD G3 (estimated glomerular filtration rate 11 ml/min per 1.73 m^2^) and proteinuria of 5.0 g/24 h. Allogenic kidney transplantation was done at 7 years of age. Relatives V-13 and V-14, brothers of V-19, were also affected by a similar phenotype (Table 1; Supplementary Case Report).

**Table 1:** Overview of the clinical phenotype of the affected individuals of the Arabic, the Turkish, and the Israeli family

	Family 1: Arabic family				Family 2: Turkish family		Family 3: Israeli family
	**VI-3**	**V-13**	**V-14**	**V-19**	**II-1**	**II-2**	
Genetic and proband data							
*FOXD2* variant, chromosomal position (hg19)	chr1:g.47904596dup	n.t.	n.t.	chr1:g.47904596dup	chr1:g.47904435A>G	chr1:g.47904435A>G	chr1:g.47904436T>G
*FOXD2* variant, cDNA position and protein position (NM_004474.4, NP_004465.3)	c.789dup, p.(Gly264Argfs∗228)	n.t.	n.t.	c.789dup, p.(Gly264Argfs∗228)	c.628A>G, p.(Met210Val)	c.628A>G, p.(Met210Val)	c.629T>G, p.(Met210Arg)
gnomAD MAF	0	0	0	0	0	0	0
Zygosity	Homozygous	**−**	**−**	Homozygous	Homozygous	Homozygous	Homozygous
Inheritance	From parents	**−**	**−**	Only mother tested (heterozygous carrier)	From parents	From parents	Only mother tested (heterozygous carrier)
Consanguinity	+	+	+	+	+	+	+
Sex	Male	Male	Male	Female	Female	female	Female
Age at the last examination, yr	8	Deceased at age 6 yr	n.d.	n.d.	12.7	16.9	7
Kidney							
Congenital anomalies of the kidney and urinary tract	+ (Bilateral kidney hypoplasia)	+ (Hypospadias)	+ (Horseshoe kidney, hypospadias)	+ (Bilateral kidney hypoplasia)	+ (Bilateral kidney hypoplasia)	+ (Kidney hypoplasia on the left side)	+ (Bilateral kidney hypoplasia)
KF	+ (10 yr)	n.d.	+	+ (6 yr)	+ (6 yr)	-	+ (5 yr)
Kidney transplantation	+ (11 yr; allogenic)	−	+ (6 yr, 25 yr; allogenic)	+ (7 yr; allogenic)	+ (6 yr)	-	+ (7 yr; allogenic)
Neurodevelopment							
Developmental delay	+	+	+	+	+	+	+
Dysmorphic features							
Facial dysmorphies	+ (Mandibular retrognathia, micrognathia, severe dental abnormalities, high-arched palate, hypertelorism, downslanting palpebral fissures, exophthalmos, flat nasal bridge, low-set dysplastic ears)	+ (Micrognathia)	+ (Micrognathia, crowded teeth)	+ (Micrognathia, discrete retrognathia)	+ (Downslanting palpebral fissures, deeply set eyes, laterally extended eyebrows, micro-retrognathia, mild ptosis on the left eye, high palate, dental crowding)	+ (Downslanting palpebral fissures, laterally extended eyebrows, micro-retrognathia, left deviation of the nasal axis)	+ (Downslanting palpebral fissures, ptosis, mild retrognathia)
Other							
				▪ Imperforate anus (surgically treated as a neonate) ▪ Corneal abrasions	▪ Fusiform fingers ▪ Sandal gap in both sides ▪ Central obesity ▪ Left esotropia and bilateral posterior subcapsular cataract	▪ Tapering of distal phalanges of fingers ▪ Sandal gap in both sides ▪ Short toes ▪ Central obesity	▪ Trivial pulmonic stenosis ▪ Esotropia, hypermetropia ▪ Preauricular pit (also in mother and healthy brother)

*FOXD2*, forkhead box 2; gnomAD, Genome Aggregation Database (v.2.1.1, https://gnomad.broadinstitute.org); KF, kidney failure; MAF, minor allele frequency; n.d., no data; n.t., not tested.

#### Family 2 (Türkiye)

In this consanguineous family, the female index individual (II-1) was diagnosed with kidney failure at the age of 6 years. Initial ultrasonography showed bilateral hypoplastic kidneys with increased echogenicity. She received a preemptive kidney transplant from her father at the age of 6 years. In addition to kidney failure, the individual had dysmorphic findings including downslanting palpebral fissures, deeply set eyes, laterally extended eyebrows, micro-retrognathia, and mild ptosis on the left eye (Figure 1e and f and Table 1), as well as high palate, dental crowding, fusiform fingers, sandal gap in both sides, and central obesity noted on physical examination. Eye examination performed at the age of 6 years showed left esotropia and bilateral posterior subcapsular cataract as well as grade I hypertensive retinopathy.

The sister of the index individual (II-2) was referred to the pediatric nephrology department because of persistent proteinuria at the age of 7 years. Her height was 117 ± −2.61 cm, and she also had dysmorphic findings (Figure 1g and h and Table 1). Serum creatinine level was normal for the age (i.e., 0.35 mg/dl, corresponding to an estimated glomerular filtration rate of 138 ml/min per 1.73 m^2^; creatinine-based “bedside Schwartz” equation [2009]).^43^ Renal ultrasound showed bilateral increased renal parenchymal echogenicity and left kidney hypoplasia. Kidney biopsy was compatible with focal segmental glomerulosclerosis (FSGS; Figure 1i). Dysmorphic features included downslanting palpebral fissures, laterally extended eyebrows, micro-retrognathia, left deviation of nasal axis, tapering of distal phalanges of fingers, sandal gap in both sides, short toes, and central obesity. A full ophthalmological examination was found to be normal. At 17 years of age, laboratory findings were compatible with CKD G3 with an estimated glomerular filtration rate of 38 ml/min per 1.73 m^2^ (modified Schwartz formula).^43^

#### Family 3 (Israel)

The proband was born to parents of Bedouin descent who are first cousins. During pregnancy, at 35 weeks of gestation, small kidneys were noted. On the third day of life, she had an elevated creatinine level of 1.7 mg/dl, which gradually decreased. Around age 2 years, she first presented with nephrotic syndrome, which did not respond to steroid treatment. Renal ultrasound showed bilateral dysplastic kidneys, and the left kidney was noted to be in the left central abdomen with small hypoechogenic findings suspicious for cysts and possibly dilated renal pelvis. Kidney biopsy was planned but not done owing to difficult access to the kidneys. She was treated with angiotensin-converting enzyme inhibitors and angiotensin receptor blockers with fluctuations in the degree of proteinuria, but levels always remained within the nephrotic range. Treatment with tacrolimus was also attempted but discontinued because of a rapid increase in the individual’s creatinine levels and hyperkalemia. Her renal functions worsened over time, and, at the age of 5 years and a half, she was started on peritoneal dialysis. At the age of 7 years, she underwent allogenic kidney transplantation. Medical history is also significant for mild developmental delay, which resolved, esotropia, and hypermetropia. She follows up with cardiology for left ventricular dysfunction attributed to hypertension, which improved with treatment. She also had short stature below the third percentile in the growth chart since age 3 years, likely because of chronic kidney failure.

On the last examination, she had microcephaly (2.8 SDs below the mean), downslanting palpebral fissures, ptosis, mild retrognathia, and a small, prominent chin. She had a unilateral preauricular pit also seen in her mother and an unaffected brother.

### Genetic results

#### Family 1 (Arabian Peninsula)

ES of individual VI-3 (index) led to the prioritization of a homozygous frameshift variant in *FOXD2* NM_004474.4:c.789dup, p.(Gly264Argfs∗228). The variant is not listed in gnomAD (Genome Aggregation Database, v.2.1.1, http://gnomad.broadinstitute.org/) and predicted to result in a protein of nearly the same length as wt FOXD2 but with a changed amino acid composition within the C-terminal half of the protein, leaving the DNA-binding domain intact (Figure 1j). As *FOXD2* is a single-exon gene (NM_004474.4), nonsense-mediated decay due to the frameshift variant cannot be expected (see Supplementary Methods for details on the filtering process of ES and Supplementary Table S1 for a list of homozygous variants below minor allele frequency 1.0% detected with ES in the index individual of family 1). Supporting the hypothesis that *FOXD2* represents an essential gene during mammalian development, no *FOXD2* homozygous loss-of-function variants are reported in gnomAD v.2.1.1.

Subsequent Sanger sequencing confirmed the segregation of the variant: parents of VI-3 are heterozygous carriers of the variant. The clinically similarly affected maternal first cousin once removed (V-19) was found to be a homozygous carrier, while the maternal great-aunt (IV-6) and mother of V-19 are heterozygous carriers of this variant (Supplementary Figure S1). Further segregation of the variant could not be performed because of limited accessibility/missing consent of relatives.

#### Family 2 (Türkiye)

Homozygosity mapping revealed runs of homozygosity located in chromosome 1, ∼11.6 Mb long (chr1:g.47,607,000-59,155,000, hg19). The identified homozygous stretch showed that both affected siblings were sharing the runs of homozygosity; however, parents and the unaffected sibling were heterozygous for the region of interest (Supplementary Figure S2A). ES was then performed for the index case (II-1), affected and unaffected siblings (II-2 and II-3), as well as healthy consanguineous parents (I-1 and I-2). ES analyses resulted in the prioritization of a homozygous missense variant NM_004474.4:c.628A>G, p.(Met210Val) in *FOXD2* (predicted as deleterious with CADD, REVEL, ClinPred, and MutPred2), which is located within the disease-segregating homozygous region and is not listed in gnomAD (see Supplementary Table S2 for a list of filtered candidate variants detected with ES in the Turkish index individual).

Sanger sequencing results confirmed that the p.Met210Val variant in *FOXD2* is segregating with the disease in the family (Supplementary Figure S2B). The homozygous missense variant is located at the methionine residue at amino acid position 210 within the DNA-binding domain of FOXD2 (NP_004465.3), which is completely conserved among different species until the level of *Danio rerio* (Figure 1j and k).

#### Family 3 (Israel)

A chromosomal microarray analysis (Raphael Recanati Genetics Institute) was inconspicuous, but showed regions of homozygosity ≥3 Mb in 6.4% of all autosomal regions tested. A renal malformation panel and nephrotic syndrome panel (Blueprint Genetics) followed by ES for the index individual and her mother were negative for disease-causing variants that fully explained the index individual’s phenotype. Variants of uncertain significance in *FN1* and *TBC1D8B* and a single variant in *PKHD1* were detected, all of which were inherited from the unaffected mother (see Supplementary Table S3).

Because of parental consanguinity, revision of the exome data focusing on rare homozygous variants was performed, which identified a homozygous missense variant NM_004474.4:c.629T>G, p.(Met210Arg) in *FOXD2*. This variant is absent from the gnomAD database. It affects the same conserved amino acid as seen in family 2. The CADD PHRED score for this variant is 28.6.

### Gibbs free energy calculation and the change in 3D protein structure in FOXD2 missense variants c.628A>G, p.(Met210Val) and c.629T>G, p.(Met210Arg) (families 2 and 3)

The starting free energy Δ*G* for the FOXD2 protein model was 817.623 kcal/mol, and it was lowered to 719.079 kcal/mol to a more stable state after the RepairPDB command of FoldX was applied. 3D protein models for wt FOXD2 and the potential changes in noncovalent bonds caused by the p.(Met210Val) and p.(Met210Arg) variants are displayed in Figure 1l. The p.(Met210Val) variant decreased protein stability and was predicted as “destabilizing” with all 4, and the p.(Met210Arg) variant decreased protein stability and was predicted as “destabilizing” with 3 of 4 different protein stability prediction tools upon point mutations. The ΔΔ*G* scores calculated for both variants are displayed in Supplementary Table S4.

#### Fine mapping of the *FOXD2* UACR GWAS locus prioritizes 2 underlying variants

The *FOXD2* locus (1p33) has been previously associated with UACR in large GWAS meta-analyses of adult study participants.^35^ In agreement, the affected individuals carrying rare biallelic *FOXD2* variants show significant proteinuria, with one of them even showing the histological finding of FSGS on kidney biopsy. We now performed fine mapping of UACR-associated genetic variants in the *FOXD2* locus in 436,392 participants of the UK Biobank to prioritize the variants most likely to cause the association signal (Figure 2; see Methods). Fine mapping yielded 2 independent sets of UACR-associated variants that contained 8 and 21 variants, all of which were also associated with UACR in a direction-consistent manner in an independent sample of up to 127,862 participants of the CKDGen Consortium (Supplementary Table S5).^35^ The variants with the highest posterior probability to cause the association signal with UACR in each set were both common SNPs upstream of *FOXD2*, rs17453832 and rs1337526 (Figure 2b). Interestingly, using publicly available scATAC-seq data from the human kidney,^37^ rs17453832 overlaps accessible chromatin regions exclusively in podocytes, the relevant cell type for albuminuria of glomerular origin, as observed in FSGS (Figure 2c). Moreover, a cis-coaccessibility network derived from podocytes in an independent scATAC-seq data set^39^ revealed a putative connection between rs17453832 and the *FOXD2* promoter/exon (Figure 2d; see Methods), further supporting rs17453832 as a regulatory variant of *FOXD2* in podocytes that leads to an association with UACR. Additional investigation of *FOXD2* in single-cell RNA-sequencing expression data (see Methods) showed >2.69-fold enriched expression in podocytes compared to all other cell types (*P* = 5.9 × 10^−120^). Fine-mapped variants in the *FOXD2* locus were not associated with other diseases in the UK Biobank or the FinnGen data sets after correction for multiple testing (see Methods); the association with the lowest *P* value was with “gestational edema and proteinuria without hypertension” (*P* = 3 × 10^−5^; FinnGen).

#### Foxd2 deficiency in mice leads to multidevelopmental phenotypes

To investigate the consequences of Foxd2 dysfunction in mice, *Foxd2* KO mice were generated using CRISPR/Cas9 technology and comprehensively phenotyped. Reverse transcription polymerase chain reaction and cDNA analysis confirmed the absence of *Foxd2* transcript in homozygous mutant animals (Supplementary Figure S3A). Homozygous *Foxd2* KO mice were viable and were born approximately according to Mendelian distribution. Compared with wt and heterozygous littermates, more homozygous newborns died shortly after birth (Supplementary Figure S3B).

At 16 weeks, micro–computed tomography analyses revealed a changed mandible morphology in homozygous *Foxd2* KO mice (Figure 3a–d; *n* = 4 wt and *n* = 2 homozygous females and *n* = 4 wt and *n* = 4 homozygous males; using landmarks that have already been described in rodents^44^), including an abnormally shortened condylar process and a flattened tip of the coronoid process in male homozygous *Foxd2* KO mice (Figure 3b–d). Importantly, homozygous *Foxd2* KO mice display smaller mandibles (micrognathia or mandibular hypoplasia), as measured with distance 3–5 (Figure 3d).

Ophthalmic evaluation identified changes in the appearance of the optic disc, imaged using the *en face* optical coherence tomography modality as a darker ring around the optic nerve head (Figure 3e). Spectral-domain optical coherence tomography images revealed that homozygous *Foxd2* KO mice have posterior deformation of the optic nerve head surface (Figure 3f). We noted different severity in the alterations of the optic nerve in these mice. The total retinal thickness was not significantly altered; only for severely affected eyes, a much thinner total retinal thickness was measured (data not shown).

Although macroscopy did not reveal malformations of the urinary system, microscopic analysis by standard hematoxylin and eosin staining detected bilateral dilation of the renal pelvis. Among 6 homozygous *Foxd2* KO mice examined (4 males, 2 females), bilateral dilation of the renal pelvis was present in 2 homozygous males (33% penetrance; Figure 4a). A clearly narrowed cortex and a decreased size of the renal papilla (Figure 4a and b) accompanied the dilation. For better visualization of the papilla, we used immunohistochemical staining with aquaporin-2 as a marker for the collecting tubules and detected a reduction in the diameter of the ducts (Figure 4c). To rule out that the bilateral dilation of the renal pelvis observed in homozygous mutants represented a background lesion, we performed hematoxylin and eosin staining of kidneys from 126 wt animals randomized by age and background strain. We detected bilateral renal pelvis dilation in only 4 of 126 wt animals (Fisher exact test, *P* = 0.0239). Taken together, our results support the diagnosis of a renal pelvis dilation in homozygous mutant mice with a penetrance of 33%. The bilateral dilation of the renal pelvis was apparently not secondary to an obstructive lower urinary tract lesion. Additional histological parameters of the kidneys are described in Supplementary Table S6A and B and Supplementary Figure S4.

Further, we found a significantly increased (on average doubled) cytokeratin 8 expression level in the renal cortex of homozygous *Foxd2* KO mice compared with wt controls, determined by algorithm-based cell counting (2-sample *t* test, *P* < 0.001; Figure 5; Supplementary Figure S5), indicating tubular epithelial injury.

Plasma clinical chemistry parameters affected by renal dysfunction showed mild to moderate deviations in homozygous *Foxd2* KO mice compared with wt controls analyzed in parallel. Although sodium levels, and in trend also chloride levels, were slightly decreased predominantly in female homozygous *Foxd2* KO mice, maybe as a sign of volume overload due to reduced urine output, a significant increase in plasma potassium and urea levels in male homozygous *Foxd2* KO mice was observed (Figure 4d–f and h). The index individual of family 1 also had increased plasma potassium and urea levels owing to reduced kidney function, but plasma sodium levels were normal. Interestingly, although in general no increase in plasma creatinine concentrations in homozygous *Foxd2* KO mice was observed, 2 homozygous *Foxd2* KO animals showed high values compared with controls (Figure 4g). One of them was studied by histological analyses, and the high creatinine and elevated urea levels were in line with the already described histopathological alterations in the kidneys, including increased cytokeratin 8 expression compatible with increased fibrosis (Figure 5; Supplementary Figure S5).

Behavioral alterations consequent to Foxd2 loss were also observed. In the open field test of spontaneous reactions to a novel environment, 8-week-old homozygous *Foxd2* KO mice were clearly hypoactive and hypoexploratory (Supplementary Figure S6). This was indexed by decreased total distance traveled (2-way analysis of variance genotype effect: F_1,81_ = 19.93; *P* < 0.0001) and total rearing activity (2-way analysis of variance genotype effect: F_1,81_ = 59.36; *P* < 0.0001) compared to wt controls. Heterozygous *Foxd2* KO mice were also hypoactive and hypoexploratory compared with wt mice, albeit with less severity than homozygous KO mice (Supplementary Table S7).

#### Generation of Foxd2-deficient metanephric mesenchyme cell models

To further investigate FOXD2 function for proper renal development and to understand how biallelic FOXD2 dysfunction may cause bilateral renal hypodysplasia/CAKUT in humans and mice, *Foxd2*-deficient immortalized mouse metanephric cell models (mk4 cells) were generated using CRISPR/Cas9 technology. mk4 cells represent ureteric bud–induced metanephric mesenchyme cells undergoing epithelial conversion.

The targeted *FOXD2* region was chosen to closely replicate the homozygous frameshift variant identified in family 1. Four suitable mk4 clones carrying different homozygous *Foxd2* variants resulting in a change in the reading frame were chosen (Table 2; Supplementary Figure S7 [only clone F7 shown]). Foxd2 is a transcription factor previously implicated in renal development in mice^10^; however, at the time of *Foxd2* KO mouse analysis, RNA sequencing analysis was unavailable and the precise role of mammalian renal development has remained elusive. Therefore, transcriptome analysis comparing the homozygous mutant mk4 with control mk4 cells transfected with nontargeting single guide RNA (sgRNA) was performed. Clone F7 carrying the frameshift variant NM_00859.3:c.801insT (p.Tyr268Leufs∗109) was chosen as the frameshift that most closely resembles the one predicted for the allele of the index individual. Five biological replicates per condition were used for transcriptome analysis.

**Table 2:** *Foxd2* variants created for *in vitro* experiments using CRISPR/Cas9 technology

Clone	cDNA NM_00859.3
mk4 clone E4	c.794insA p.(Tyr265fs∗)
mk4 clone F6	c.794A>G,792-793delTAinsGGG p.(Tyr265Trypfs∗88)
mk4 clone F7	c.801insT p.(Tyr268Leufs∗109)
mk4 clone H9	c.800-803delCTTA p.(Tyr268Alafs∗83)

CRISPR, clustered regularly interspaced short palindromic repeats; *Foxd2*, forkhead box D2.

#### RNA sequencing results

**Figure 6:**
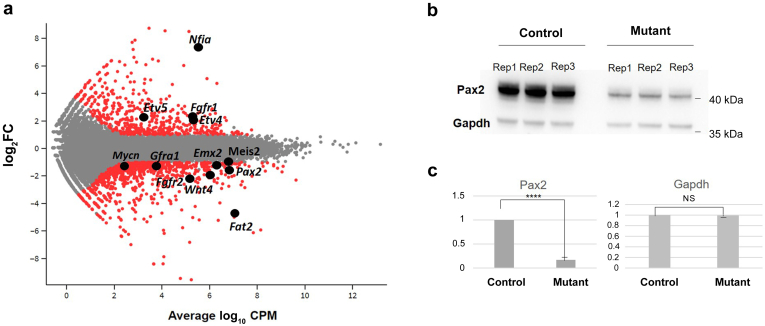
**Differential gene expression in *Foxd2* (forkhead boxD2) mutant cells.** (**a**) Glimma plot visualizing hits of interest. (**b**) Western blot analysis showing a strong reduction of Pax2 protein in mutant (clone E4) versus control cells using glyceraldehyde-3-phosphate dehydrogenase (Gapdh) as a loading control. *n* = 3 biological replicates per condition. (**c**) Quantitative analysis of Pax2 levels combining the 3 biological replicates per condition and Gapdh as a control protein, confirming a lower Pax2 protein amount in mutant cells than in controls. Student *t* test, ∗∗∗∗*P* < 0.0001. CPM, counts per million; FC, fold change; NS, not significant; Rep, replicate. To optimize viewing of this image, please see the online version of this article at www.kidney-international.org.

Transcriptomics analyses revealed a number of differentially regulated genes (Figure 6a). Gene Ontology (GO) term analysis of differentially expressed genes comparing clone F7 with a control clone revealed extracellular matrix organization (GO:0043062, *P* = 3.46 × 10^−8^)/extracellular matrix (GO:0030198, *P* = 3.378 × 10^−7^) as top hits followed by several terms related to renal/urogenital development as top hits among genes downregulated in the mutant clone: kidney development (GO:0001822, *P* = 5.73 × 10^−7^), renal system development (GO:0072001, *P* = 6.57 × 10^−7^), and urogenital development (GO:0001655, *P* = 7.45 × 10^−7^) with 24 to 27 of 6331 genes (adjusted *P* = 0.000655 for all 3 terms). Genes comprised were *Mmp17*, *Smad9*, *Fgf1*, *Emx2*, *Col4a4*, *Tfap2a*, *Sim1*, *Wnt2b*, *Adamts1*, *Tgfb2*, *Aqp1*, *Cys1*, *Pax2*, *Npnt*, *Egr1*, *Agt*, *Lrp4*, *Prlr*, *Col4a3*, *Igf1*, *Wnt4*, *Fgfr2*, *Id3*, *Fras1*, *Gli2*, *Pygo1*, and *Enpep* (Supplementary Figure S8A and B). Interestingly, among upregulated genes in *Foxd2* mutant versus control cells, top Gene Ontology term hits were related to leukocyte migration, antigen presentation, and chemotaxis followed by regulation of mitogen-activated protein kinase activity (GO:0043405, *P* = 2.96 × 10^−10^; Table 3; Supplementary Figure S8A and B). Transcriptome analysis of *Foxd2* mutant clone F7 compared with unedited control cells further suggested a significant upregulation of *Nfia* (log_2_ fold change = 7.27; adjusted *P* = 3.358 × 10^−7^; Figure 6a; Supplementary Figure S9A).

**Table 3:** Gene Ontology term analysis comparing mk4 clone F7 with wild type

	*P*	Adjusted *P*
Down		
Extracellular matrix organization	3.456442E−8	0.000152
Renal system development	6.571659E−7	0.000655
Urogenital system development	7.447146E−7	0.000655
Positive regulation of the steroid metabolic process	3.479562E−6	0.002186
Inner ear development	3.807702E−6	0.002186
Up		
Cell chemotaxis	6.340431E−11	4.250202E−8
Positive regulation of vasculature development	1.506551E−10	7.765204E−8
Antigen processing and presentation of endogenous antigen	1.544546E−10	7.765204E−8
Regulation of MAP kinase activity	2.957847E−10	1.321828E−7
Regulation of protein serine/threonine kinase activity	1.459391E−9	3.668544E−7

MAP, mitogen-activated protein.

qPCR validation of a set of selected targets showed a highly significant correlation with the relative expression levels determined by RNA sequencing (Pearson *R* = 0.8955; *P* < 0.0001; Supplementary Figure S9A). To exclude clone-specific effects, top gene hits from the Gene Ontology analysis with potential influence on renal development were confirmed by qPCR, comparing control cells with different *Foxd2* mutant mk4 clones (E4, F6, F7, and H9). These included single gene hits related to early kidney development and/or known to cause renal hypoplasia, such as *Pax2*, *Wnt4*, and *Fgfr2*. In addition, *Emx2* was found to be downregulated in *Foxd2* mutant mk4 cells compared with control cells. Although qPCR confirmed downregulation in all clones compared with control cells, the log 2 relative expression value for *Emx2* was <1, suggesting a rather minor effect (data not shown). In addition, higher gene expression of *Fat4* in mutant clones was confirmed compared with wt cells. qPCR also confirmed the strong downregulation of *Fat2* in all mutant clones compared with control cells (see Supplementary Figures S8C and S9B for a full set of qPCR-validated genes).

To investigate *Foxd2* KO on protein level, Western blotting was performed to confirm *Pax2* downregulation. This revealed markedly reduced Pax2 protein levels in *Foxd2*-deficient cells (3 independent experiments with 3 replicates each per condition; Figure 6b and c).

#### 3D tubuloid models

The human probands we identified presented with renal hypoplasia, which could be a result of impaired renal tubule development/branching morphogenesis defect during renal development to which reduced *Pax2* expression could contribute. Given the low penetrance of the CAKUT phenotype in *Foxd2* mutant mice also previously observed by Hogan and coworkers,^10^ in line with the “3R” principles for animal procedures, we decided to validate the effects of FOXD2 dysfunction on renal tubule formation using 3D tubuloid models to reduce the number of animals killed. To study the effect of Foxd2 dysfunction in metanephric mesenchyme cells on ureteric bud–derived tubule formation, we used so-called conditioned medium from wt and *Foxd2* mutant metanephric mesenchyme cells. This revealed significantly lower rates of tubule formation when conditioned medium from mutant cells was applied compared with conditioned medium derived from wt cells or medium treated with glial cell line–derived neurotrophic factor (GDNF) and fibroblast growth factor (FGF) additions (Supplementary Figure S10).

## Discussion

*FOXD2* encodes a transcription factor of the large, evolutionarily conserved, forkhead box gene family important for various processes in humans, such as organogenesis or metabolism.^45^ *FOX* genes share a “winged helix” DNA-binding domain consisting of 3 N-terminal a-helices, 3 b-strands, and 2 loops toward the C-terminal region. Different *FOX* genes have been associated with inherited human diseases and carcinogenesis.^46^ Notably, *FOXC1*, a gene associated with syndromic ophthalmological disease (Axenfeld-Rieger syndrome, type 3; Mendelian Inheritance in Men, MIM #602482), has recently been linked to autosomal dominant CAKUT.^47^

In addition, several other *FOX* genes (*FOXL2*, *FOXA2*, and *FOXA3*) have been proposed as candidate genes for monogenic CAKUT in a recent ES study.^48^

However, no representative of the “D” subfamily has been linked to a monogenic disease in humans thus far. *FOXD2* (*FREAC-9* and *MF-2*) is well expressed in the renal cortex.^49^ Of note, the *FOXD2* locus (1p33) has recently been associated with urinary albumin levels in genome-wide meta-analyses^35^ and *Foxd2* RNA is highly enriched in podocytes and was implicated in maintenance of podocyte integrity.^50^ Proteinuria up to the nephrotic range was reported in the index individual of family 1 and his female first cousin once removed, albuminuria with FSGS on kidney biopsy was present in individual II-2 of family 2, and nephrotic syndrome was reported in the affected individual of family 3 (see clinical cases in the Results section and Supplementary Case Report). In line with this, fine mapping of the *FOXD2* UACR GWAS locus revealed a likely podocyte-specific regulatory SNP (Figure 2). Together, this could support a role of *FOXD2* in podocyte maintenance and hence in proteinuric kidney disease in general.

Our study suggests that the abrogation of FOXD2 function can result in CAKUT. *Foxd2* KO mice have previously been reported to show renal hypoplasia and hydroureters, however, at a reduced penetrance of 40%.^10^ Also, in KO mice generated for this study, renal anomalies could only be identified at a reduced penetrance of 33% and were rather subtle (Figure 4a–c), underlining the variable expressivity of CAKUT. *Foxd2* shares close sequence homology with *Foxd1* (*Bf2*). A (partial) redundancy of the 2 could explain the reduced penetrance of CAKUT in *Foxd2* KO mice.^10^^,^^51^ Of note, in contrast to *Foxd2* mutant mice, homozygous *Foxd1* KO mice die shortly after birth of kidney failure due to hypoplastic kidneys.^52^ Furthermore, despite their phylogenetic relation and genomic homology, humans and mice are known to not respond completely alike to interventions/gene KO. This could also explain the differences in expressivity between the renal mouse and human phenotypes in this study (milder in mice than in affected individuals).^53^

In Northern blot experiments, mRNA transcripts of *Foxd2* were detected in the kidney, facial regions (tongue, nose, and maxilla), and brain.^51^ Developmental delay presenting as delayed motor milestone achievement and delayed speech development was noted in the affected individuals of families 1 and 2 (see clinical case in the Results section and Supplementary Case Report). Unfortunately, no cranial magnetic resonance imaging data were available except for individual II-1 of family 2, in whom enlarged ventricles and an enlarged subarachnoid space were noted at the age of 13 years (see Supplementary Case Report). Furthermore, facial anomalies in affected individuals were compatible with the previously detected expression patterns. A neurological phenotype was not reported in *Foxd2* KO mice in the literature. However, *Foxd1* KO mice present with small cerebral hemispheres with reduced development of the ventral telencephalon.^10^^,^^54^^,^^55^ Facial alterations, in turn, were not reported in *Foxd2* KO mice in the literature, but could be detected in the meticulously phenotyped *Foxd2* KO mice of this study (Figure 3a–d), in line with the facial dysmorphies of the affected individuals of families 1 to 3 (Figure 1e–h and Table 1).^10^^,^^51^^,^^56^^,^^57^ Furthermore, behavioral changes were identified in *Foxd2* KO mice of this study (hypoactivity in the open field test; Supplementary Figure S6). This can be indicative of neurodevelopmental alterations in *Foxd2* KO mice. Taken together, it can be assumed that *FOXD2* plays an important role in neuronal, branchial arch, and facial development.

Transcriptome analysis of mk4 CRISPR/Cas9-mediated homozygous *Foxd2* frameshift mutants gave a valuable insight into the possible mechanisms of CAKUT in the described families. Pathway analysis showed significant enrichment of differentially expressed genes important for the development of the renal/urogenital system (Table 3). *Pax2* was downregulated in all mk4 clones (Figure 6a; Supplementary Figure S8C) as were Pax2 protein levels on the Western blot (Figure 6b and c). *PAX2* haploinsufficiency is known to be associated with papillorenal syndrome in humans (MIM #120330), which comprises a CAKUT phenotype with eye anomalies.^58^ Of note, no overt eye anomalies were reported in the affected individuals described in this study. However, there is *Foxd2* RNA expression around the eye vesicle in mice during embryonic development.^59^ In line with this, the generated *Foxd2* KO mice showed alterations of the optic disc and nerve (Figure 3e and f), which might have been missed on routine ophthalmological examination of the affected individuals. Interestingly, there were alterations on eye examination of individual II-1 of family 2 and in the index individual of family 3. However, these did not involve the optic nerve and disc (see clinical case in the Results section).

*Foxd2* is strongly expressed in renal condensed mesenchyme at embryonic day 11.5, similarly to *Pax2*.^10^ *Pax2* is activated in the mesenchyme in response to induction by the ureteric bud and is subsequently downregulated in more differentiated cells derived from the mesenchyme. With reduced Pax2 protein levels, kidney mesenchyme cells fail to aggregate and do not undergo the sequential morphological changes characteristic of epithelial cell formation, demonstrating an essential role for Pax2 function for early mesenchymal-epithelial transition.^60^ Interestingly, we also detected a downregulation of *Emx2* in *Foxd2* mutant versus control cells by transcriptomics. *Pax2* enhances *Emx2* gene expression, and digenic loss of function of *Pax2* and *Emx2* is known to result in CAKUT similar to what is found in *Foxd2* KO mice and to the phenotype observed in the affected individuals of this study.^10^^,^^61^

Intriguingly, *Pax2* is also implicated in establishing the nephron-interstitium boundary during kidney development: nephron progenitor cells lacking Pax2 fail to differentiate into nephron cells, but can switch fates into Foxd1-positive renal interstitium–like cell types, suggesting that Pax2 function maintains nephron progenitor cells by repressing a renal interstitial cell program.^62^ Our findings suggest that the lack of Foxd2 results in reduced Pax2 levels. Therefore, it seems possible that Foxd2 dysfunction will divert lineage identity toward renal stroma cells. In line with this hypothesis, we could detect significantly increased cytokeratin 8 expression in the renal cortex of homozygous *Foxd2* KO mice compared with wt controls (Figure 5; Supplementary Figure S5). Keratins such as cytokeratin 8 are markers of tubular epithelial injury preceding renal fibrotic changes.^63^ Fittingly, individual II-2 of family 2 featured fibrotic changes on kidney biopsy in terms of FSGS (Figure 1i).

This hypothesis is further supported by the upregulation of *Fat4* in *Foxd2* mutants (Supplementary Figure S8C). *Fat4* encodes an atypical cadherin expressed by stromal cells inhibiting nephron progenitor renewal.^64^ In contrast, there was a marked downregulation of *Fat2* in *Foxd2* mutant cells (Figure 6a; Supplementary Figure S8C). Although *Fat4* has been shown to play a role in kidney tubule elongation and planar cell polarity in renal cells, the role of *Fat2* in renal development and homeostasis is unclear.^65^ The *Drosophila* orthologue *fat* has been implicated in cell proliferation and morphogenesis in a contact-dependent manner.^66^ We also observed a strong upregulation of *Nfia* in *Foxd2* mutant cells (Figure 6a). Haploinsufficiency of *NFIA* has been associated with brain malformations and CAKUT, and upregulation could be a compensatory mechanism in *Foxd2* KO cells.^67^^,^^68^

In contrast, *Fgfr2* was downregulated in mutant *Foxd2* cells versus controls (Figure 6a). Conditional KO of *Fgfr2* in metanephric mesenchyme cells leads to CAKUT in mice including hypo-/dysplastic kidneys and hydroureters. Interestingly, in these mice devoid of mesenchymal *Fgfr2* expression, there is no Fgfr2 in stromal cells either, also indicating a disturbance in stromal cells in *Fgfr2* KO mice.^69^

**Figure 7:**
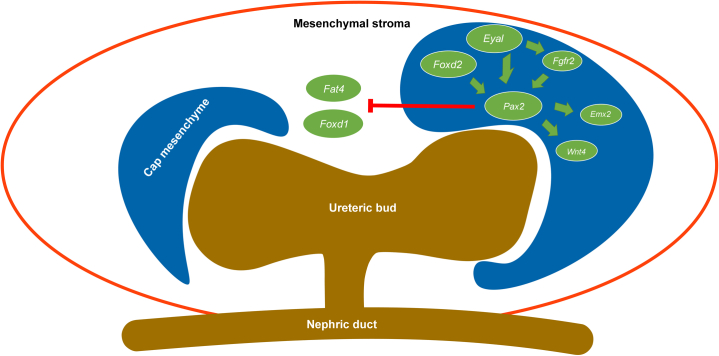
**Key players in the network of assumed Foxd2 (forkhead boxD2) function.** Although *Foxd1* expression in the developing mouse kidney is largely restricted to the mesenchymal stroma, *Foxd2* is found in the cap mesenchyme where it plays a role in *Pax2* expression.^10^^,^^74^ Foxd2 dysfunction leads to reduced PAX2 protein levels (see the Results section and Figure 6b and c). *Eya1* is important for *Pax2* expression in the ureteric bud–induced metanephric mesenchyme (embryonic day 11.5 in the mouse), and *Eya1* upregulation in *Foxd2* mutant cells may indicate a compensatory mechanism for reduced *Pax2* expression via a feedback loop.^76^*Pax2* activates *Wnt4* expression in the metanephric mesenchyme during mammalian kidney development, and *Wnt4* expression is downregulated in *Foxd2* mutant cells.^70^*Pax2* enhances *Emx2* gene expression, and *Emx2* expression is downregulated in *Foxd2* mutant cells.^61^ Nephron progenitor cells lacking Pax2 can change into Foxd1-positive renal interstitium–like cell types, suggesting that Pax2 represses a renal interstitial cell program.^62^ This is supported by the upregulation of *Fat4* in *Foxd2* mutant cells as *Fat4* encodes an atypical cadherin expressed by stromal cells inhibiting nephron progenitor renewal.^64^*Fgfr2* (and *Fgfr1*) is believed to act downstream of *Eya1* and upstream of *Pax2* in the metanephric mesenchyme.^77^ Conditional knockout of *Fgfr2* in metanephric mesenchyme cells leads to congenital anomalies of the kidney and urinary tract in mice and deficiency of Fgfr2 in stromal cells^69^ (see Discussion for further details). Adapted from McMahon AP. Development of the mammalian kidney. *Curr Top Dev Biol*. 2016;117:31–64^74^ and Walker KA, Sims-Lucas S, Bates CM. Fibroblast growth factor receptor signaling in kidney and lower urinary tract development. *Pediatr Nephrol*. 2016;31:885–895.^75^

Further, *Pax2* activates *Wnt4* expression in the metanephric mesenchyme during mammalian kidney development,^70^ potentially explaining reduced *Wnt4* gene expression in *Foxd2* mutant cells (Figure 6a; Supplementary Figure S8C). Wnt4 protein plays a role in mesenchymal-epithelial transition and is essential for tubulogenesis in the developing kidney through a noncanonical Wnt-signaling pathway.^71^^,^^72^ Of note, Wnt4 signaling can be substituted by other Wnt proteins such as Wnt7b,^73^ which was also downregulated in *Foxd2* mutant mk4 cells (Supplementary Figure S9B). Figure 7^10^^,^^61^^,^^62^^,^^64^^,^^69^^,^^70^^,^^74^, ^75^, ^76^, ^77^ summarizes the network of assumed Foxd2 function (adapted from McMahon^74^ and Walker *et al.*^75^; with the presumed feedback loop^76^).

In line with the hypothesis of an impaired Pax2-Wnt4 axis due to Foxd2 dysfunction abrogating proper mesenchymal-epithelial transition and tubulogenesis, we could indeed show significantly lower rates of tubule formation when conditioned medium from *Foxd2* mutant cells was applied compared with conditioned medium derived from wt cells in 3D tubuloid models (Supplementary Figure S10).

In addition, we observed higher *Eya1* expression in *Foxd2* mutant cells compared with control cells (Supplementary Figure S8C). *Eya1* is important for *Pax2* expression in the ureteric bud–induced metanephric mesenchyme, and *Eya1* upregulation in *Foxd2* mutant cells may indicate a compensatory mechanism for reduced *Pax2* expression via a feedback loop.^76^ *Gdnf* expression, which is vital for ureteric bud induction, was unchanged in *Foxd2* mutants (Supplementary Figure S9B). This is in line with *Eya1* upregulation in the context of reduced Pax2 levels, as both genes regulate *Gdnf* expression.^76^^,^^78^ Hence, it does not seem likely that the renal hypoplasia phenotype observed in the index individuals of the presented families is a result of impaired *Gdnf* expression.

This study has several limitations. The affected individuals of families 1 to 3 do not share the same type of variant. Family 1 segregates a homozygous frameshift variant in *FOXD2*, families 2 and 3 feature a homozygous missense variant affecting the same codon. However, as *FOXD2* is a single-exon gene (NM_004474.4), nonsense-mediated decay cannot be assumed, and this makes the frameshift variant not a clear-cut loss-of-function variant but probably leads to an altered protein. Interestingly, no homozygous truncating variants are listed in gnomAD, illustrating that there is constraint for this type of variant in *FOXD2*. This supports a causative role of the frameshift variant. Unfortunately, no patient-derived cells could be obtained in affected individuals of family 1 to clarify *FOXD2* expression. This limits the transferability of the KO experiments in this study. In contrast, it cannot be denied that *Foxd2* KO recapitulates the phenotype of the affected individuals of this study (all families 1–3). Furthermore, family 2 segregates a protein-altering variant—identified by a stringent filtering process—and features striking phenotypic overlap in affected individuals in comparison to family 1. In families 2 and 3, it could be shown by *in silico* analysis that the missense variant, located in the DNA-binding domain, destabilizes the mature protein. Hence, we believe that both missense variants abrogate proper FOXD2 function, which can be related to by KO experiments. Of course, further studies are needed to experimentally clarify causality of the described variants in *FOXD2* (according to MacArthur *et al.*^79^). Finally, a limitation of our cell culture model is that we investigated similar but not identical *FOXD2* variants *in vitro* compared with the variants we identified in human individuals with CAKUT.

In conclusion, our findings indicate that the syndromic CAKUT phenotype in the presented families is caused by FOXD2 dysfunction, putatively causing a shift of nephron progenitor cells undergoing mesenchymal-epithelial transition toward a stromal cell identity, resulting in fibrotic changes in the kidney. The observed human and *Foxd2* KO mouse phenotypes highlight an important role of *Foxd2* in kidney and craniofacial development. The observed kidney alterations are in line with the enrichment of differentially expressed genes important in extracellular matrix organization and renal/urogenital development in *Foxd2* mutant metanephric mesenchyme cells.

It is intriguing that FOXD2 dysfunction could result in a phenotype of both renal malformation and podocyte damage. As the *FOXD2* locus was also previously associated with urinary albumin in genome-wide meta-analyses^35^ and we have now identified a likely podocyte-specific regulatory SNP within this locus, FOXD2 could represent an interesting target in common kidney diseases in terms of tackling proteinuria and renal fibrosis. Consequently, our findings are building bridges between rare monogenic and common complex kidney disease.

## Disclosure

All the authors declared no competing interests.

## Data Statement

### Exome data

Human next-generation sequencing data sets for families 1 to 3 are not openly available, as the private nature of disease-causing *FOXD2* (forkhead box D2) variants in these data sets would make individuals identifiable. Data can be made available on personal request. Monogenic *FOXD2* disease alleles in families 1 to 3 and corresponding phenotypes have been submitted to ClinVar.

### Genome-wide association study analyses

The data supporting the findings of this study are described in Teumer *et al.*^35^ Genome-wide summary statistics from the publication are publicly available in repository https://ckdgen.imbi.uni-freiburg.de/. The data from the UK Biobank used in fine-mapping analyses were obtained and analyzed under application number 20272. All other analyses used publicly available data sets as described in the Methods section.

### Transcriptomics analyses in renal cells

The RNA sequencing data supporting the findings of this study are openly available in Gene Expression Omnibus (GEO; https://www.ncbi.nlm.nih.gov/geo/query/acc.cgi?acc=GSE168582). Codes for analyses and plotting are available under https://github.com/gwangjinkim/foxd2_analysis.

## Appendix

### Web resources

1000 Genomes Project human polymorphism database, http://www.1000genomes.org/

AlphaFold database, https://alphafold.ebi.ac.uk/entry/O60548

ANNOVAR, https://annovar.openbioinformatics.org/en/latest/

Burrows-Wheeler aligner, https://bio-bwa.sourceforge.net/

Combined Annotation Dependent Depletion (CADD) https://cadd.gs.washington.edu/

ClinPred, https://sites.google.com/site/clinpred/

ClinVar, https://www.ncbi.nlm.nih.gov/clinvar/

Clustal Omega, https://www.ebi.ac.uk/Tools/msa/clustalo/

Database of Genomic Variants (DGV) http://dgv.tcag.ca/dgv/app/home

dbSNP, http://www.ncbi.nlm.nih.gov/SNP/

DECIPHER, https://decipher.sanger.ac.uk/

DynaMut2, https://bio.tools/dynamut

Expression atlas, https://www.ebi.ac.uk/gxa/home

FinnGen release DF9, https://r9.finngen.fi/

FoldX, https://foldxsuite.crg.eu/

Galaxy platform12, https://usegalaxy.org/

Genome Analysis Tool Kit, https://gatk.broadinstitute.org/hc/en-us

Genome Aggregation Database (gnomAD v.2.1.1), http://gnomad.broadinstitute.org/

Homozygous Stretch Identifier (HomSI), https://bio.tools/homsi

Human Gene Mutation Database (HGMD®), http://www.hgmd.cf.ac.uk

Human Kidney snATAC-seq Peaks, http://www.susztaklab.com/human_kidney/igv/

Leiden Open Variation Database (LOVD), https://www.lovd.nl

INPS-3D, https://inpsmd.biocomp.unibo.it/inpsSuite/default/index3D

Mutation Taster, https://www.mutationtaster.org

MutPred2, http://mutpred.mutdb.org/

National Heart, Lung, and Blood Institute Exome Sequencing Project, http://evs.gs.washington.edu/EVS/

Online Mendelian Inheritance in Man®, http://www.omim.org/

Picard tool, https://broadinstitute.github.io/picard

Polymorphism Phenotyping v2 (PolyPhen-2), http://genetics.bwh.harvard.edu/pph2/

PremPS, https://bio.tools/premps

Protein Variation Effect Analyzer (PROVEAN), http://provean.jcvi.org/index.php/

Primer3, http://frodo.wi.mit.edu/primer3/input.htm

QuPath, https://QuPath.github.io/

Rare Exome Variant Ensemble Learner (REVEL), https://sites.google.com/site/revelgenomics/?pli=1

RCSB Protein Data Bank, https://www.rcsb.org/

Sorting Intolerant From Tolerant (SIFT), https://sift.bii.a-star.edu.sg

susieR, https://stephenslab.github.io/susieR/

The Human Protein Atlas, https://www.proteinatlas.org/

UK Biobank TOPMed-imputed PheWeb, https://pheweb.org/UKB-TOPMed/

UniProt, https://www.uniprot.org/

## Supplementary Material

Supplementary File (Word)

Supplementary File (Excel)

Supplementary File (Excel)

**Supplementary Methods.**

**Supplementary Results.** Supplementary Case Report Families 1, 2, and 3.

**Supplementary Figure S1.** Chromatograms of the homozygous frameshift variant NM_004474.4:c.789dup, p.(Gly264Argfs∗228) in *FOXD2* (forkhead box 2) of family 1. The wild-type reading frame in the chromatogram is CTACGGCG. Affected individuals carried the variant homozygously (VI-3 and V-19), whereas the healthy parents/relatives were heterozygous for the variant (V-6, V-1, IV-6, IV-4).

**Supplementary Figure S2.** (**A**) Homozygosity mapping of family 2. The overlapping homozygous stretches shared in 2 affected individuals (II-1 and II-2) are ∼11.6 Mb in size between genomic positions of 47,600,000 and 59,200,000 on chromosome 1 (box). *FOXD2* (forkhead box 2) resides here. (**B**) Chromatograms of the missense variant NM_004474.4:c.628 A>G), p.(Met210Val) observed in *FOXD2*. Affected individuals carried the variant homozygously, whereas the healthy parents were heterozygous for the variant. The unaffected sibling had a wild-type sequence.

**Supplementary Figure S3.** (**A**) Reverse transcription polymerase chain reaction and cDNA synthesis confirm the absence of *Foxd2* (forkhead box 2) transcript in homozygous (hom) knockout (KO) animals. (**B**) Decreased viability in hom *Foxd2* KO newborns. Pie charts representing different genotype proportions. The “expected” genotype percentages are those anticipated according to Mendelian distribution after double heterozygous (het) mating. The “born offspring” genotype proportions show the genotype proportions determined in pups born from such mating, and the “viable offspring” show the genotype percentages in offspring that survived weaning. Percentages were calculated by dividing the observed number of pups with the respective genotype by the total number of offspring (*n* = 143 born; *n* = 116 weaned) that was genotyped multiplied by 100. wt, wild type.

**Supplementary Figure S4.** Other histopathological renal alterations in 16-week-old mice. (**A**) Representative images of periodic acid–Schiff (PAS) staining of a wild-type (wt) mouse and a *Foxd2* (forkhead box 2) homozygous knockout (KO) mouse. No alterations such as thickening of the glomerular and tubular basement membrane or increased proliferation of mesangial cells or Bowman’s capsule are observed. (**B**) Hematoxylin and eosin (H&E) staining shows mild and focal alterations found only in homozygous KO mice. From left to right: examples of tubular basophilia, hyaline intratubular cast, and presence of clear vacuoles (arrows) in the renal tubular epithelium.

**Supplementary Figure S5.** (**A**) *Foxd2* (forkhead box 2) homozygous knockout (KO) increases cortical cytokeratin 8 (CK8) expression*. Foxd2* homozygous KO mice show highly increased CK8 expression. Data are expressed as mean ± SD. Two-sample *t* test, ∗∗∗*P* < 0.001. Representative examples of tissue image analysis CK8 staining of the renal cortex. Overview of (**B**) kidney with the marked cortex, section wild-type (**C**) preanalysis and (**D**) postanalysis, and homozygous KO mice (**E**) preanalysis and (**F**) postanalysis. Yellow indicates marked region; red, positive cells; blue, negative cells; and brown, CK8 H-DAB immunohistochemistry.

**Supplementary Figure S6.***Foxd2* (forkhead box 2) homozygous (hom) knockout (KO) causes hypoactivity and hypoexploration in a novel mildly stressful environment. *Foxd2* hom KO mice show clear (**A**) hypoactivity (decreased distance traveled) and (**B**) hypoexploration (decreased rearing) during the 20-minute open field test compared with wild-type (wt) controls at 8 weeks. Data are expressed as mean ± SD. ∗∗∗*P* < 0.001, genotype effect in 2-way analysis of variance.

**Supplementary Figure S7.** (**A**) Illustration of Foxd2 (forkhead box 2) protein showing *Foxd2* mutant clone F7. (**B**) Amino acid sequence of human wild-type (WT) *FOXD2* (NP_004465.3), human mutant *FOXD2* p.(Gly264Argfs∗228), mouse WT *Foxd2* (NP_032619.1), and mouse *Foxd2* clone F7.

**Supplementary Figure S8.** Transcriptome analysis results of *Foxd2* (forkhead box 2) mutant cells versus controls. (**A**) Heatmap GO term kidney morphogenesis. (**B**) Heatmap GO term mitogen-activated protein kinase (MAPK) signaling. (**C**) Quantitative polymerase chain reaction (PCR). Confirmation of the selected genes of interest from transcriptomics in all 4 different clones versus control cells using real-time PCR analysis (*y* axis: log 2 relative expression values [Log_2_RQ]). In agreement with RNA sequencing results, we could not detect differences in *Foxd1* gene expression; however, they confirmed downregulation of *Fgfr2*, *Wnt4*, *Fat2*, and *Pax2* and upregulation of *Eya1* and *Fat4* in *Foxd2* mutant versus control cells. Data shown for *n* = 1 biological experiment. Error bars indicate the SD of technical duplicates.

**Supplementary Figure S9.** (**A**) Pearson correlation of quantitative polymerase chain reaction (qPCR) validated genes showing a highly significant correlation with the relative expression levels determined by RNA sequencing (Pearson *R* = 0.8955; *P* < 0.0001). (**B**) qPCR validation of the selected genes of interest found to be differentially regulated by transcriptomics (*y* axis: log 2 relative expression values [Log_2_RQ]; see also Figure 6 in the main text).

**Supplementary Figure S10.** (**A**) Schematic overview of the CMUB-1 Matrigel culture. Cell culture medium from mk3 *Foxd2* (forkhead box 2) mutant or wild-type cells was concentrated by centrifugation and added to wild-type CMUB-1 cells in Matrigel on day 0 (“conditioned medium”). Normal (nonconditioned) medium containing additional GDNF and FGF1 served as a positive control. Differentiation of CMUB-1 cells into 3-dimensional (3D) tubuloids was visualized by fluorescence microscopy. Graphics created with BioRender.com. (**B–E**) Confocal images of 3D CMUB-1 cell culture shows formation of different types of 3D structures after 7 days of differentiation. A graphical abstract of structure types is presented in (**G**). Immunofluorescence was performed using the cell surface marker Na/K adenosine triphosphatase (Na/K-ATPase) or β-catenin (both in green), cytoskeleton marker F-actin (red), and nucleus marker 4ʹ,6-diamidino-2-phenylindole (DAPI; blue). Bar = 30 μm. (**B**) CMUB-1 cells under addition of GDNF/FGF1 (positive control). (i) Confocal overview image displaying different ureteric bud tubuloid types. The arrow indicates outgrowing tubule. The cross indicates lumen. (ii) Tubule with lumen (cross). (iii) The arrows mark round cyst with budding (top) and elongation (right). (**C**) Negative control, CMUB-1 cells plus normal culture medium barely showed tubule formation. (i) Overview image. (ii) Round cyst with lumen (cross). (**D**) Addition of wild-type conditioned medium resulted in tubule formation similar to what we observed upon addition of GDNF- and FGF-treated medium. (i) Overview image showing a tubule with elongation (arrow). (ii) Tubule with continuous lumen (cross) developed. (iii) Structures with several elongations (arrow) was observed. (**E**) Conditioned medium from *Foxd2* mutant cells resulted in reduced tubulogenesis and budding/elongation as compared with wild-type conditioned medium. (i) Overview image. (ii) Close-up of a cyst with lumen (cross) typically seen in ureteric bud culture plus mutant conditioned medium. (**F**) Statistical analysis of structure types observed. The positive control GDNF/FGF1 showed significantly increased tubule formation as well as elongations/buddings and protuberances compared with negative control and both mutant conditioned media. Negative control (normal medium) as well as both mutant conditioned media resulted in the formation of cysts/spheroids without signs of budding or elongation, while wild-type conditioned medium showed a significant increase in tubulogenesis, elongations/buddings, and protuberance formations compared with both mutant conditioned media and negative control. *n* = 3 independent experiments. Per experiment, 94 to 102 structures were counted. For *P*-value calculation, an unpaired Student *t* test was conducted. ∗*P* < 0.05. (**G**) Graphical description and definition of structures seen in ureteric bud 3D culture. (**H**) Diameter of structures of each condition was analyzed. GDNF/FGF1 treatment resulted in diameters of ∼50 μm, reflecting a collecting tubule diameter. Nonconditioned and mutant conditioned media resulted in diameters of ∼25 μm, while wild-type conditioned medium resulted in diameters of ∼35 μm. *n* = 2 independent experiments. Per experiment, 94 to 101 structures were counted. An unpaired Student *t* test was performed to analyze *P* value. ∗*P* < 0.05, ∗∗∗*P* < 0.001, ∗∗∗∗*P* < 0.0001. ns, not significant.

**Supplementary References.**

**Supplementary Table S2.** List of filtered candidate variants detected with exome sequencing in the index individual (II-1) of family 2.

**Supplementary Table S3.** Additional variants reported on next-generation sequencing in the index case of family 3.

**Supplementary Table S4.** Online *in silico* analysis tools used for evaluating protein stability changes on the p.(Met210Val) and p.(Met210Arg) missense variants in *FOXD2* (forkhead box 2), and Gibbs free energy calculation results. INPS-3D and DynaMut2: ΔΔ*G* < 0 is destabilizing; ΔΔ*G* > 0 is stabilizing PremPS and FoldX: ΔΔ*G* > 0 is destabilizing; ΔΔ*G* < 0 is stabilizing.

**Supplementary Table S6.** (**A,B**) Histological parameters of the examined kidneys. GM, glomeruli; H&E, hematoxylin and eosin; hom, homozygous knockout; NAD, no alteration determined; wt, wild type.

**Supplementary Table S7.** Total distance traveled and total rearing activity of wild-type (wt) control mice and heterozygous (het) and homozygous (hom) *Foxd2* (forkhead box 2) knockout mice.

**Supplementary Table S1,** Sheet 1. List of homozygous variants at minor allele frequency (MAF) 1.0% detected with exome sequencing in the index individual of family 1. Variants listed as “benign” or likely “benign” in ClinVar (https://www.ncbi.nlm.nih.gov/clinvar/) have been deleted for the sake of clarity. Only intronic variants ±20 bp to the exon-intron boundary are shown.

**Supplementary Table S1,** Sheet 2. List of rare homozygous variants detected with exome sequencing in the index individual of family 3. Filters applied included homozygosity in the proband but not the mother, absence from the gnomAD database, high or moderate quality as determined by the automated pipeline, and variant type that included missense, splice, or intronic variants.

**Supplementary Table S5** Summary statistics of fine-mapped urinary albumin-to-creatinine ratio (UACR)–associated variants at FOXD2 (forkhead box D2) locus in the UK Biobank and CKDGen Consortium.

## References

[1] Pohl M., Bhatnagar V., Mendoza S.A., Nigam S.K. (2002). Toward an etiological classification of developmental disorders of the kidney and upper urinary tract. Kidney Int.

[2] Chesnaye N., Bonthuis M., Schaefer F. (2014). Demographics of paediatric renal replacement therapy in Europe: a report of the ESPN/ERA-EDTA registry. Pediatr Nephrol.

[3] van der Ven A.T., Connaughton D.M., Ityel H. (2018). Whole-exome sequencing identifies causative mutations in families with congenital anomalies of the kidney and urinary tract. J Am Soc Nephrol.

[4] Verbitsky M., Westland R., Perez A. (2019). The copy number variation landscape of congenital anomalies of the kidney and urinary tract. Nat Genet.

[5] Vivante A., Mann N., Yonath H. (2017). A dominant mutation in nuclear receptor interacting protein 1 causes urinary tract malformations via dysregulation of retinoic acid signaling. J Am Soc Nephrol.

[6] Sanna-Cherchi S., Westland R., Ghiggeri G.M., Gharavi A.G. (2018). Genetic basis of human congenital anomalies of the kidney and urinary tract. J Clin Invest.

[7] Connaughton D.M., Hildebrandt F. (2022). Disease mechanisms of monogenic congenital anomalies of the kidney and urinary tract. Am J Med Genet C Semin Med Genet.

[8] Vivante A., Hwang D.Y., Kohl S. (2017). Exome sequencing discerns syndromes in patients from consanguineous families with congenital anomalies of the kidneys and urinary tract. J Am Soc Nephrol.

[9] Vivante A., Kohl S., Hwang D.Y. (2014). Single-gene causes of congenital anomalies of the kidney and urinary tract (CAKUT) in humans. Pediatr Nephrol.

[10] Kume T., Deng K., Hogan B.L. (2000). Minimal phenotype of mice homozygous for a null mutation in the forkhead/winged helix gene, Mf2. Mol Cell Biol.

[11] Kremer L.S., Bader D.M., Mertes C. (2017). Genetic diagnosis of Mendelian disorders via RNA sequencing. Nat Commun.

[12] Sobreira N., Schiettecatte F., Valle D., Hamosh A. (2015). GeneMatcher: a matching tool for connecting investigators with an interest in the same gene. Hum Mutat.

[13] Gormez Z., Bakir-Gungor B., Sagiroglu M.S. (2014). HomSI: a homozygous stretch identifier from next-generation sequencing data. Bioinformatics.

[14] Riedhammer K.M., Nguyen T.T., Kosukcu C. (2023). Implication of FOXD2 dysfunction in syndromic congenital anomalies of the kidney and urinary tract (CAKUT). *Preprint*. medRxiv.

[15] Ioannidis N.M., Rothstein J.H., Pejaver V. (2016). REVEL: an ensemble method for predicting the pathogenicity of rare missense variants. Am J Hum Genet.

[16] Alirezaie N., Kernohan K.D., Hartley T. (2018). ClinPred: prediction tool to identify disease-relevant nonsynonymous single-nucleotide variants. Am J Hum Genet.

[17] Pejaver V., Urresti J., Lugo-Martinez J. (2020). Inferring the molecular and phenotypic impact of amino acid variants with MutPred2. Nat Commun.

[18] Schymkowitz J., Borg J., Stricher F. (2005). The FoldX web server: an online force field. Nucleic Acids Res.

[19] Rodrigues C.H.M., Pires D.E.V., Ascher D.B. (2021). DynaMut2: assessing changes in stability and flexibility upon single and multiple point missense mutations. Protein Sci.

[20] Savojardo C., Fariselli P., Martelli P.L., Casadio R. (2016). INPS-MD: a web server to predict stability of protein variants from sequence and structure. Bioinformatics.

[21] Chen Y., Lu H., Zhang N. (2020). PremPS: predicting the impact of missense mutations on protein stability. PLoS Comput Biol.

[22] Haeussler M., Schonig K., Eckert H. (2016). Evaluation of off-target and on-target scoring algorithms and integration into the guide RNA selection tool CRISPOR. Genome Biol.

[23] Gailus-Durner V., Fuchs H., Becker L. (2005). Introducing the German Mouse Clinic: open access platform for standardized phenotyping. Nat Methods.

[24] Fuchs H., Aguilar-Pimentel J.A., Amarie O.V. (2018). Understanding gene functions and disease mechanisms: phenotyping pipelines in the German Mouse Clinic. Behav Brain Res.

[25] Fuchs H., Gailus-Durner V., Adler T. (2009). The German Mouse Clinic: a platform for systemic phenotype analysis of mouse models. Curr Pharm Biotechnol.

[26] Rathkolb B., Hans W., Prehn C. (2013). Clinical chemistry and other laboratory tests on mouse plasma or serum. Curr Protoc Mouse Biol.

[27] Fuchs H., Gailus-Durner V., Adler T. (2011). Mouse phenotyping. Methods.

[28] Puk O., de Angelis M.H., Graw J. (2013). Longitudinal fundus and retinal studies with SD-OCT: a comparison of five mouse inbred strains. Mamm Genome.

[29] Holter S.M., Garrett L., Einicke J. (2015). Assessing cognition in mice. Curr Protoc Mouse Biol.

[30] Oud M.M., Latour B.L., Bakey Z. (2018). Cellular ciliary phenotyping indicates pathogenicity of novel variants in IFT140 and confirms a Mainzer-Saldino syndrome diagnosis. Cilia.

[31] Mohammed S.G., Arjona F.J., Verschuren E.H.J. (2018). Primary cilia-regulated transcriptome in the renal collecting duct. FASEB J.

[32] Tosic J., Kim G.J., Pavlovic M. (2019). Eomes and Brachyury control pluripotency exit and germ-layer segregation by changing the chromatin state. Nat Cell Biol.

[33] Rehman A.U., Najafi M., Kambouris M. (2019). Biallelic loss of function variants in PPP1R21 cause a neurodevelopmental syndrome with impaired endocytic function. Hum Mutat.

[34] Loges N.T., Antony D., Maver A. (2018). Recessive DNAH9 loss-of-function mutations cause laterality defects and subtle respiratory ciliary-beating defects. Am J Hum Genet.

[35] Teumer A., Li Y., Ghasemi S. (2019). Genome-wide association meta-analyses and fine-mapping elucidate pathways influencing albuminuria. Nat Commun.

[36] Zou Y., Carbonetto P., Wang G., Stephens M. (2022). Fine-mapping from summary data with the “Sum of Single Effects” model. PLoS Genet.

[37] Sheng X., Guan Y., Ma Z. (2021). Mapping the genetic architecture of human traits to cell types in the kidney identifies mechanisms of disease and potential treatments. Nat Genet.

[38] Pruim R.J., Welch R.P., Sanna S. (2010). LocusZoom: regional visualization of genome-wide association scan results. Bioinformatics.

[39] Muto Y., Wilson P.C., Ledru N. (2021). Single cell transcriptional and chromatin accessibility profiling redefine cellular heterogeneity in the adult human kidney. Nat Commun.

[40] Bycroft C., Freeman C., Petkova D. (2018). The UK Biobank resource with deep phenotyping and genomic data. Nature.

[41] Kurki M.I., Karjalainen J., Palta P. (2023). FinnGen provides genetic insights from a well-phenotyped isolated population. Nature.

[42] Lake B.B., Menon R., Winfree S. (2023). An atlas of healthy and injured cell states and niches in the human kidney. Nature.

[43] Schwartz G.J., Munoz A., Schneider M.F. (2009). New equations to estimate GFR in children with CKD. J Am Soc Nephrol.

[44] Fabre P.H., Herrel A., Fitriana Y. (2017). Masticatory muscle architecture in a water-rat from Australasia (Murinae, Hydromys) and its implication for the evolution of carnivory in rodents. J Anat.

[45] Carlsson P., Mahlapuu M. (2002). Forkhead transcription factors: key players in development and metabolism. Dev Biol.

[46] Benayoun B.A., Caburet S., Veitia R.A. (2011). Forkhead transcription factors: key players in health and disease. Trends Genet.

[47] Wu C.W., Mann N., Nakayama M. (2020). Phenotype expansion of heterozygous FOXC1 pathogenic variants toward involvement of congenital anomalies of the kidneys and urinary tract (CAKUT). Genet Med.

[48] Zheng B., Seltzsam S., Wang C. (2022). Whole exome sequencing identifies FOXL2, FOXA2 and FOXA3 as candidate genes for monogenic congenital anomalies of the kidneys and urinary tract. Nephrol Dial Transplant.

[49] Ernstsson S., Betz R., Lagercrantz S. (1997). Cloning and characterization of freac-9 (FKHL17), a novel kidney-expressed human forkhead gene that maps to chromosome 1p32-p34. Genomics.

[50] Okabe M., Motojima M., Miyazaki Y. (2019). Global polysome analysis of normal and injured podocytes. Am J Physiol Renal Physiol.

[51] Wu S.C., Grindley J., Winnier G.E. (1998). Mouse mesenchyme forkhead 2 (Mf2): expression, DNA binding and induction by sonic hedgehog during somitogenesis. Mech Dev.

[52] Hatini V., Huh S.O., Herzlinger D. (1996). Essential role of stromal mesenchyme in kidney morphogenesis revealed by targeted disruption of Winged Helix transcription factor BF-2. Genes Dev.

[53] Perlman R.L. (2016). Mouse models of human disease: an evolutionary perspective. Evol Med Public Health.

[54] Newman E.A., Kim D.W., Wan J. (2018). Foxd1 is required for terminal differentiation of anterior hypothalamic neuronal subtypes. Dev Biol.

[55] Xuan S., Baptista C.A., Balas G. (1995). Winged helix transcription factor BF-1 is essential for the development of the cerebral hemispheres. Neuron.

[56] Millington G., Elliott K.H., Chang Y.T. (2017). Cilia-dependent GLI processing in neural crest cells is required for tongue development. Dev Biol.

[57] Jeong J., Mao J., Tenzen T. (2004). Hedgehog signaling in the neural crest cells regulates the patterning and growth of facial primordia. Genes Dev.

[58] Sanyanusin P., Schimmenti L.A., McNoe L.A. (1995). Mutation of the PAX2 gene in a family with optic nerve colobomas, renal anomalies and vesicoureteral reflux. Nat Genet.

[59] Sasaki H., Hogan B.L. (1993). Differential expression of multiple fork head related genes during gastrulation and axial pattern formation in the mouse embryo. Development.

[60] Rothenpieler U.W., Dressler G.R. (1993). Pax-2 is required for mesenchyme-to-epithelium conversion during kidney development. Development.

[61] Boualia S.K., Gaitan Y., Murawski I. (2011). Vesicoureteral reflux and other urinary tract malformations in mice compound heterozygous for Pax2 and Emx2. PLoS One.

[62] Naiman N., Fujioka K., Fujino M. (2017). Repression of interstitial identity in nephron progenitor cells by Pax2 establishes the nephron-interstitium boundary during kidney development. Dev Cell.

[63] Djudjaj S., Papasotiriou M., Bulow R.D. (2016). Keratins are novel markers of renal epithelial cell injury. Kidney Int.

[64] Das A., Tanigawa S., Karner C.M. (2013). Stromal-epithelial crosstalk regulates kidney progenitor cell differentiation. Nat Cell Biol.

[65] Saburi S., Hester I., Fischer E. (2008). Loss of Fat4 disrupts PCP signaling and oriented cell division and leads to cystic kidney disease. Nat Genet.

[66] Saburi S., Hester I., Goodrich L., McNeill H. (2012). Functional interactions between Fat family cadherins in tissue morphogenesis and planar polarity. Development.

[67] Rao A., O’Donnell S., Bain N. (2014). An intragenic deletion of the NFIA gene in a patient with a hypoplastic corpus callosum, craniofacial abnormalities and urinary tract defects. Eur J Med Genet.

[68] Nyboe D., Kreiborg S., Kirchhoff M., Hove H.B. (2015). Familial craniosynostosis associated with a microdeletion involving the NFIA gene. Clin Dysmorphol.

[69] Hains D., Sims-Lucas S., Kish K. (2008). Role of fibroblast growth factor receptor 2 in kidney mesenchyme. Pediatr Res.

[70] Torban E., Dziarmaga A., Iglesias D. (2006). PAX2 activates WNT4 expression during mammalian kidney development. J Biol Chem.

[71] Tanigawa S., Wang H., Yang Y. (2011). Wnt4 induces nephronic tubules in metanephric mesenchyme by a non-canonical mechanism. Dev Biol.

[72] Stark K., Vainio S., Vassileva G., McMahon A.P. (1994). Epithelial transformation of metanephric mesenchyme in the developing kidney regulated by Wnt-4. Nature.

[73] Kispert A., Vainio S., McMahon A.P. (1998). Wnt-4 is a mesenchymal signal for epithelial transformation of metanephric mesenchyme in the developing kidney. Development.

[74] McMahon A.P. (2016). Development of the mammalian kidney. Curr Top Dev Biol.

[75] Walker K.A., Sims-Lucas S., Bates C.M. (2016). Fibroblast growth factor receptor signaling in kidney and lower urinary tract development. Pediatr Nephrol.

[76] Sajithlal G., Zou D., Silvius D., Xu P.X. (2005). Eya 1 acts as a critical regulator for specifying the metanephric mesenchyme. Dev Biol.

[77] Poladia D.P., Kish K., Kutay B. (2006). Role of fibroblast growth factor receptors 1 and 2 in the metanephric mesenchyme. Dev Biol.

[78] Brophy P.D., Ostrom L., Lang K.M., Dressler G.R. (2001). Regulation of ureteric bud outgrowth by Pax2-dependent activation of the glial derived neurotrophic factor gene. Development.

[79] MacArthur D.G., Manolio T.A., Dimmock D.P. (2014). Guidelines for investigating causality of sequence variants in human disease. Nature.

