# Supplementary material for: Implication of transcription factor FOXD2 dysfunction in syndromic congenital anomalies of the kidney and urinary tract (CAKUT)
Source: Kidney Int. 2024 Apr;105(4):844–64. doi: 10.1016/j.kint.2023.11.032 (PMC10957342; doi:10.1016/j.kint.2023.11.032)
Supplement: Supplementary File (Word) [file mmc1.docx]

**Implication of *FOXD2* dysfunction in syndromic congenital anomalies of the kidney and urinary tract (CAKUT)**

**Supplementary Material**

**Methods**

***Ascertainment of families*** ***4***

***Exome sequencing family 1 4***

***Sanger sequencing family 1 6***

***Exome sequencing family 2 7***

***Sanger sequencing family 2 9***

***Exome sequencing family 3 10***

***Protein Modelling and Gibbs Free Energy Calculation of FOXD2 p.(Met210Val) variant 11***

***CRISPR/Cas9 gene editing in mice 12***

***Mice 13***

***Mouse phenotyping 13***

***Image analysis of CK8-immunohistochemistry 15***

***Statistical analysis (mouse experiments) 16***

***Cell culture and CRISPR/Cas9 mutant generation (Foxd2 deficient metanephric mesenchyme cell models*) *16***

***RNA sequencing 17***

***RNA seq bioinformatics 18***

***Heatmaps 18***

***GO enrichment analysis 19***

***Volcano plot 19***

***PAX2 immunofluorescence 19***

***Western Blot analysis 20***

**Results**

***Supplementary Case Report Family 1 21***

***Supplementary Case Report Family 2 22***

***Supplementary Case Report Family 3 25***

**Figures and Tables**

***Supplementary Figure 1 27***

***Supplementary Figure 2 28***

***Supplementary Figure 3 29***

***Supplementary Figure 4 30***

***Supplementary Figure 5 31***

***Supplementary Figure 6 32***

***Supplementary Figure 7 33***

***Supplementary Figure 8*** ***34***

***Supplementary Figure 9*** ***35***

***Supplementary Figure 10*** ***36***

***Supplementary Table 1 38***

***Supplementary Table 2 39***

***Supplementary Table 3 40***

***Supplementary Table 4 41***

***Supplementary Table 5 42***

***Supplementary Table 6 43***

***Supplementary Table 7 44***

**References** ***45***

**Methods**

***Ascertainment of the families***

The study was approved by the local Ethics Committee of the Technical University of Munich, the Istanbul University-Cerrahpasa, Cerrahpasa Medical Faculty (No: 139896, Date: 22/10/2020), and the Rabin Medical Center (RMC) institutional review board committee in Petah Tikva (6826) and was performed according to the standard of the Helsinki Declaration of 2013. Written informed consent was obtained from all participants or their legal guardians. Explicit permission was obtained to publish the photographs of the Turkish individuals. Blood samples were collected after written informed consent was given from the individuals and/or their legal guardians. After informed consent, we also obtained clinical data and pedigree information from included families using a standardized questionnaire. As for the CAKUT cases of the NephroGen cohort (*n* = 313 families) and Turkish familial CAKUT cohort (*n* = 42 families)*,* the diagnosis was made by (pediatric) nephrologists on the basis of relevant imaging. The proband in family 3 was followed up at Schneider Children’s Medical Center of Israel by a multidisciplinary team due to her complex medical condition resulting from chronic renal failure and nephrotic syndrome. She was evaluated by a pediatric geneticist, including detailed medical history, pedigree and physical exam. She underwent clinical exome sequencing as part of her diagnostic workup. The study was approved by the Rabin Medical Center ethics committee. Family provided written informed consent for participation in the study and publication of medical data and pedigree.

***Exome sequencing family 1***

We investigated family members from a multiplex consanguineous Arabic family (Figure 1A) with a possible autosomal recessive mode of inheritance of syndromal CAKUT characterized predominantly by renal hypoplasia, developmental delay, and facial anomalies (mandibular hypoplasia, facial dysmorphic features; see Results). DNA sample of the index case (Figure 1A: VI-3) was analyzed by exome sequencing (ES), an also affected female first cousin once removed (Figure 1A: V-19) and the parents of both individuals (Figure 1A: V-1, V-6, IV-6; except the father of V-19) as well as the maternal grandmother of the index case (Figure 1A: IV-4) were analyzed by targeted Sanger sequencing.

ES was performed using a Sure Select Human All Exon 60 Mb V6 Kit (Agilent, Santa Clara, CA, USA) and a HiSeq4000 (Illumina, San Diego, CA, USA) as previously described.^1^ Mitochondrial DNA was derived from off-target exome reads as previously described.^2^ Reads were aligned to the human reference genome (UCSC Genome Browser build hg19) using Burrows-Wheeler Aligner (v.0.7.5a). Detection of single-nucleotide variants (SNVs) and small insertions and deletions (indels) was performed with SAMtools (version 0.1.19). ExomeDepth was used for the detection of CNVs. A noise threshold of 2.5 was accepted for diagnostic analysis.^3^ The exome of the index individual was first searched for causative variants in known disease-associated genes. Called CNVs were visualized by the Integrative Genomics Viewer (IGV, https://software.broadinstitute.org/software/igv/)^4^ to check for sufficient coverage of the inspected region and plausibility of the CNV. CNVs were compared with publicly available control databases like the Genome Aggregation Database (gnomAD, v.2.1.1, https://gnomad.broadinstitute.org/about), the Database of Genomic Variants (DGV, http://dgv.tcag.ca/dgv/app/home) and databases for pathogenic CNVs like DECIPHER (https://decipher.sanger.ac.uk/) and ClinVar (https://www.ncbi.nlm.nih.gov/clinvar/). For the analysis of *de novo,* autosomal dominant and mitochondrial SNVs and indels (in the following called “variants”), only variants with a minor allele frequency (MAF) of less than 0.1 % in the in-house database of the Helmholtz center Munich containing over 20,000 exomes were considered. For the analysis of autosomal recessive and X-linked variants (homozygous, hemizygous or putative compound heterozygous), variants with a MAF of less than 1.0 % were considered. Identified variants in known disease-associated genes were compared with publicly available databases for pathogenic variants like ClinVar, the Human Gene Mutation Database (HGMD®, http://www.hgmd.cf.ac.uk) and the Leiden Open Variation Database (LOVD, https://www.lovd.nl). Rating of SNVs/indels and CNVs in known disease-associated genes was done according to American College of Medical Genetics (ACMG) and additional guidelines.^5-7^

As no causative variant was found in known disease-associated genes and no causative CNV could be identified, search was extended for non-synonymous variants (missense, frameshift, nonsense, stop-loss, canonical splice site variants, in-frame indels) and CNVs in genes not yet associated with a monogenic disease using the same MAF thresholds as described above. The homozygous frameshift variant in *FOXD2* was prioritized because of the genotypic evidence (homozygous putative loss-of-function variant), its non-occurrence in databases like gnomAD and an in-house database of over 20,000 exomes (“Munich Exome Server”) and its implication in CAKUT as per a knockout (KO) mouse model.^8^ See identified homozygous variants at MAF 1.0% listed in Supplementary Table 1.

***Sanger sequencing family 1***

Segregation analysis was performed using direct DNA sequencing on both strands on an ABI capillary sequencer 3730 (Applied Biosystems, Foster City, CA, USA). Primers were designed by Primer3 program (http://frodo.wi.mit.edu/primer3/input.htm). DNA alignment and sequence variant analysis were carried out using the Sequence PilotCE software (JSI Medical Systems, Kippenheim, Germany) and compared to EMBL (European Molecular Biology Laboratory) and GenBank databases as well as our in-house database.

***Exome sequencing family 2***

In a consanguineous Turkish family (Figure 1B), we ascertained 2 siblings with a possible autosomal recessive mode of inheritance of syndromic CAKUT characterized predominantly by renal hypoplasia, neurodevelopmental delay, and facial anomalies (mandibular hypoplasia, facial dysmorphic features; see Results, Figure 1E-H, and Table 1).

We performed ES for index case (II-1), affected and unaffected siblings (II-2 and II-3) as well as healthy consanguineous parents (I-1 and I-2), respectively.

A total amount of 0.6 µg genomic DNA per sample was used as input material for the DNA sample preparation. Sequencing libraries were generated using Human Core Exome Panel v3.0 (Twist Bioscience, San Francisco, USA) following the manufacturer’s recommendations and index codes were added to each sample. Briefly, fragmentation was carried out by enzymology shearing to generate 150-350 bp fragments. Remaining overhangs were converted into blunt ends via exonuclease/polymerase activities. After adenylation of 3’ ends of DNA fragments, adapter oligonucleotides were ligated. DNA fragments with ligated adapter molecules on both ends were selectively enriched in a PCR reaction. After PCR reaction, libraries were hybridized with liquid phase with biotin labeled probe, then magnetic beads with streptomycin were used to capture the exons of genes. Captured libraries were enriched in a PCR reaction to add index tags to prepare for sequencing. Products were purified using AMPure XP system (Beckman Coulter, Brea, CA, USA) and were quantified using the Agilent high sensitivity DNA assay on the Agilent Bioanalyzer 2100 system (Agilent, Santa Clara, CA, USA).

After cluster generation, the DNA libraries were sequenced on MGI-T7 (MGI Tech, Shenzhen, China) sequencing system according to the manufacturer’s instructions. 150 bp paired-end reads were generated. Reads were mapped against the human reference genome assembly (GRCH37, hg19).

A custom bioinformatics pipeline was used for converting the MGI-formatted headers to Illumina-formatted headers (bash scripts are available upon request). Read qualities were checked with FastQC, and Trimmomatic software was used for trimming low quality sequences and removing adapters.^9^ The alignment of paired-end DNA sequence data with human reference genome hg19 (UCSC) was performed by using Burrows–Wheeler aligner (BWA) v0.7.17.18.^10^ Picard tool (https://broadinstitute.github.io/picard) was used to PCR duplicate removal. Variant calling was performed using Genome Analysis Tool Kit (GATK v3.7; https://gatk.broadinstitute.org/hc/en-us) and ANNOVAR software was used to functionally annotate genetic variants. Mean region coverage depth was 271.4X and 92% of the target region was at least 20% of the mean read depth for four samples. A total of 38,312, 39,335, 38,861, and 38,808 variants were annotated for the samples I-1, I-2, II-1, and II-2, respectively. Filtering steps following IGV^4^ visualization revealed three homozygous rare missense variants (i.e. *FOXD2, GALNT14* and *BIRC6*; Supplementary Table 2). None of them were detected in gnomAD in a homozygous state. Among them, two variants were classified as “disease causing”, “deleterious”, or “damaging by four different variant effect prediction methods (SIFT, PROVEAN, PolyPhen2, and MutationTaster). Of them, the pathogenicity scores were above threshold for the homozygous NM_004474.4; c.628A>G; p.(Met210Val) variant in *FOXD2* (CADD, Revel, ClinPred, and MutPred2).

Homozygosity mapping was performed using ES data via HomSI software (Homozygous Stretch Identifier from next-generation sequencing data), which was developed by Advanced Genomics and Bioinformatics Research Center in the Scientific and Technological Research Council of Türkiye.^11^

We focused on homozygous alterations in ES data as a result of pedigree evaluation and parental consanguinity (Figure 1B). Homozygosity mapping conducted with ES data revealed a ROH located in Chromosome 1, approximately 11.6 Mb-long (chr1: 47,607,000-59,155,000). The identified homozygous stretch showed that both affected siblings were sharing the ROH, however parents and the unaffected sibling were heterozygous for the region of interest (Supplementary Figure 2A). The homozygous missense p.(Met210Val) variant in *FOXD2*, which is located within the disease-segregating homozygous region, was selected for further evaluation with *in slico* methods.

***Sanger sequencing family 2***

Sanger sequencing was performed in all individuals of the family to validate the sequence variation identified by ES at Hacettepe University Nephrogenetics Laboratory, Ankara, Türkiye. Forward (5’-CAACGACTGCTTCGTCAAGA-3’) and reverse primers (5’-CCGTAGCCGTAGCCGTAG-3’) for exon 1 of *FOXD2* were designed, and the corresponding region was sequenced using the BigDye terminator v.3.1 sequencing kit and the ABI 3130 genetic analyzer (Applied Biosystems, Foster City, CA, USA). The GeneBank (National Center for Biotechnology Information, NCBI) sequence *FOXD2* NM_004474.4 (corresponding to Ensembl reference ENST00000334793.6) was used as reference sequence.

Conventional sequencing results confirmed the p.(Met210Val) variant in *FOXD2* is co-segregating with the disease in the family. Index individual and her affected older sister (II-1 and II-2) were homozygous for c.628A>G, p.(Met210Val) variant in *FOXD2*. The parents (I-1 and I-2) were heterozygous carriers, and the healthy younger sibling (II-3) was not carrying the alternative allele (Supplementary Figure 2B). These findings were consistent with ES results and validated the segregation across family members. The causative homozygous rare missense variant is located at the methionine residue at position 210, which is completely conserved among different species until the level of *Danio rerio* (Figure 1K).

***Exome sequencing family 3***

A seven year old girl from a consanguineous Bedouin family was referred to the Pediatric Genetics Unit at Schneider Children’s Medical Center of Israel for investigation of a renal disorder with prenatal onset. She presented with dysplastic kidneys, nephrotic syndrome and progressive renal failure, ultimately requiring kidney transplantation. In addition, she was noted to have speech delay, microcephaly and dysmorphic facial features (see Results). Her parents are first cousins.

Chromosomal microarray analysis was carried out using CytoScanTM 750K Array (Applied Biosystems) and analyzed at the Raphael Recanati Genetics Institute, Israel. The index case also underwent multi-gene panel testing (Blueprint Genetics, Helsinki, Finland), which was non-diagnostic. Subsequently, clinical duo ES (proband and mother) was performed.

Sequencing was done at The Genetics Institute in the Tel Aviv Sourasky Medical Center. Targeted capture of protein-coding regions was performed using xGen Exome Hyb Panel v2 (Integrated DNA Technologies). Paired-end libraries were sequenced on a NovaSeq 6000 (Illumina). At least 97% of target bases were covered at 20x or greater (95% at >100x). Data analysis was carried out at the Raphael Recanati Genetics Institute, Israel using two platforms: the first is Emedgene’s platform (Emedgene Technologies, Mazor, Israel). Variant alignment and detection were performed using DRAGEN bioinformatics pipeline (Illumina), with GRCh37/hg19 as the human genome reference. All called variants were assigned a quality value for filtering. Based on validation studies, the pipeline showed precision and detection >99% for SNVs in areas with coverage greater than 20x and high mapping quality. The following public and internal bioinformatics/genetics resources were used for variant annotation: gnomAD, Ensembl Variant Effect Predictor (VEP), dbNSFP, Emedgene© DB, SnpSift, SnpEff ExAC, GWAS GRC, ClinVar, Breast Cancer Information Core (BIC), BRCA Exchange, The Greater Middle East (GME), Amish Coriell and Mennonite, dbSNP, GERP, 1000 Genomes, Online Mendelian Inheritance in Man® (OMIM), Clinical Genomic Database (CGD), Clinical Genomic Database (CGD), Orphanet, and Emedgene© Gene-Disease Knowledge Graph. Tertiary analysis was performed using Emedgene's automated variant classification and prioritization application that uses an automated machine learning engine and a knowledge graph containing gene-disease relationships and polymorphism variant information to generate a shortlist of potential causative variants along with supporting evidence.

In addition, the Franklin genetic analysis and variant classification platform (https://franklin.genoox.com - Franklin by Genoox) was also used for variant analysis and prioritization, as previously described.^12^ FASTQ files were aligned against the GRCh37/hg19 reference genome using BWA version 0.7.17.^13^ GATK version 4.0.12.0 and FreeBayes version 1.3.1 were used for variant calling of single nucleotide variants and indels.^14^ Genoox Data Annotation Version 50 was used.

Interpretation and classification were based on the ACMG standards and guidelines.^5^

Since no causative variant was identified, the data was subsequently reanalyzed for suspicious rare homozygous variants, and a homozygous missense variant in *FOXD2* NM_004474.4:c.629T>G, p.(Met210Arg) was prioritized. While this manuscript was in preparation, a preprint version describing clinical and molecular findings in family 1 and family 2 was published on medRxiv, allowing us to identify the resemblance between the cases.^15^

Confirmation by Sanger sequencing and testing the siblings for the *FOXD2* variant was recommended to the family.

***Protein Modelling and Gibbs Free Energy Calculation of FOXD2* p.(Met210Val) and p.(Met210Arg) *variants***

The FOXD2 protein structure and PDB files were absent in the RCSB Protein Data Bank (https://www.rcsb.org/). Therefore, we used the pre-existing model based in AlphaFold database (https://alphafold.ebi.ac.uk/entry/O60548). Gibbs free energy minimization of the wild type protein structure was carried out with RepairPDB command of FoldX software^16^ with the following syntax:

foldx --command=RepairPDB --pdb= AF-O60548-F1-model_v2.pdb --water=CRYSTAL.

Four *in silico* tools, which estimate the impact of missense variants on protein stability, and calculates the changes of unfolding Gibbs free energy, ΔΔG were employed. Protein stability predictions were calculated with DynaMut2^17^, INPS-3D^18^, FoldX^16^ and PremPS^19^ using the AlphaFold structure of FOXD2 as input PDB file.

The ΔΔG value between the wild type protein and results of altered amino acid substitution were calculated for the p.(Met210Val) and p.(Met210Arg) variants in FOXD2. Finally, 3D structures of the wild type and altered FOXD2 protein structures were generated with DynaMut2 (Figure 1L). The ΔΔG values (kcal/mol) for both variants were interpreted as follows: Predicted ΔΔG<0 is destabilizing in the case of DynaMut2 and INPS-3D. Predicted ΔΔG>0 is destabilizing in the case of FoldX 5.0 and PremPS.

***CRISPR/Cas9 gene editing in mice***

The *Foxd2* KO mouse model was derived using CRISPR/Cas9 technology and systematically characterized in the German Mouse Clinic (GMC) phenotyping screen as described previously.^20,21^

To generate a knock out for the single-exon gene *Foxd2* the web-based CRISPOR Design Tool^22^ was used and 2 guides targeting exon 1 (gene ID 2306) were chosen resulting in an intra exon deletion of 1126 bp. The sgRNAs were synthesized by using the *in vitro* transcription EnGen Kit (New England Biolabs, Ipswich, MA, USA). Following *in vitro* transcription, the purified RNA was used for the deletion allele injection mix consisted of 200 ng/μL Cas9 protein from IDT (1081059) and 50 ng/μL sgRNA (each) in a final volume of 5 μL of 1x OptiMEM (Thermo Fischer Scientific, Waltham, MA, USA).

For zygote production C57BL/6NCrl female mice (32 d old) were superovulated, mated to C57BL/6NCrl males, and zygotes were collected at 0.5 dpc. The sgRNA/Cas9 mixture was electroporated into pronuclear stage zygotes. For a single electroporation event 50 one-cell stage embryos were filled in a glass chamber (1 mm) of a NEPA 21 electroporator (NEPA GENE, Ichikawa, Japan), filled with 5 µl OptiMEM containing sgRNA. RNP complexes following a standardized electroporation protocol. Resulting 2-cell embryos were transferred into pseudopregnant (day 0.5pc) CD-1-females. For genotype analysis, PCR reaction was performed with *Foxd2* specific primers (*Foxd2*_F ctgctgctgcgagatcatgtc and *Foxd2*_R tagactccctgaattccgctg).

***Mice***

Mice were maintained in IVC cages with water and standard mouse chow according to the directive 2010/63/EU, German laws, and GMC housing conditions. All tests were approved by the responsible authority of the district government of Upper Bavaria.

***Mouse phenotyping***

Homozygous *Foxd2* KO mice (10 males, 7 females), heterozygous *Foxd2* KO mice (8 males, 10 females) and wild type controls (33 males, 36 females; 6/69 were littermates, 63/69 mice of the same background strain) were analyzed by the German Mouse Clinic (GMC) at the Helmholtz Zentrum München, Neuherberg, Germany (http://www.mouseclinic.de).^20,21,23^ The phenotypic tests were part of the GMC screen and performed according to standardized protocols as described before.^24-27^ Variations in protocols are specified.

Eye posterior segment was imaged with a Spectralis OCT (Heidelberg Engineering, Heidelberg, Germany) and was carried out as described previously.^26^ The parameters evaluated were number of main blood vessels (fundus), retinal thickness, and morphology of retinal layers and optical disc.

The open field (OF) analysis was carried out as described previously using the ActiMot system (TSE Systems, Bad Homburg vor der Höhe, Germany).^28^ Arena illumination levels were set at ∼150 lux in the corners and 200 lux in the middle.

Blood samples collected by retrobulbar puncture under isoflurane anesthesia in Li-heparin coated sample tubes were separated by centrifugation (5000xg, 10 Min, 8°C). Plasma aliquots (200µl) were used for clinical chemistry analysis using a Beckman Coulter AU 480 autoanalyzer and adapted reagents from Beckman Coulter (Krefeld, Germany). A broad set of parameters were measured including various enzyme activities, as well as plasma concentrations of specific substrates and electrolytes in ad libitum fed mice, as described previously.^24^

For the mandible analysis, data sets of the skulls were acquired *post mortem* at 18 μm³ voxel resolution using a SkyScan 1176 in vivo micro-CT system (Bruker microCT, Kontich, Belgium). All scans were acquired with the following parameters: 80 kV source voltage, 313 μA source current, 300 ms exposure time, 0.5° rotation step, 2 frame averages, aluminum-copper filter. Image reconstruction was performed using NRecon (Bruker microCT), subsequent image analysis was carried out using GOM Inspect Suite software (GOM, Braunschweig, Germany) and 3D Slicer (version 4.10; www.slicer.org).

For pathological analyses (4 male, 2 female homozygous KO mice), mice were euthanized with CO2 and the visceral organs were analyzed macroscopically as described before.^25^ We used 4% formalin-fixed buffered paraffin-embedded (3 μm) sections of 28 organs/tissues including the kidney, stained with hematoxylin and eosin (H&E). Immunohistochemical staining of the kidney was carried out on 1-2 µm paraffin sections. The procedure was performed by an automated immunostainer (Leica Bio systems, Wetzlar, Germany; BOND RX, using the ds9800-DAB Polymer Detection System). Heat induced antigen retrieval with citrate buffer (pH 6) was performed prior to incubation with following primary antibodies: Rabbit polyclonal Anti-Aquaporin2 (Sigma-Aldrich, St. Louis, MO, USA; Nr. AB3274, dilution 1:600) and rabbit monoclonal Anti-Cytokeratin 8, (CK8; Abcam, Cambridge, UK; Nr. ab53280, dilution 1:250). Target tissue stained without primary antibody was used as negative control. Two pathologists analyzed the slides independently.

***Image analysis of CK8-immunohistochemistry***

Tissue sections were scanned with NanoZoomer® 2.O HT (Hamamatsu Photonics, Hamamatsu, Japan) and subsequently analyzed with QuPath Version 0.2.3 software (downloaded from the QuPath homepage located at Github, https://QuPath.github.io/) ^29^. First, a new project was created and image type was set to the used staining/immunohistochemistry (H-DAB). Second, the annotation tool was used to mark positive and negative cells (nucleus and cytoplasm) in different areas and staining intensities in order to train the cell detection algorithm. Third, mean cell size was calculated (10 ± 3 µm) after precise measuring at representative sections. Fourth, by using the Polygon tool, cortex region was outlined manually by a pathologist for every single renal tissue section. Fifth, for each outlined cortex region, the positive cell detection tool was executed after checking nucleus, cytoplasm and intensity parameters as well as adjusting the intensity threshold parameters for the cytoplasmatic staining (CK8). During this process, QuPath was using a cell segmentation and classification algorithm. Each slide in the project was checked for incorrect cell classification manually. Sixth, detection and annotation measurements were extracted after computing and transferred to Excel (Microsoft, Redmond, WA, USA) for further statistical analysis. To confirm the accuracy of QuPath, a comparison with manual counting was performed. Both positively and negatively CK8-stained cortical cells were quantified and the proportion of positive to total cells was calculated.

***Statistical analysis (mouse experiments)***

Depending on the type and the assumed distribution of data, Fisher’s exact test, Mann-Whitney test or 2-way ANOVA were used to analyze the data as indicated in the figure legends. A p-value ≤0.05 was used as level of significance. The GMC data set was not corrected for multiple testing.

***Cell culture and CRISPR/Cas9 mutant generation (Foxd2 deficient metanephric mesenchyme cell models*)**

The immortalized mouse metanephric mesenchyme mk4 cell line was a kind gift of Steven S. Potter, Division of Developmental Biology, Children's Hospital Medical Center, Cincinnati, OH, USA. Cells were cultured in DMEM medium (Sigma-Aldrich) with 10% FBS under standard conditions. CRISPR/Cas9 gene targeting was performed as previously described.^30^ In brief, short guide RNAs (sgRNAs) were designed using different designing tools including the Benchling (https://www.benchling.com/crispr/) and the CRISPR Design (https://zlab.bio/guide-design-resources, recently shut down by the Zhang lab), targeting the corresponding mouse *Foxd2* location of the index individual’s frameshift variant. Binding to other sites in the genome was excluded. A sgRNA targeting a sequence not present in the mouse genome was used as control sgRNA. The two sgRNA sequences used are sgRNA-*FoxD2*: GTAGCCGTAAGCGCCGTAAG, TTCGCCGCTTACGGCGCTTA and sgRNA-neg-ctrl: 5′-GCGAGACAGTTTGACCGTCT-3′. SgRNAs were subcloned into the pSpCas9 (BB)-2A-GFP (PX458) vector and correct insertion of the sgRNAs was verified by Sanger sequencing (data not shown). *Foxd2* sgRNAs were tested for gene editing efficiency using a T7 endonuclease assay. In brief, a mixture of 7 µl of the purified PCR product of the genomic region of interest, 2 µl NEB 10 x buffer 2, and 10 µl mQ was subjected to a thermal cycling as described in the protocol of T7 endonuclease kit (New England Biolabs). The mixture was subsequently incubated with 1 µl T7 endonuclease at 37C for 1h, and run on 2 % agarose gel for examining a presence of mutated alleles and the editing efficiency. SgRNA binding to other genomic regions was excluded by genome-wide BLAST analysis. A sgRNA not targeting any genomic region of the mouse was used to generate control cell lines.

mk4 cells were transfected with the px458-*Foxd2* or the px458-neg-ctrl plasmid using Lipofectamine2000 (Invitrogen, Waltham, MA, USA) according to the manufacturer’s protocol. Green fluorescent protein (GFP) expression was checked after 24 h, 48 h, and 72 h post-transfection. At 48 h post-transfection, cells were harvested and sorted for GFP signal using FACS single-cell sorting. GFP-positive cells were collected in 96-well plates (Corning, Corning, NY, USA) and cultured in a standard condition. For clones that expanded enough, genomic DNA was extracted using DNeasy Blood & Tissue Kits (Qiagen, Hilden, Germany). gDNA was subjected to PCR amplification of the region around the targeted sequence and Sanger sequencing afterward. Clones with homozygous variants changing the reading frame were chosen for subsequent transcriptomics analysis and qPCR verification.

***RNA sequencing***

RNA preparation, library preparation and RNA sequencing were performed as previously described.^31^ RNA was extracted using the Nucleospin RNA purification Kit (Macherey-Nagel, Düren, Germany) according to the manufacturers protocol. In brief, cells were lysed in proprietary buffer RLT containing β-mercaptoethanol. Lysis mix was transferred into a spin column and subsequently washed and treated with DNAse to remove genomic DNA contamination. The quality and quantity of total RNA were measured using the High Sensitivity RNA Screen Tape Analysis (Agilent) and Qubit RNA HS Assay Kit (Thermo Fisher Scientific). 500 ng of total RNA was used for RNA library preparation using the KAPA RNA HyperPrep with RiboErase (Roche, Basel, Switzerland) and KAPA SI Adapter kit set A+B (30uM; Roche) according to the manufacturer’s protocol with specific settings for the fragmentation step (6.5 min at 94^o^C) and the amplification of the library (7 cycles). The final libraries were quantified using the Qubit DNA HS Assay Kit (Thermo Fischer Scientific) and evaluated for quality using the High Sensitivity DNA Screen Tape Analysis (Agilent). An Illumina (San Diego, CA, USA) NextSeq 500 sequencer with a 2 × 42 bp paired-end module was used for RNA sequencing and approximately 20 Mio reads obtained per sample. Bioinformatics analysis was performed using the GALAXY platform (https://usegalaxy.org/).

***RNA seq bioinformatics***

RNA seq analysis was performed as previously described.^32^ In brief, sequence reads were mapped to the mouse reference genome GRCm38/mm10 (iGenomes, Illumina; chromosomes 1-19, X, Y, M) using the Rsubread v1.28.1 package in R v3.4.4. Gene counts were retrieved from the mapping, applying the Rsubread::featureCounts function with the iGenomes reference genome gtf file for annotation (archive-2015-07-17-32-40 and archive-2015-07-17-33-26 for GRCM38). For differential expression analysis, DESeq2 v1.18.1 was used. First, the counts were normalized by library size (DESeq2::estimateSizeFactors) and by gene-wise dispersion (gene-wise geometric mean over the samples; DESeq2::estimateDispersions), then, the differential expression analysis was performed applying negative binomial generalized linear model fitting and Wald statistics on the normalized count data (DESeq2::DESeq2). Subsequently, the results were filtered for adjust *p* < 0.05 and the indicated log2 fold change values (log2FC) in the figure legends.

***Heatmaps***

For heatmaps, the normalized counts were gene-wise-scaled to cluster the counts by their expression tendency between the samples (stats::kmeans function). In addition, the counts were centered (gene-wise mean set to zero) which explains why some count values are negative (below gene-wise mean). The result was plotted using the pheatmap::pheatmap function (pheatmap v1.0.10) with deactivated function-intrinsic clustering.***GO enrichment analysis***

GO enrichment analysis was performed as previously described.^32^ In brief, gene names were converted into Entrez IDs (biomaRt v2.34.2). Gene group enrichment was retrieved using the clusterProfiler::enrichGO function (clusterProfiler v3.6.0 with org.Mm.eg.db v3.5.0). Pathway enrichments were retrieved using the clusterProfiler::enrichKEGG function (KEGG.db v3.2.3) or the clusterProfiler::enricher function (gskb v1.10.0). For all analyses, a q-value cut-off of 0.05 (pAdjustMethod Benjamini-Hochberg) was applied.

***Volcano plot***

The volcano plot was plotted using the normalized count data with the log2FC and negative log10 of the p-values obtained from the DESeq2 analysis by applying the Glimma::glXYPlot function (Glimma v1.6.0).

***PAX2 immunofluorescence***

mk4 mutant and control cells were cultured in glass bottom chamber slides for immunofluorescence analysis. In brief, cells were washed with PBS and fixed five minutes in 5 % PFA. After three washes, cells were permeabilized using 0.1 % Triton for 3 minutes, washed three times and the incubated in 5 % BSA for one hour at RT to bock unspecific reactions. Incubation with Pax2 antibody (ab79389, Abcam, Cambridge, UK) was then performed for one hour (1:200 in 5 % BSA) and after five washes, cells were incubated in corresponding fluorescence tagged secondary 1:1000 antibody dilution for 30 minutes, washed five times in PBS and then mounted in vectashield with DAPI. Images were taken with a Zeiss confocal microscope and processed with AxioVision 4.8 and Fiji software.

***Western Blot analysis***

Gel electrophoresis and western blotting was performed as previously described.^33^ In brief, cells were grown under standard conditions and harvested using trypsinisation. Cells were subsequently washed with PBS, cells pelleted and lysated con ice using lysis buffer (50 mM Tris-HCl [pH 7.5], 150 mM NaCl, 1 % NP-40) supplemented with complete protease inhibitor cocktail (Roche) followed by addition of 4 x NuPAGE sample buffer heating samples for 10 min at 70°C. Subsequently, samples were run on NuPAGE Novex 4 %-12 % Bis-Tris SDS-PAGE gels and blotting performed overnight at 4 degrees, Membranes were then incubated with rabbit anti-PAX2 antibody (ab150391, EPR8586, Abcam) for 2 hours, washed 3 times in PBS and subsequently incubated with secondary anti-rabbit antibody, washed 3 times and finally developed using ECL.

**Results**

***Supplementary Case Report Family 1***

The index individual (VII-3) is the second son of consanguineous healthy parents (1^st^ degree cousins) of Arabic origin. He has a healthy older brother and two healthy younger sisters. Pregnancy was uneventful but already in the neonatal period, bilateral hypoplastic kidneys were identified by ultrasound. Furthermore, he had dysmorphic facial features (mandibular retrognathia, micrognathia, severe dental abnormalities, a high-arched palate, hypertelorism, exophthalmos, down-slanting palpebral fissures, flat nasal bridge, low-set dysplastic ears). Documented kidney lengths were 5.7 cm (right and left) at four years of age (<1P), 5.8 cm (right) and 6.4 cm (left) at age five years (<1P) and 5.6 cm (right) and 5.5 cm (left) at 7 years of age (<1P) (Figure 1D). Both kidneys showed increased parenchymal echogenicity and loss of corticomedullary differentiation. There were no other urinary anomalies reported and he had normal male genitalia. Chronic kidney disease (CKD) evolved from stage II at four years (estimated glomerular filtration rate [eGFR] 86 mL/min/1.73m^2^ [Schwartz formula]) to stage III at five years (eGFR 59 mL/min/1.73m^2^) to stage IV at seven years of age (eGFR 26 mL/min/1.73m^2^). Proteinuria was first identified at five years of age (1.5 g/24h) and angiotensin converting enzyme (ACE) inhibition was started. At seven years of age, proteinuria was 2.7 g/24h without peripheral edema. Mental retardation was present (he attended a special needs school) but no formal intelligence testing had been undertaken. Due to micrognathia, the index individual was severely dysarthric and had delayed speech development. At the last clinical examination at nearly eight years of age (7 10/12), he had a height of 121 cm (9P), a weight of 22 kg (11P), and a head circumference of 51.5 cm (15P). Blood pressure at that time was hypertensive at 119/72 mmHg (>95P for systolic blood pressure). Furthermore, glucose-6-phosphate dehydrogenase deficiency (G6PD) was diagnosed by neonatal screening. Allogenic kidney transplantation was performed at 11 years of age. The clinical tentative diagnosis for the syndromic aspects including developmental delay and hypoplastic kidneys was Opitz GBBB syndrome (MIM 145410) and ES was done to obtain a genetic diagnosis at 7 11/12 years of age.

Three relatives of the index case, all first cousins once removed, were likewise affected by syndromic CAKUT; individuals V-13, V-14 and V-19 (see Figure 1A for a detailed pedigree of the family). V-19, a now 21 years old female, also had developmental delay (no active speech, she attended a special needs school) and facial dysmorphy (micrognathia, discrete retrognathia). Further, she had an imperforate anus at birth and was diagnosed with kidney hypoplasia. At six years of age, she had CKD stage V with an eGFR of 11 mL/min/1.73m2 and proteinuria of 5 g/24h. Kidney biopsy at five years of age showed global and focal glomerulosclerosis but was of limited value as only five glomeruli were sampled. She received an allogenic kidney transplant at seven years of age. The clinical tentative diagnosis in this affected female relative also was Opitz GBBB syndrome. V-14 is an older brother of V-19 and was kidney transplanted twice, at six years of age and 25 years of age. He had aposthia, a horseshoe kidney and hypospadias. He also showed facial dysmorphism (micrognathia, crowded teeth) and attended a special needs school. A third cousin once removed, V-13, the oldest brother of V-19, died at the age of six years after an admission to the hospital due to fever and anemia. He had a phenotype featuring aposthia, developmental delay and facial dysmorphy (micrognathia). He had hypospadias as a CAKUT manifestation, but it is unknown if there was a kidney malformation or CKD in this individual.

***Supplementary Case Report Family 2***

Individual-II1 was referred to the pediatric nephrology clinic to maintain her chronic hemodialysis (HD) program after rejection of transplanted kidney. She was born 2,620 grams at 40th week of gestational age from first cousin healthy parents. She was diagnosed with end-stage kidney failure at the age of six years, incidentally. The etiology of CKD was unknown. Initial ultrasonography showed bilateral hypoplastic kidneys with increased echogenicity without any other obstructive findings. Voiding cystourethrography showed no vesicoureteral reflux. She received a preemptive kidney transplantation from her father at the age of six years. Almost one year after transplantation, her serum creatinine levels reached 1.45 mg/dL (estimated glomerular filtration rate [eGFR] 32.8 ml/min/1.73m^2^, according to the modified Schwartz formula ^34^) and renal biopsy showed calcineurin inhibitor toxicity. At the age of 9 years, serum creatinine reached 3.5 mg/dL. A second renal biopsy was performed, which showed a chronic active T-cell-mediated rejection that was treated with metylprednisolone and anti-thymocyte globulin (ATG). However, eGFR was below 10 ml/min/1.73m^2^ and hemodialysis was started. On admission at the age of 9 years, she was anuric; her weight (19 kg, -2.54 SD) and height (117 cm, -2.61 SD) were below the third percentile. Blood pressure was 132/92 mmHg (above 95th percentile for age, gender and height percentile). Height-age adjusted body mass index (BMI) SD was -1.15. Short stature (height -2.95 SD), hypertension (blood pressure 170/100 mmHg) and dysmorphic features including down-slanting palpebral fissures, deeply set eyes, laterally extended eyebrows, micro-retrognathia, mild ptosis on left eye, high palate, dental crowding, fusiform fingers, sandal gap in both sides, and central obesity were noted in physical examination (Figure 1E, F and Table 1). Echocardiographic examination revealed mild pericardial effusion and increased left ventricular mass index (52.7 g/m^2^) while ejection fraction was normal. Eye examination showed left esotropia and bilateral posterior subcapsular cataract that were identified at the age of six years. In her history, neurodevelopmental delay was also noted. Namely, she had started to walk at the age of two years and begun to speak with few words at the age of three years. She was diagnosed with a special learning disability in the 1st year of elementary school and had learned to read and to write in the third year of elementary school. Cranial magnetic resonance imaging performed at the age of 13 years showed enlarged ventricles and enlarged subarachnoid space. Microarray analysis for CNVs was found to be normal. During her follow-up, she had a second living donor transplantation and is still followed-up with a functioning graft (serum creatinine 1.2 mg/dL). Clinical and laboratory findings are given in Table 1.

Individual II-2 was referred to pediatric nephrology due to persistent proteinuria at the age of seven years. Her height was 117 cm (-2.61 SD). She had no edema and systemic examination was normal except for dysmorphic findings similar to the affected sibling including down-slanting palpebral fissures, laterally extended eyebrows, micro-retrognathia, left deviation of nasal axis, tapering of distal phalanges of fingers, sandal gap in both sides, short toes, central obesity, micro-retrognathia, teeth anomalies and neurodevelopmental delay (she had started to walk at 20 months of age and had her first words at 30 months of age). She had not done well with maths and science when compared to her peers (Figure 1G, H, Table 1). Serum creatinine level was normal for the age (i.e., 0.35 mg/dL). Renal ultrasound showed bilateral increased renal parenchymal echogenicity and left kidney hypoplasia. Voiding cystourethrography (VCUG) showed no vesicoureteral reflux (VUR) but increased bladder wall thickness. Tc-99m dimercapto succinic acid radyonuclid scanning showed hypoplasia of the left kidney without findings of dysplasia. Differential functions of the left and the right kidneys were 40% and 60%, respectively. A renal biopsy was compatible with focal segmental glomerulosclerosis (Figure 1I). ACE inhibitor was started for proteinuria.

On her current examination at the age of 17 years, her weight and height were as follows: 55 kg (-0.33 SD); 152 cm (-1.83 SD). Height-age adjusted BMI SD was 1.46 (BMI 23.8kg/m²). Blood pressure was normal (90/62 mmHg). Laboratory findings showed impaired renal function (CKD stage 3 with an eGFR of 38 mL/min/1.73 m^2^ based on the modified Schwartz formula). She is still treated with enapril 10 mg per day and has mild albuminuria (58 mg/day). Microarray analysis for CNVs was found to be normal in this sibling as well.

***Supplementary Case Report Family 3***

The index individual was the fifth child out of six born to parents of Bedouin descent who are first cousins. The mother was diagnosed with anti-phospholipid syndrome and immune thrombocytopenia, and was noted to have an extrarenal pelvis. During pregnancy, two anatomy scans were reported as normal but, at 35 weeks of gestation, small kidneys were noted. On the third day of life, she had a high creatinine level of 1.7 mg/dl which gradually decreased. She was discharged home with a creatinine of 0.9 mg/dl at the age of one week, and over the next two years her creatinine levels stabilized at around 0.45 mg/dl. Around age two years, she first presented with nephrotic syndrome, which did not respond to steroid treatment. Renal ultrasound showed bilateral dysplastic kidneys; the right kidney had poor corticomedullary differentiation, the left kidney was noted to be in the left central abdomen with small hypoechogenic findings suspicious for cysts and possibly dilated renal pelvis. Renal biopsy was planned but not done due to difficult access to the kidneys. She was treated with ACE inhibitors and angiotensin receptor blockers with fluctuations in the degree of proteinuria but levels always remained within the nephrotic range. Treatment with tacrolimus was also attempted but discontinued due to rapid increase in the patient’s creatinine levels and hyperkalemia. Her renal functions worsened over time and, at age five and a half years, she was started on peritoneal dialysis. At age seven years, she underwent allogeneic renal transplantation.

Medical history is also significant for mild developmental delay – she walked at one year eight months and had mild speech delay, but currently attends a regular school. She had esotropia and hypermetropia causing amblyopia, and she wears glasses. She had a single episode of febrile seizure. She follows up with cardiology due to left ventricular dysfunction attributed to hypertension, which improved with treatment. Trivial pulmonic stenosis was noted on an early echocardiogram but no treatment was required. Until age three years, her height was at the 25^th^ percentile but was later noted to have short stature below the third percentile likely due to chronic renal failure. Growth hormone treatment was being considered.

On the last exam, she had microcephaly (2.8 standard deviations below mean), down-slanting palpebral fissures, ptosis, a slightly abnormal helix, mild retrognathia, and a small, prominent chin. She had a unilateral preauricular pit which was also seen in her mother and an unaffected brother.

**Figures and Tables**

***Supplementary Figure 1***

***
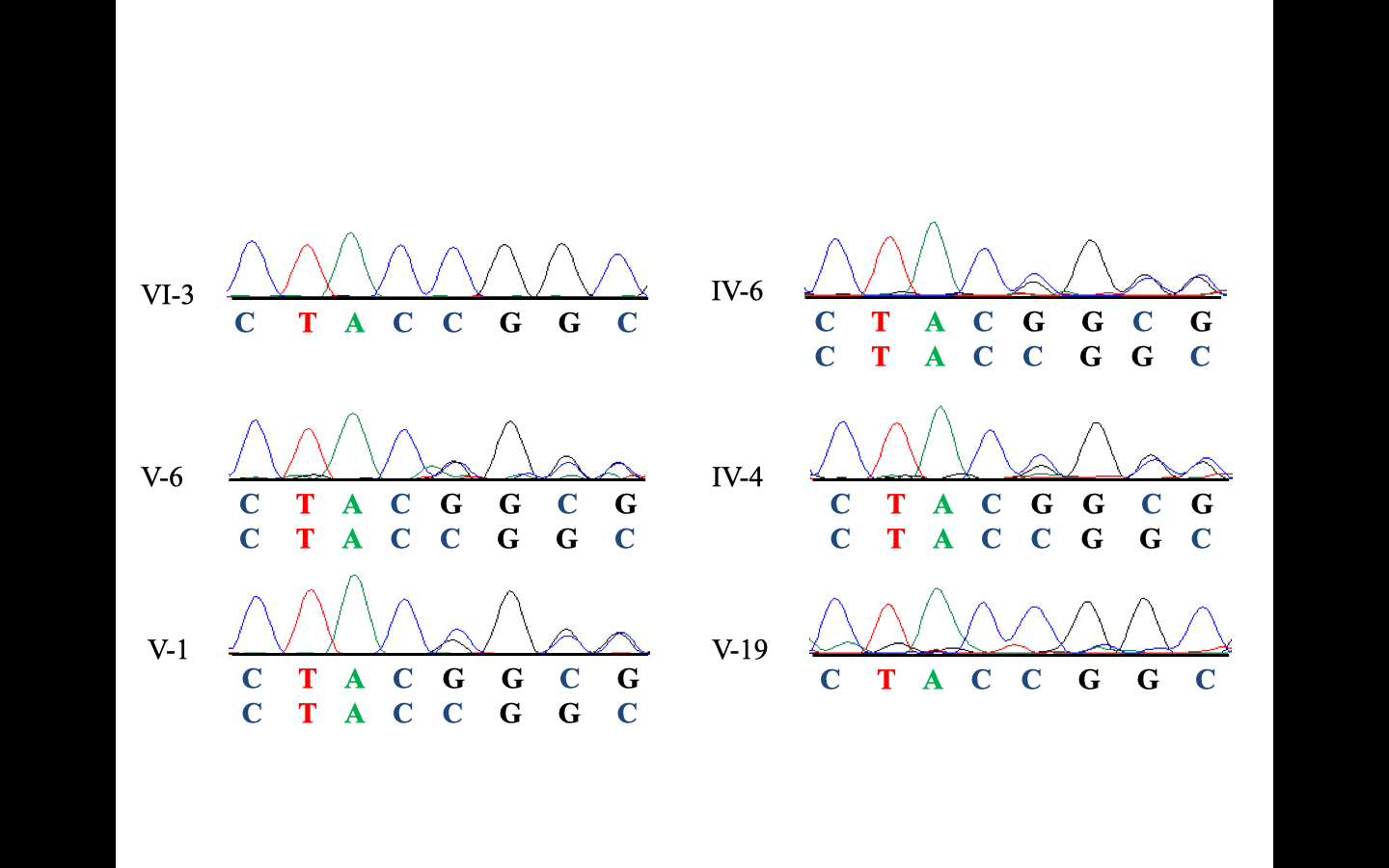
***

**Supplementary Figure 1.** **Chromatograms of the homozygous frameshift variant NM_004474.4:c.789dup, p.(Gly264Argfs*228) in *FOXD2* of family 1.** The wild type reading frame in the chromatogram is CTACGGCG. Affected individuals carried the variant homozygously (VI-3 and V-19), whereas the healthy parents/relatives were heterozygous for the variant (V-6, V-1, IV-6, IV-4).

***Supplementary Figure 2***

A

B

**

**

**Supplementary Figure 2.** **(A)** **Homozygosity mapping of family 2.** The overlapping homozygous stretches shared in two affected individuals (II-1 and II-2) are approximately 11.6 Mb in size between genomic positions of 47.600.000 and 59.200.000 on chromosome 1 (box). *FOXD2* resides here. **(B) Chromatograms of the missense variant NM_004474.4:c.628 A>G), p.(Met210Val) observed in *FOXD2*.** Affected individuals carried the variant homozygously, whereas the healthy parents were heterozygous for the variant. The unaffected sibling had a wild-type sequence.

***Supplementary Figure 3***

**A**


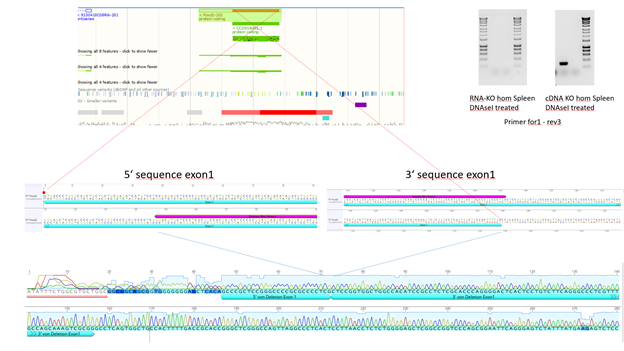


**B**

**
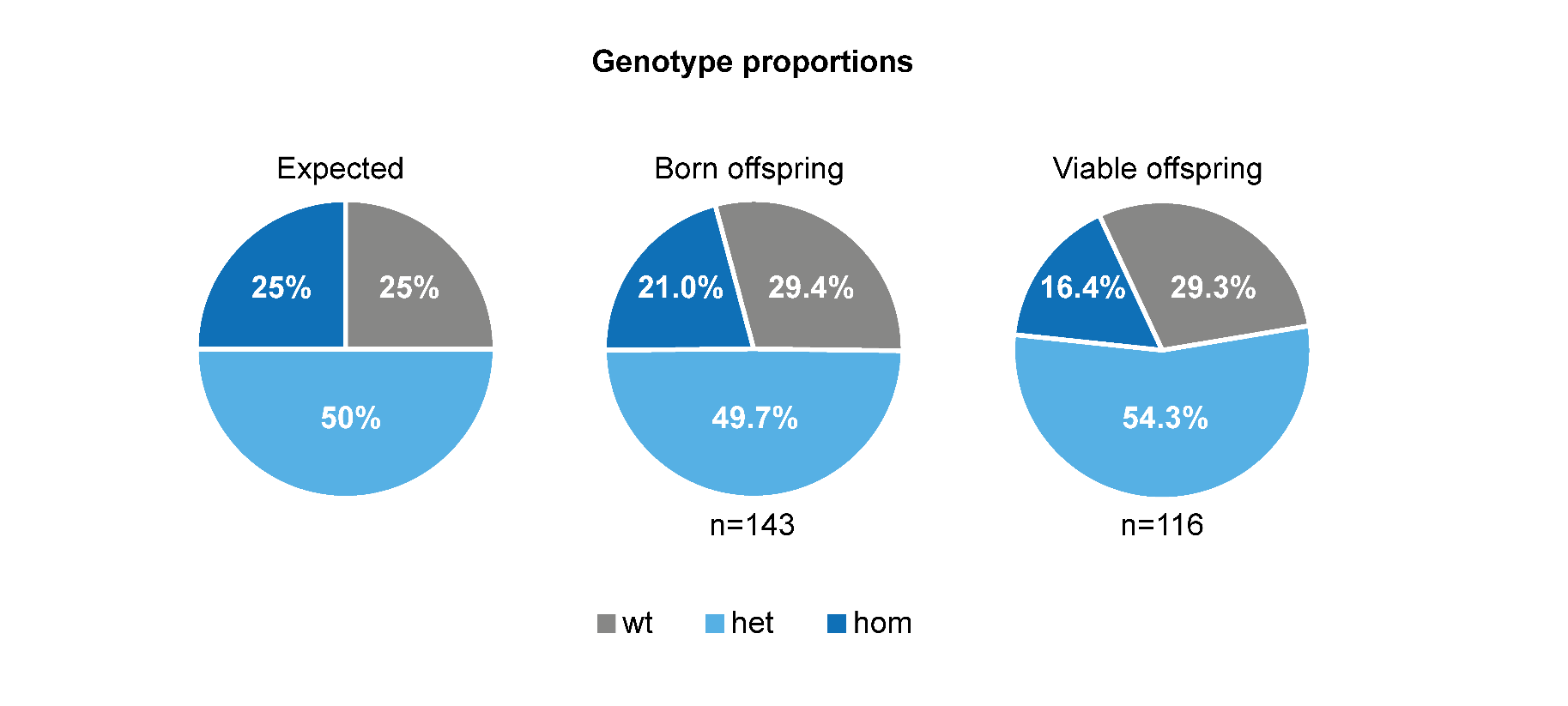
**

**Supplementary Figure 3.** (**A**) RT-PCR and cDNA synthesis confirms absence of *Foxd2* transcript in homozygous knockout (KO) animals. (**B**) Decreased viability in homozygous *Foxd2* KO newborns. Pie charts representing different genotype proportions. The “expected” genotype percentages are those anticipated according to a Mendelian distribution after double heterozygous matings. The “born offspring” genotype proportions show genotype proportions determined in pups born from such matings, and the “viable offspring” the genotype percentages within offspring that survived weaning. Percentages were calculated by dividing observed number of pups with the respective genotype by the total number of offspring (n = 143 born, n = 116 weaned) that was genotyped *100. het, heterozygous; hom, homozygous; wt, wild type.

***Supplementary Figure 4***

**
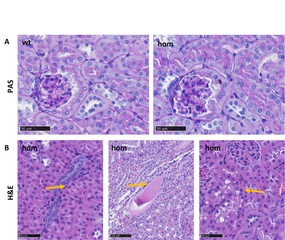

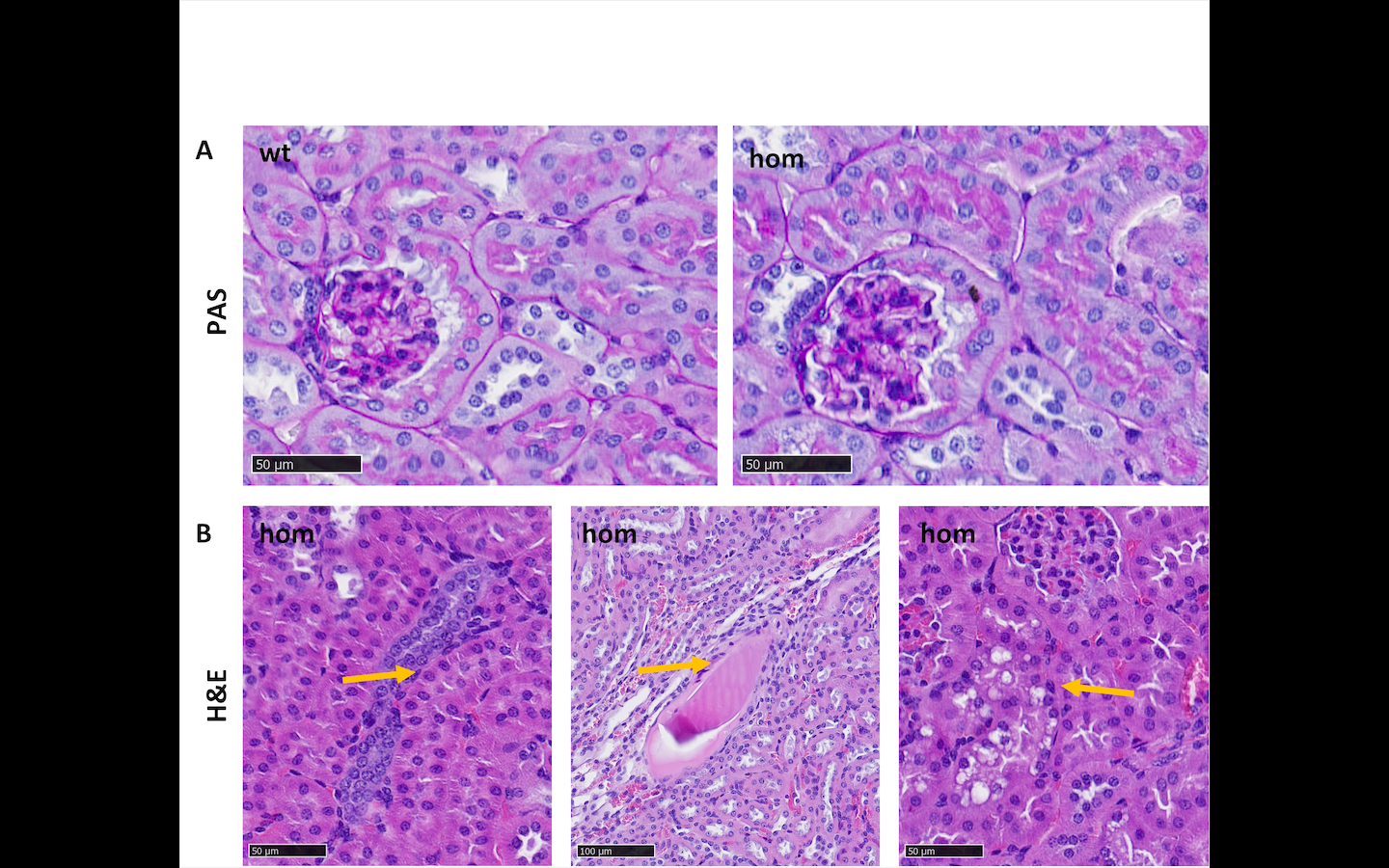
**

**Supplementary Figure 4. Other histopathological renal alterations in 16-week-old mice**. **(A)** Representative images of periodic acid-Schiff (PAS) staining of a wild-type (wt) mouse and a *Foxd2* homozygous knockout (KO) mouse. No alterations such as thickening of the glomerular and tubular basement membrane or increased proliferation of mesangial cells or Bowman's capsule are observed. **(B)** Hematoxylin-eosin (H&E) staining shows mild and focal alterations found only in homozygous KO mice. From left to right, examples of tubular basophilia, hyaline intratubular cast and presence of clear vacuoles (arrows) in the renal tubular epithelium.


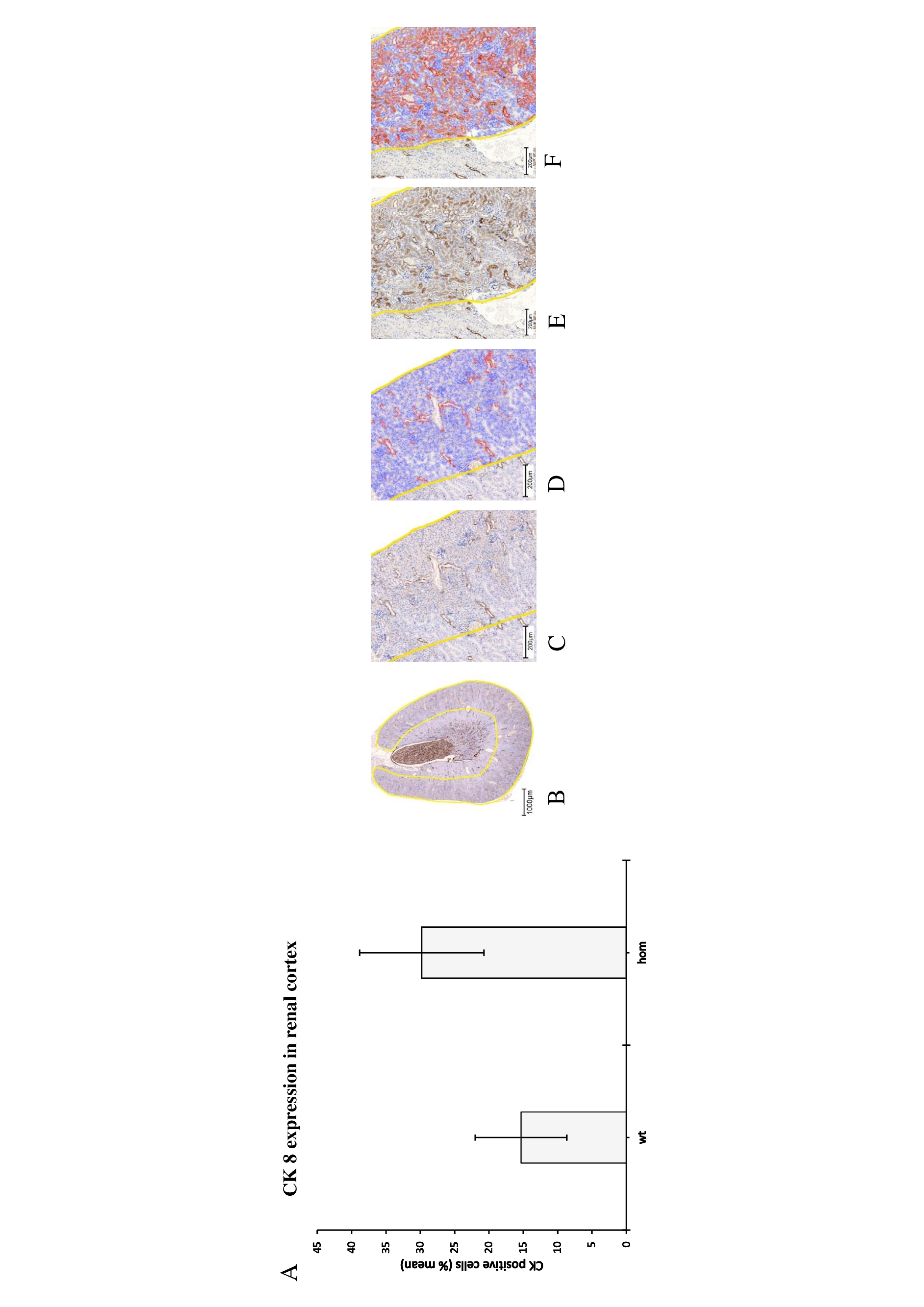
***Supplementary Figure 5***

**Supplementary Figure 5. *Foxd2* homozygous knockout (KO) increases cortical CK8 expression (A)*.*** *Foxd2* homozygous KO mice show highly increased CK8 expression. ****p* < 0.001 in two-sample t-test. Data are mean ± SD. **Representative examples of tissue image analysis CK8-staining of renal cortex.** Overview kidney with marked cortex **(B)**, section wild type pre-analysis **(C)** and post-analysis **(D)** and homozygous KO mice pre-analysis **(E)** and post-analysis **(F)**. Yellow = marked region, red = positive cells, blue = negative cells, brown = CK8 H-DAB immunohistochemistry.

***Supplementary Figure 6***

**
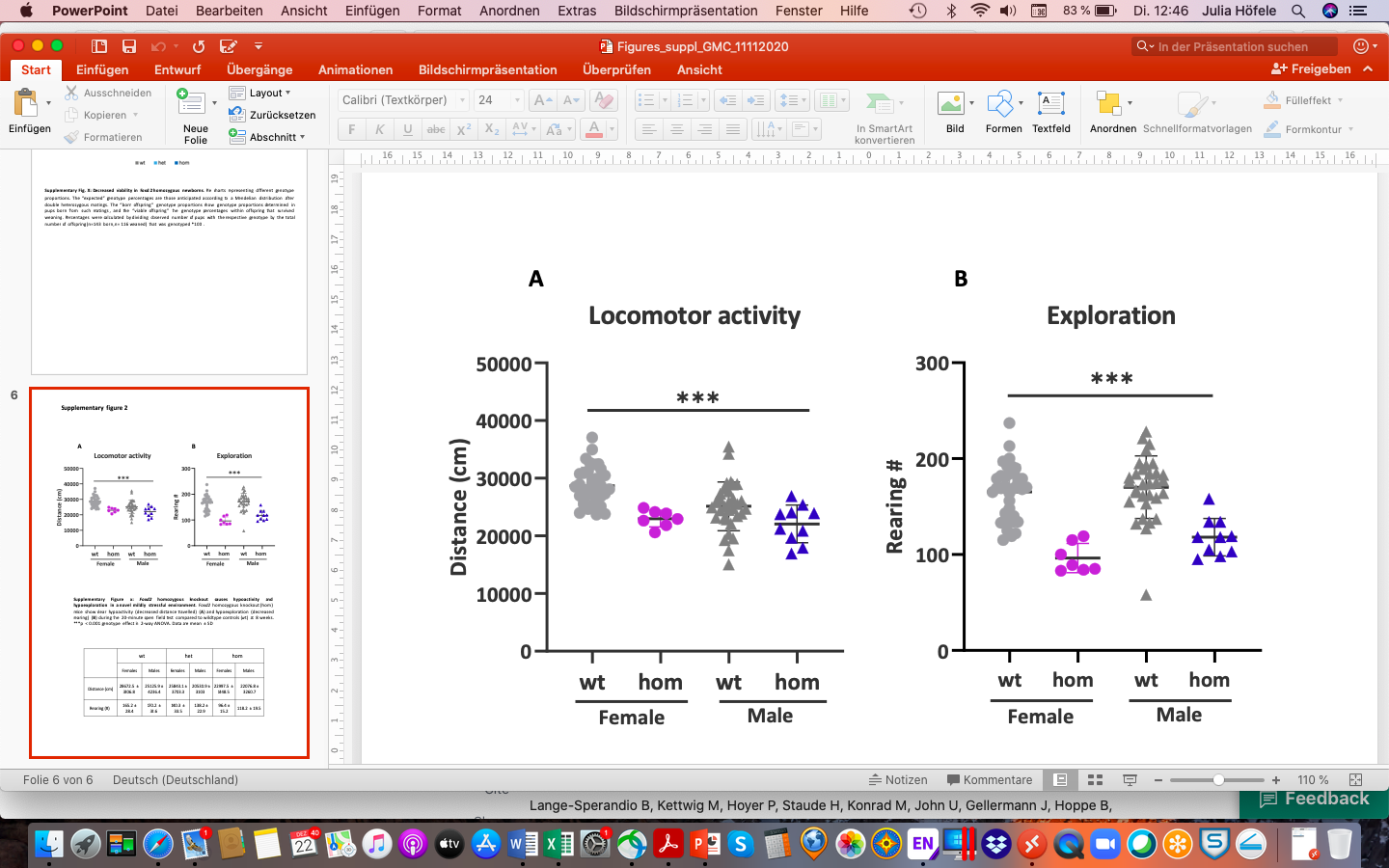
**

**Supplementary Figure 6. *Foxd2* homozygous knockout (KO) causes hypoactivity and hypoexploration in a novel mildly stressful environment*.*** *Foxd2* homozygous (hom) KO mice show clear hypoactivity (decreased distance traveled) **(A)** and hypoexploration (decreased rearing) **(B)** during the 20-minute open field test compared to wild type controls (wt) at 8 weeks. ****p* < 0.001 genotype effect in 2-way ANOVA. Data are mean ± SD.

***Supplementary Figure 7***

A


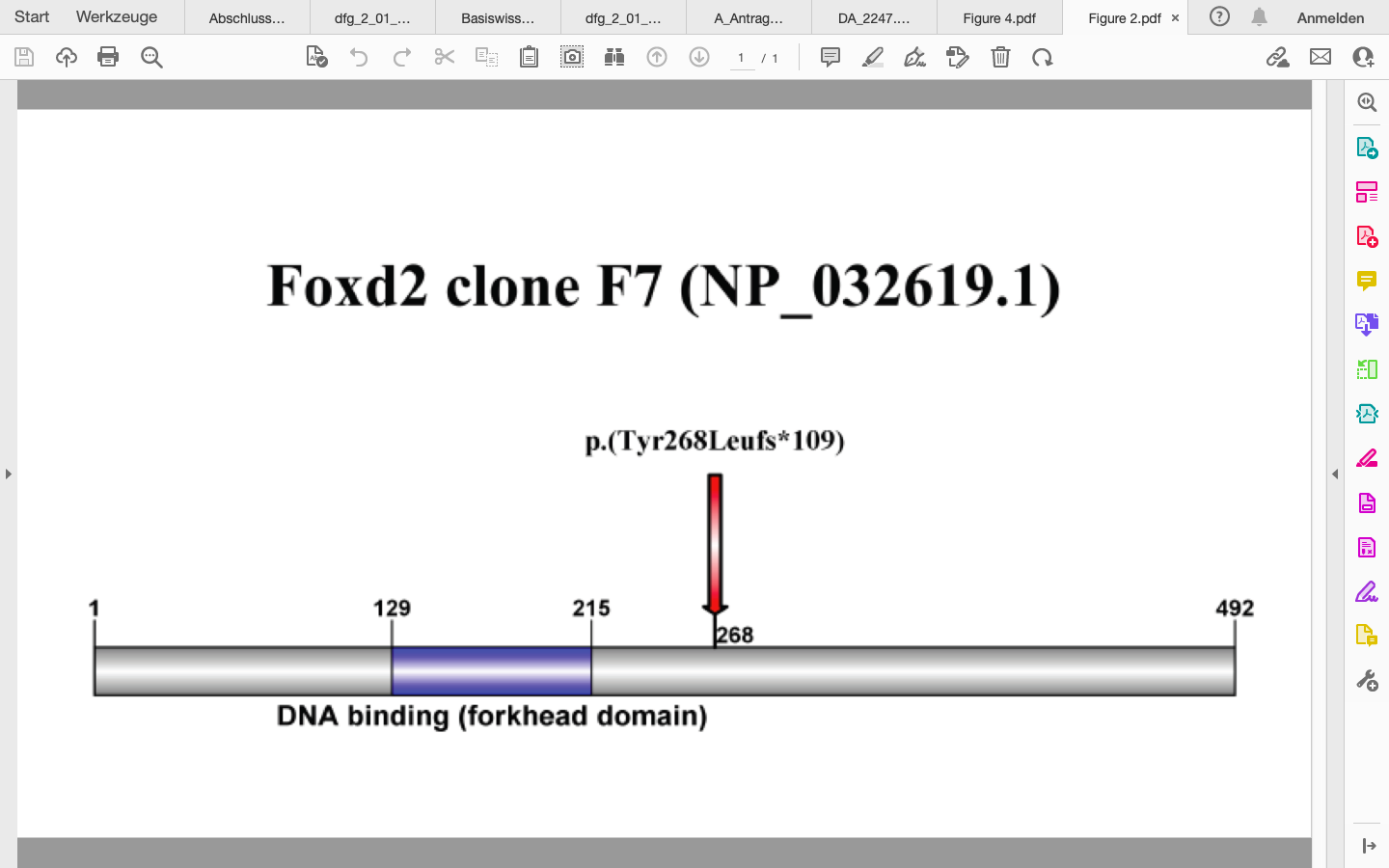


**223**


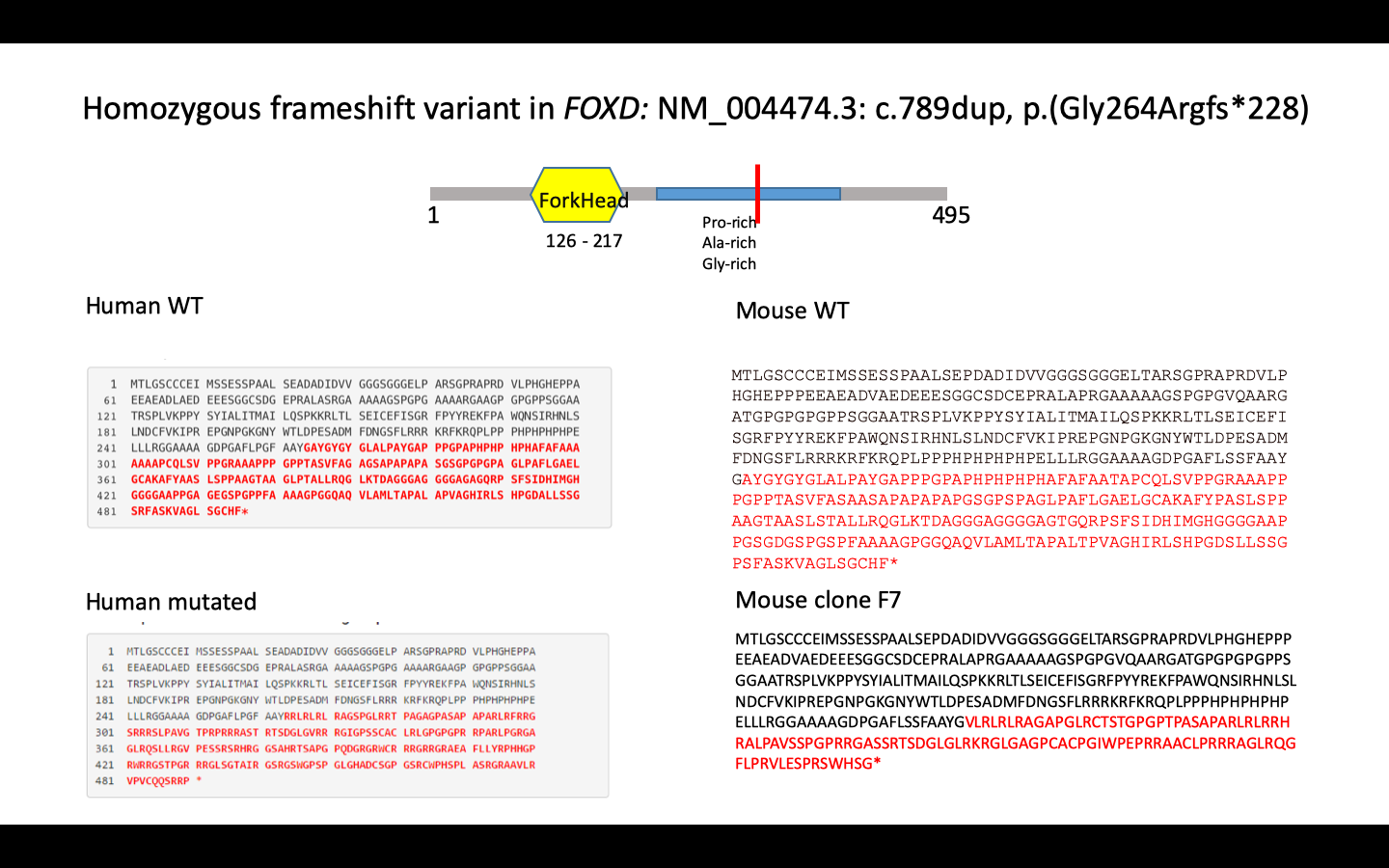


B

**Supplementary Figure 7. (A) Illustration of Foxd2 protein showing the Foxd2 mutated clone F7**. **(B) Amino acid sequence of human wild type FOXD2 (WT; NP_004465.3), human mutated FOXD2 p.(Gly264Argfs*228), mouse WT Foxd2 (NP_032619.1) and mouse Foxd2 clone F7.**

***Supplementary Figure 8***

**
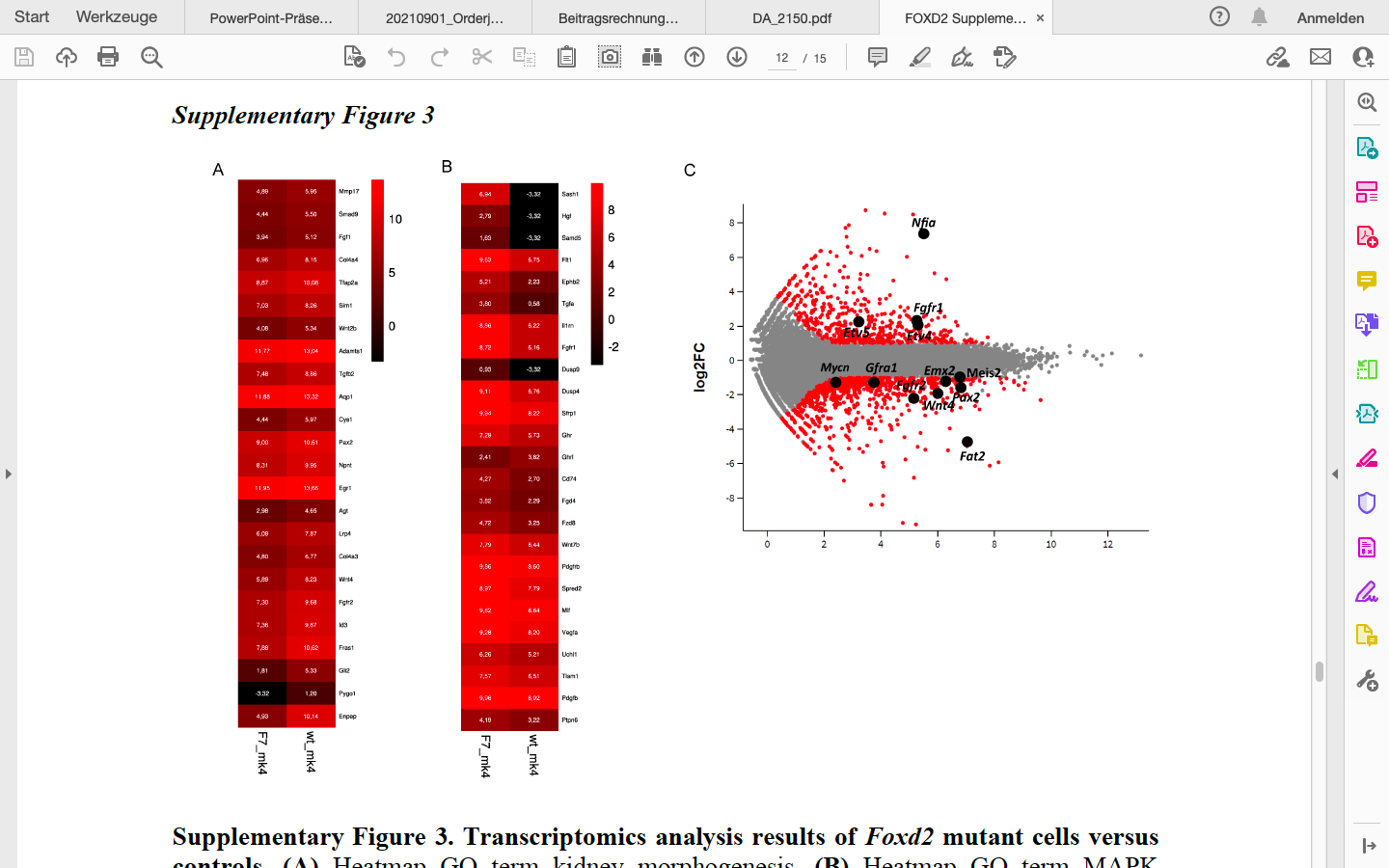
**
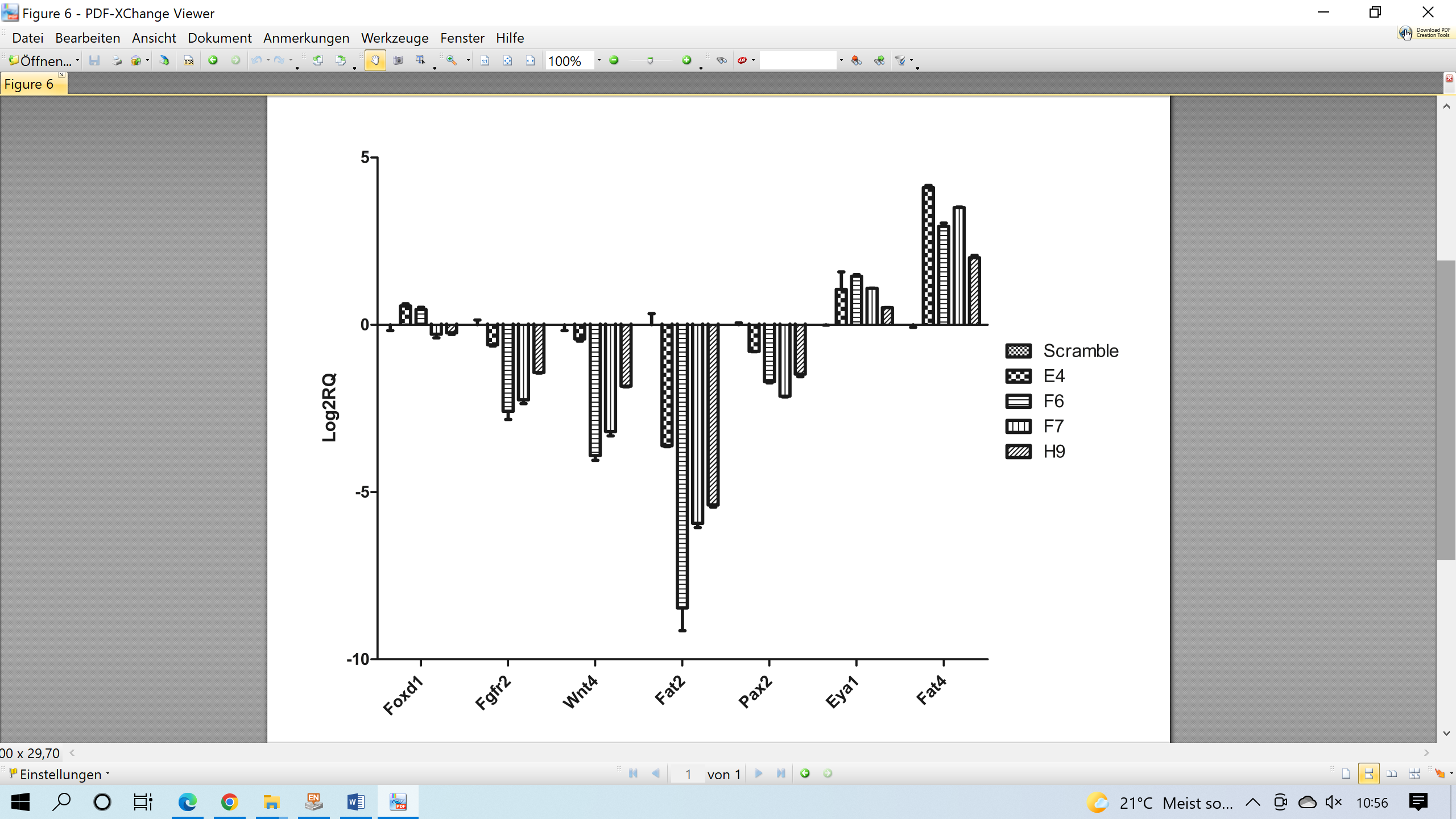


**Supplementary Figure 8. Transcriptome analysis results of *Foxd2* mutant cells versus controls. (A)** Heatmap GO term kidney morphogenesis. **(B)** Heatmap GO term MAPK signaling. **(C) qPCR.** Confirmation of selected genes of interest from transcriptomics in all four different clones versus control cells using real-time PCR analysis (y-axis, log 2 relative expression values [Log2RQ]). In agreement with RNA sequencing results, we could not detect differences in *Foxd1* gene expression, however confirmed downregulation of *Fgfr2*, *Wnt4*, *Fat2*, and *Pax2* and upregulation of *Eya1* and *Fat4* in *Foxd2* mutant compared to control cells. Data shown for n = 1 biological experiment. Error bars indicate standard deviation of technical duplicates.

***Supplementary Figure 9***

**A**

**
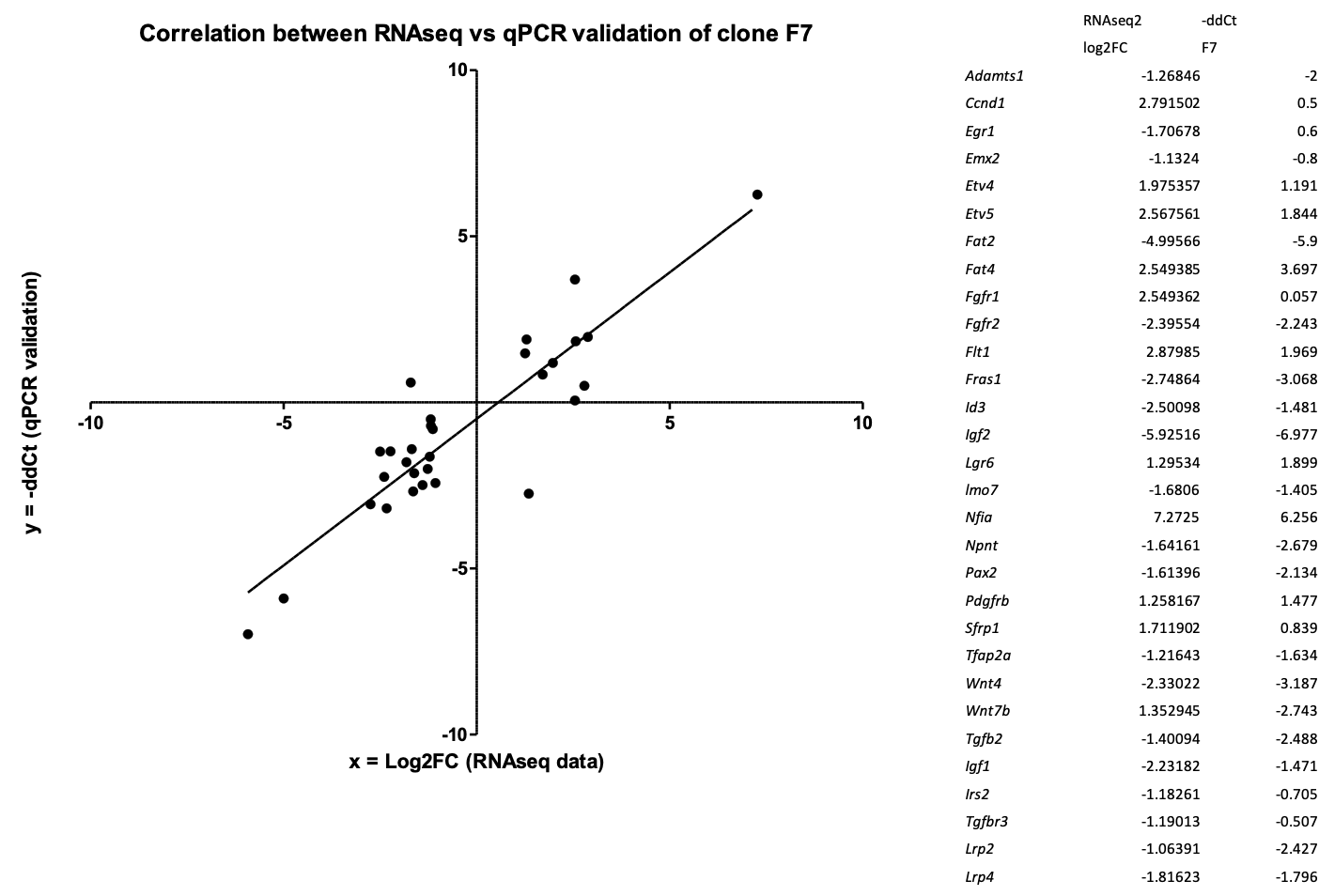
**

**B**

**
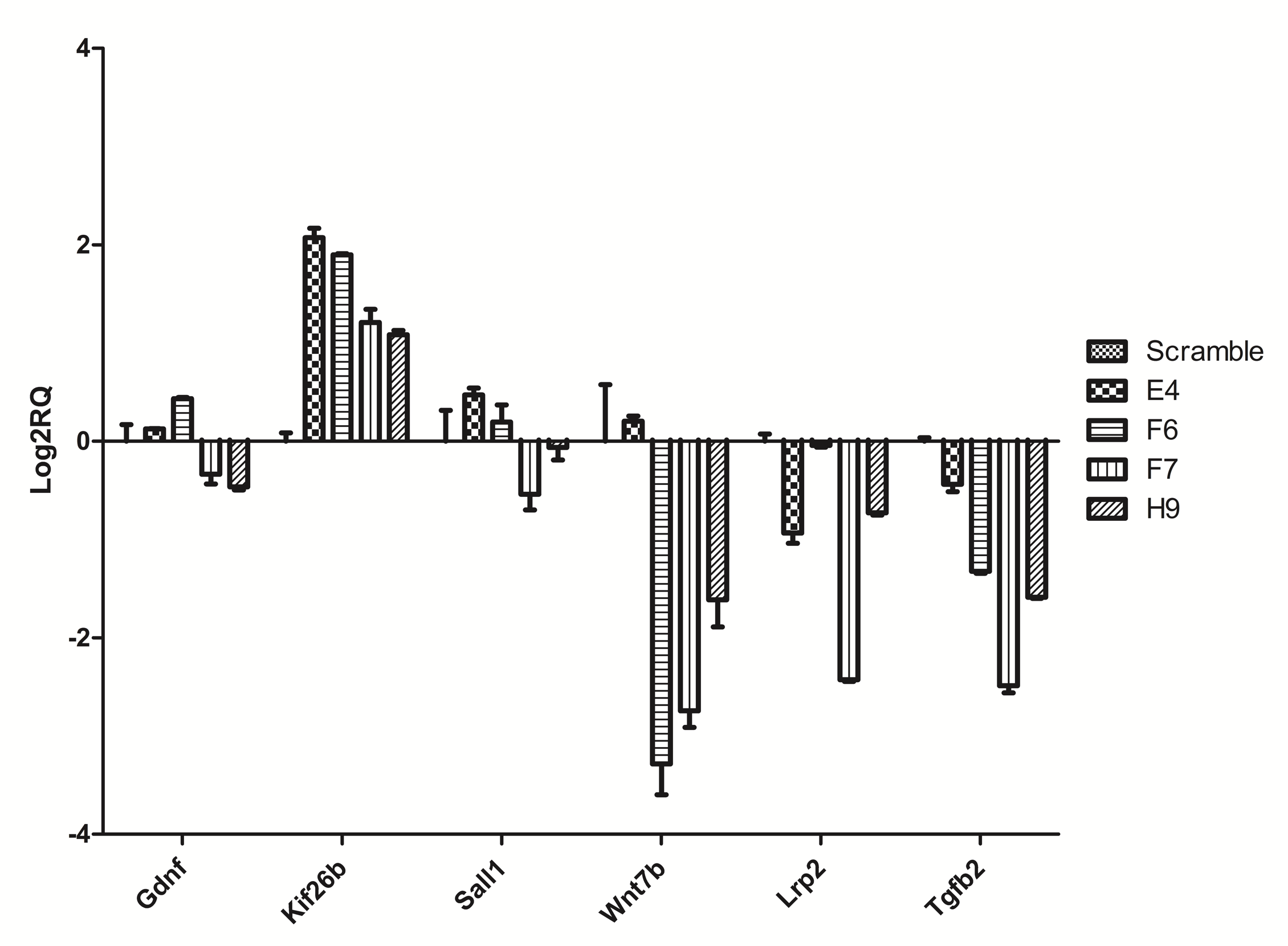
**

**Supplementary Figure 9. (A) Pearson correlation** of qPCR validated genes showing a highly significant correlation with the relative expression levels determined by RNAseq (Pearson R = 0.8955, *p* < 0.0001). **(B) qPCR validation** of selected genes of interest found to be differentially regulated by transcriptomics (y-axis, log 2 relative expression values [Log2RQ]; see also Figure 6 in the main text).

***Supplementary Figure 10***


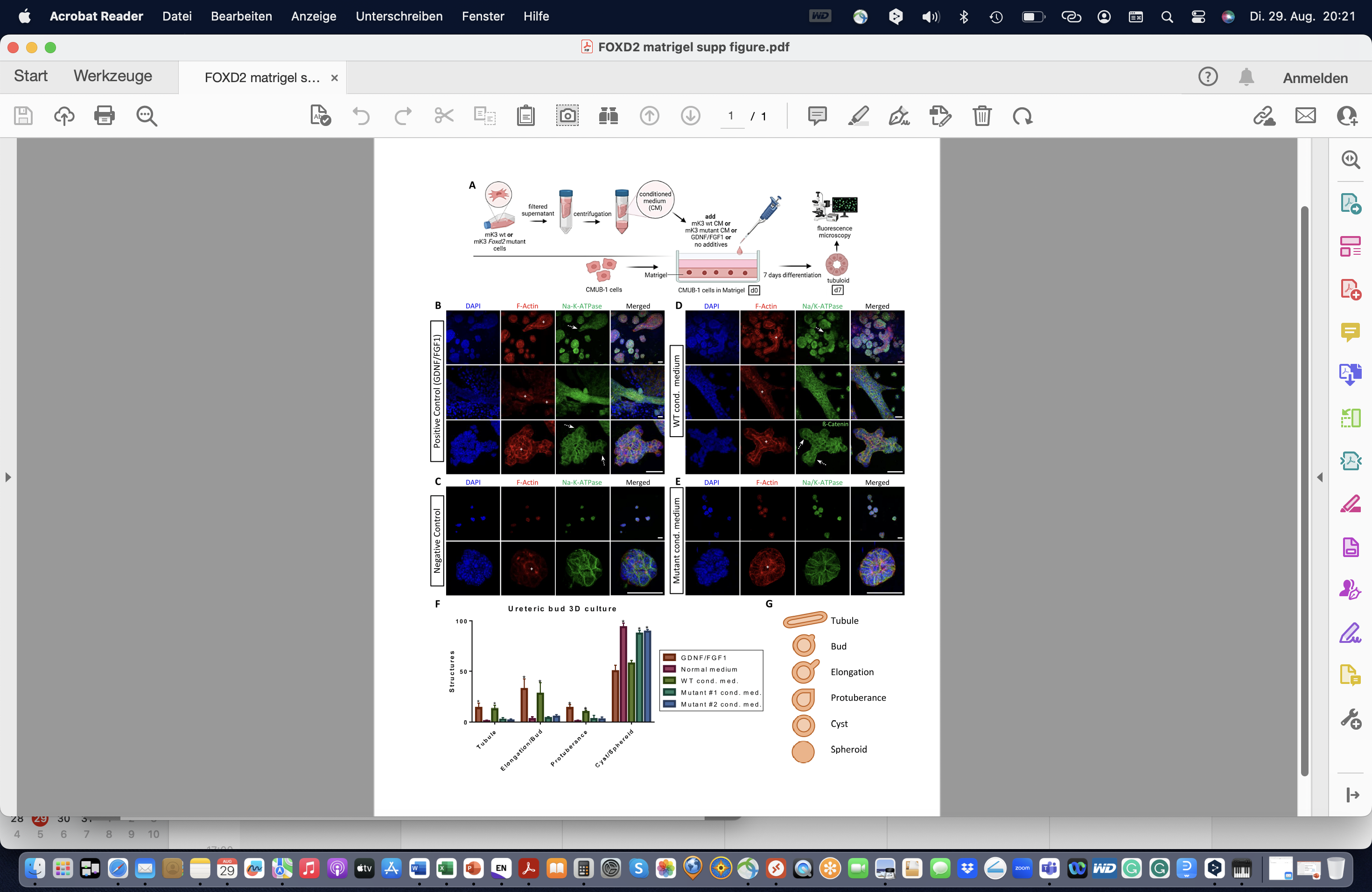


**Supplementary Figure 10. (A)** **Schematic overview of the CMUB-1 Matrigel culture.** Cell culture medium from mk3 *Foxd2* mutant or wildtype cells was concentrated by centrifugation and added to wildtype CMUB-1 cells in Matrigel on day 0 (“conditioned medium”). Normal (non-conditioned) medium containing additional GDNF and FGF1 served as positive control. Differentiation of CMUB-1 cells into 3D tubuloids was visualized by fluorescence microscopy. Graphics created in BioRender.com. (**B-E)** **Confocal images of 3D CMUB-1 cell culture** shows formation of different types of 3D structures after 7 days of differentiation. A graphical abstract of structure types is presented in (**G**). Immunofluorescence was performed using cell surface marker Na/K-ATPase or ß-Catenin (both in green), cytoskeleton marker F-Actin (red) and DAPI (blue) as nucleus marker. Scalebar: 30 µm. (**B) CMUB-1 cells** under addition of GDNF/FGF1 (positive control) (i) Confocal overview image displaying different ureteric bud tubuloid types. Arrow marks outgrowing tubule. Cross marks lumen. (ii) Tubule with lumen (cross). (iii) Arrows mark round cyst with budding (top) and elongation (right). **(C)** **Negative control**, CMUB-1 cells plus normal culture medium barely showed tubule formation (i) Overview image. (ii) Round cyst with lumen (cross). (**D)** **Addition of wildtype conditioned medium** resulted in tubule formation similar to what we observed upon addition of GDNF and FGF treated medium. (i) Overview image showing a tubule with elongation (arrow). (ii) A tubule with continuous lumen (cross) developed. (iii) Structures with several elongations (arrow) was observed. (**E)** **Conditioned medium from *Foxd2* mutant cells** resulted in reduced tubulogenesis and buddings/elongation compared to wildtype conditioned medium. (i) Overview image. (ii) Closeup of a cyst with lumen (cross) typically seen in ureteric bud culture plus mutant conditioned medium. (**F)** **Statistical analysis of structure types observed.** The positive control GDNF/FGF1 showed significantly increased tubule-formation, as well as elongations/buddings and protuberances compared to negative control and both mutant conditioned media. Negative control (Normal medium) as well as both mutant conditioned media resulted in formation of cysts/spheroids without signs of budding or elongation while wildtype conditioned medium showed a significant increase in tubulogenesis, elongations/buddings and protuberance formations compared to both mutant conditioned media and negative control. n = 3 independent experiments. Per experiment 94-102 structures were counted. For p-value calculation unpaired Student´s t-test was performed. **p* < 0.05. **(G)** **Graphical description and definition of structures seen in ureteric bud 3D culture.** **(H)** **Diameter of structures of each condition was analyzed.** GDNF/FGF1 treatment resulted diameters of ~50 µm reflecting a collecting tubules diameter. Non conditioned and mutant conditioned medium resulted in diameters of ~25 µm while wildtype conditioned medium resulted in diameters of ~35 µm; n = 2 independent experiments, 94-101 structures per experiment were analyzed. Unpaired student´s t-test was performed to analyze p-value. P-value **p* < 0.05, ****p* < 0.001, *****p* < 0.0001, ns=not significant.

***Supplementary Table 1***

*Please see attached Excel file Supplementary Table 1 sheet 1 and 2.*

**Supplementary Table 1, sheet 1.** **List of homozygous variants at MAF 1.0% detected with exome sequencing in the index individual of family 1**. Variants listed as “benign” or likely “benign” in ClinVar (https://www.ncbi.nlm.nih.gov/clinvar/) have been deleted for the sake of clarity. Only intronic variants +/- 20 bp to exon-intron boundary are shown.

**Supplementary Table 1, sheet 2.** **List of rare, homozygous variants detected with exome sequencing in the index individual of family 3**. Filters applied included homozygosity in the proband but not the mother, absence from the gnomAD database, high or moderate quality as determined by the automated pipeline, and variant type that included missense, splice or intronic variants.

***Supplementary Table 2***

| **Location (hg19)** | chr1:g.47904435 | chr2:g.31178838 | chr2:g.32824825 |
| --- | --- | --- | --- |
| **Gene** | *FOXD2* | *GALNT14* | *BIRC6* |
| **RefSeq** | NM_004474.4 | NM_001253826.1 | NM_016252.3 |
| **Strand** | Forward | Reverse | Forward |
| **Phenotype MIM Number** | - | - | - |
| **Reference** | A | C | C |
| **Reference allele coverage of proband** | 0 | 0 | 0 |
| **Genotype of proband** | G/G | T/T | T/T |
| **Alternative allele coverage of proband** | 246 | 204 | 105 |
| **Other relatives tested with Sanger sequencing** | Father: A/G  Mother: A/G  Affected older sibling: G/G  Unaffected younger sibling: A/A | - | - |
| **Variant type** | Missense | Missense | Missense |
| **dbSNP ID** | - | rs145021697 | rs777498615 |
| **ClinVar ID** | - | - | - |
| **gnomAD allele frequency** | 0 | 0.0007883 | 0.0001310 |
| **Variant: Coding (HGVS nomenclature_c.)** | c.628A>G | c.487G>A | c.13850C>T |
| **Variant: Protein (HGVS nomenclature_p.)** | p.(Met210Val) | p.(Asp163Asn) | p.(Ala4617Val) |
| **SIFT Result^a^** | Damaging  (score: 0.03) | Damaging  (score: 0.01) | Damaging  (score: 0.01) |
| **PROVEAN Result^b^** | Deleterious  (score: 0.000) | Deleterious  (score: 0.085) | Deleterious  (score: 0.003) |
| **PolyPhen2 Result (HumVar)^c^** | Probably Damaging  (score: 0.997) | Probably Damaging  (score: 0.977) | Benign  (score: 0.301) |
| **MutationTaster Result** | Disease Causing  (score: 0.999) | Disease Causing  (score: 0.999) | Disease Causing  (score: 0.999) |
| **CADD Score^d^** | 25.9 | 32 | 24.4 |
| **Revel Score^e^** | 0.913 | 0.458 | 0.251 |
| **ClinPred Score^f^** | 0.992 | 0.048 | 0.352 |
| **MutPred2 Score ^g^** | 0.67 | 0.47 | 0.24 |

**Supplementary Table 2.** **List of filtered candidate variants detected with exome sequencing in index individual (II-1) of family 2**.

^a^ SIFT score of ≤0.05 indicates a damaging (deleterious) variant and the range 0.05 to 1.0 is predicted to be tolerated (benign). Variants with scores closer to 0.0 are more confidently predicted to be deleterious.

^b^PROVEAN categorizes variants as deleterious or neutral; a variant with a PROVEAN score of <-2.5 is deleterious, whereas variants with scores >-2.5 are considered neutral.

^c^Variants with a PolyPhen2 score >0.5 are predicted as ‘probably’ or ‘possibly’ damaging (Ranges: 0.0 - 1.0).

^d^Variants with a CADD score >20 are predicted to be more damaging than 99% of theoretical genome-wide substitutions.

^e^Variants with a REVEL score >0.45 are predicted as ‘deleterious’ and scored below this threshold are predicted as ‘benign’ (Ranges: 0.0 - 1.0).

^f^The decision boundary to classify the variant as pathogenic or benign is 0.5 for ClinPred (lower for benign, higher for pathogenic variants).

^g^The MutPred2 score is greater than 0.5 for a pathogenic.

***Supplementary Table 3***

| **Location (hg19)** | chr6:g.51914955 | chr2:g.216245576 | chrX:g.106066483 | chrX:g.106083996 |
| --- | --- | --- | --- | --- |
| **Gene** | *PKHD1* | *FN1* | *TBC1D8B* | *TBC1D8B* |
| **RefSeq** | NM_138694.4 | NM_212482.3 | NM_017752.3 | NM_017752.3 |
| **Strand** | Reverse | Reverse | Forward | Forward |
| **Phenotype MIM Number** | 263200 | 601894  184255 | 301028 | 301028 |
| **Reference** | C | C | T | G |
| **Reference allele coverage of proband** | 65 | 45 | 32 | 47 |
| **Genotype of proband** | C/T | C/T | T/C | G/A |
| **Alternative allele coverage of proband** | 44 | 32 | 45 | 50 |
| **Other relatives tested with next-generation sequencing** | Mother: C/T | Mother: C/T | Mother: T/C | Mother: G/A |
| **Variant type** | Missense | Missense | Missense | Missense |
| **dbSNP ID** | rs745770404 | rs775247659 | - | rs199795712 |
| **ClinVar ID** | VCV000188876.16 | - | - | - |
| **gnomAD allele frequency** | 4/251,138 | 3/251,102 | 0 | 1/183,133 |
| **Variant: Coding (HGVS nomenclature_c.)** | c.2279G>A | c.5392G>A | c.614T>C | c.1601G>A |
| **Variant: Protein (HGVS nomenclature_p.)** | p.Arg760His | p.Asp1798Asn | p.Val205Ala | p.Arg534His |
| **SIFT Result^a^** | Benign (Moderate)  (score: 0.431) | Benign (Supporting)  (score: 0.209) | Uncertain  (score: 0.001) | Deleterious (Supporting)  (score: 0) |
| **PROVEAN Result^b^** | Benign (Moderate)  (score: -1.33) | Benign (Moderate)  (score: -0.83) | Uncertain  (score: -3.33) | Pathogenic (Supporting)  (score: -4.34) |
| **PolyPhen2 Result (HumVar)^c^** | Uncertain  (score: 0.84) | Benign (Supporting)  (score: 0.05) | N/A | Deleterious (Moderate)  (score: 1) |
| **MutationTaster Result** | Deleterious  (score: 0.79) | Deleterious  (score: 1) | Deleterious  (score: 0.74) | Deleterious  (score: 1) |
| **CADD Score^d^** | 33 | 25 | 23.4 | 29.5 |
| **Revel Score^e^** | 0.5 | 0.18 | 0.55 | 0.53 |
| **gnomAD allele frequency** | 4/251,138 | 3/251,102 | 0 | 1/183,133 |
| **Variant classification*** | Pathogenic | VUS | VUS | VUS |

**Supplementary Table 3. Additional variants reported on next-generation sequencing in the index case in family 3.** VUS, variant of uncertain significance.

^a^ SIFT score of ≤0.05 indicates a damaging (deleterious) variant and the range 0.05 to 1.0 is predicted to be tolerated (benign). Variants with scores closer to 0.0 are more confidently predicted to be deleterious.

^b^PROVEAN categorizes variants as deleterious or neutral; a variant with a PROVEAN score of <-2.5 is deleterious, whereas variants with scores >-2.5 are considered neutral.

^c^Variants with a PolyPhen2 score >0.5 are predicted as ‘probably’ or ‘possibly’ damaging (Ranges: 0.0 - 1.0).

^d^Variants with a CADD score >20 are predicted to be more damaging than 99% of theoretical genome-wide substitutions.

^e^Variants with a REVEL score >0.45 are predicted as ‘deleterious’ and scored below this threshold are predicted as ‘benign’ (Ranges: 0.0 - 1.0).

* Variant classification – according to American College of Medical Genetics standards.

***Supplementary Table 4***

|  |  | Gibbs free energy between the wild‐type  and mutant proteins (ΔΔG; kcal/mol) | | | | |
| --- | --- | --- | --- | --- | --- | --- |
| Variant |  | | DynaMut2 | INPS-3D | FoldX5.0 | PremPS |
|  | 210VAL  (Family 2) | | -0.45 | -1.73 | 2.56 | 0.6 |
|  | 210ARG  (Family 3) | | 1.22 | -0.95 | 0.81 | 1.1 |

**Supplementary Table 4.** Online *in-silico* analysis tools used for evaluating protein stability changes upon the p.(Met210Val) and p.(Met210Arg) missense variants in FOXD2, and Gibbs free energy calculation results. INPS-3D and DynaMut2: ΔΔG<0 is destabilizing; ΔΔG>0 is stabilizing PremPS and FoldX: ΔΔG>0 is destabilizing; ΔΔG<0 is stabilizing.

***Supplementary Table 5***

*Please see attached Excel file Supplementary Table 5*

***Supplementary Table 6***

**A**

| **Sex** | **Genotype** | **Kidney Histology** | **Area kidney** **section in mm^2^** | **Number**  **of GM** | **Number of GM per mm^2^** |
| --- | --- | --- | --- | --- | --- |
| f | *Foxd2*:hom | Basophilic tubules | 16.3 | 87 | 5.34 |
| f | *Foxd2*:hom | NAD | 17 | 90 | 5.29 |
| m | *Foxd2*:hom | Bilateral renal pelvis dilatation | 16.7 | 90 | 5.39 |
| m | *Foxd2*:hom | Hyalin intratubular cast | 16.4 | 70 | 4.27 |
| m | *Foxd2*:hom | Basophilic tubules | 18.1 | 84 | 4.64 |
| m | *Foxd2*:hom | Bilateral renal pelvis dilatation Focal vacuolization | 20.2 | 89 | 4.41 |
| f | wt | NAD | 14.7 | 89 | 6.05 |
| f | wt | NAD | 13.3 | 85 | 6.39 |
| m | wt | NAD | 20.9 | 90 | 4.31 |
| m | wt | NAD | 15.8 | 83 | 5.25 |

**B**

**Supplementary Table 6.** (**A+B) Histological parameters of the examined kidneys.** GM, glomeruli; H&E, hematoxylin and eosin; hom, homozygous knockout; NAD, no alteration determined; wt, wild type.

***Supplementary Table 7***


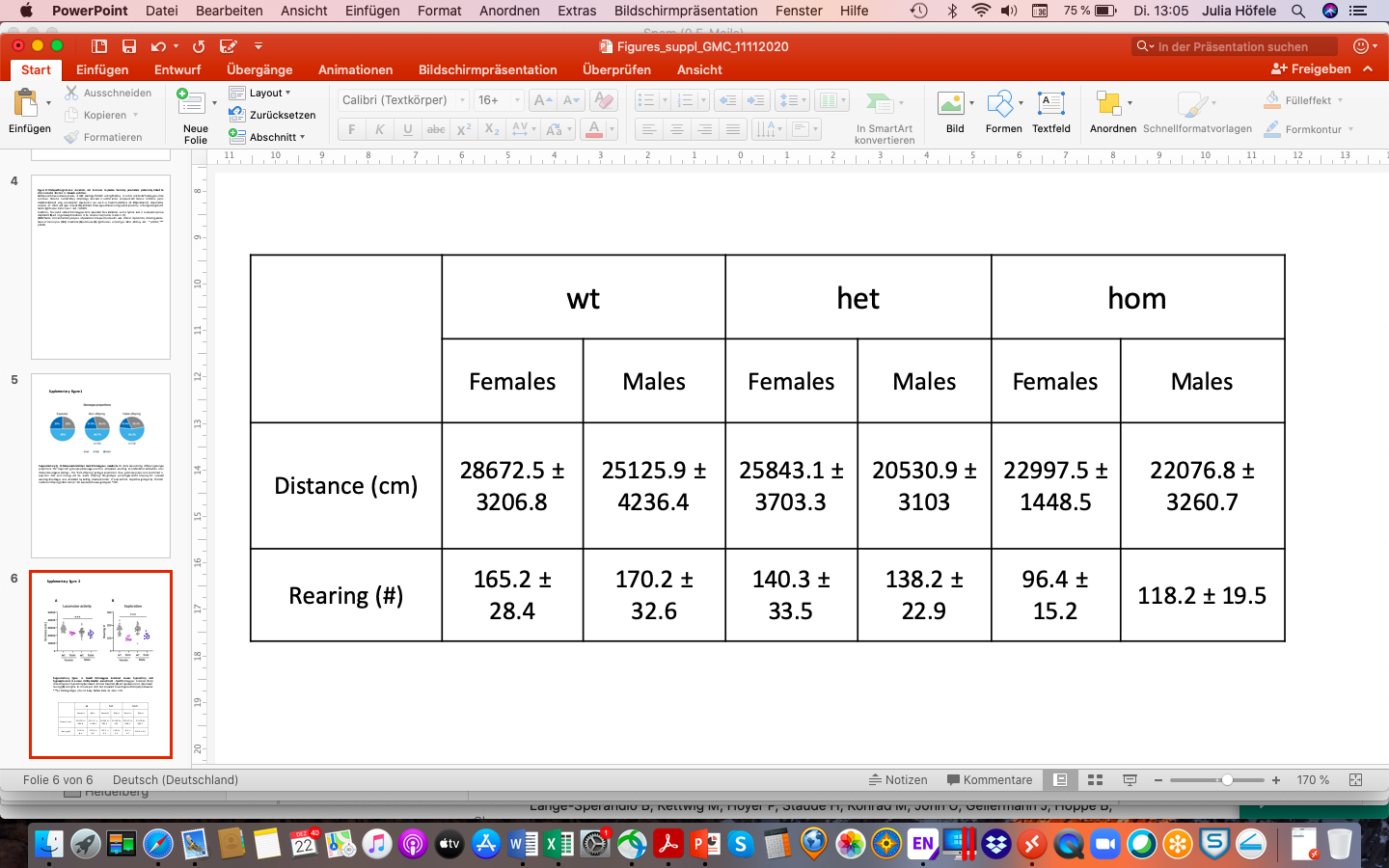


**Supplementary Table 7.** Total distance travelled and total rearing activity of wild type control mice, heterozygous and homozygous *Foxd2* knockout mice. wt, wild type; het, heterozygous; hom, homozygous.

**References**

1. Kremer LS, Bader DM, Mertes C, et al. Genetic diagnosis of Mendelian disorders via RNA sequencing. *Nat Commun*. Jun 12 2017;8:15824. doi:10.1038/ncomms15824

2. Griffin HR, Pyle A, Blakely EL, et al. Accurate mitochondrial DNA sequencing using off-target reads provides a single test to identify pathogenic point mutations. *Genet Med*. Dec 2014;16(12):962-71. doi:10.1038/gim.2014.66

3. Plagnol V, Curtis J, Epstein M, et al. A robust model for read count data in exome sequencing experiments and implications for copy number variant calling. *Bioinformatics*. Nov 1 2012;28(21):2747-54. doi:10.1093/bioinformatics/bts526

4. Robinson JT, Thorvaldsdottir H, Winckler W, et al. Integrative genomics viewer. *Nat Biotechnol*. Jan 2011;29(1):24-6. doi:10.1038/nbt.1754

5. Richards S, Aziz N, Bale S, et al. Standards and guidelines for the interpretation of sequence variants: a joint consensus recommendation of the American College of Medical Genetics and Genomics and the Association for Molecular Pathology. *Genet Med*. May 2015;17(5):405-24. doi:10.1038/gim.2015.30

6. Riggs ER, Andersen EF, Cherry AM, et al. Technical standards for the interpretation and reporting of constitutional copy-number variants: a joint consensus recommendation of the American College of Medical Genetics and Genomics (ACMG) and the Clinical Genome Resource (ClinGen). *Genet Med*. Feb 2020;22(2):245-257. doi:10.1038/s41436-019-0686-8

7. Abou Tayoun AN, Pesaran T, DiStefano MT, et al. Recommendations for interpreting the loss of function PVS1 ACMG/AMP variant criterion. *Hum Mutat*. Nov 2018;39(11):1517-1524. doi:10.1002/humu.23626

8. Kume T, Deng K, Hogan BL. Minimal phenotype of mice homozygous for a null mutation in the forkhead/winged helix gene, Mf2. *Mol Cell Biol*. Feb 2000;20(4):1419-25.

9. Bolger AM, Lohse M, Usadel B. Trimmomatic: a flexible trimmer for Illumina sequence data. *Bioinformatics*. Aug 1 2014;30(15):2114-20. doi:10.1093/bioinformatics/btu170

10. Li H, Durbin R. Fast and accurate long-read alignment with Burrows-Wheeler transform. *Bioinformatics*. Mar 1 2010;26(5):589-95. doi:10.1093/bioinformatics/btp698

11. Gormez Z, Bakir-Gungor B, Sagiroglu MS. HomSI: a homozygous stretch identifier from next-generation sequencing data. *Bioinformatics*. Feb 1 2014;30(3):445-7. doi:10.1093/bioinformatics/btt686

12. Einhorn Y, Einhorn M, Kurolap A, et al. Community data-driven approach to identify pathogenic founder variants for pan-ethnic carrier screening panels. *Hum Genomics*. Mar 28 2023;17(1):30. doi:10.1186/s40246-023-00472-w

13. Li H, Durbin R. Fast and accurate short read alignment with Burrows-Wheeler transform. *Bioinformatics*. Jul 15 2009;25(14):1754-60. doi:10.1093/bioinformatics/btp324

14. Garrison E, Marth G. Haplotype-based variant detection from short-read sequencing. *https://arxivorg/pdf/12073907pdf*. 2012;

15. Riedhammer KM, Nguyen TT, Kosukcu C, et al. Implication of FOXD2 dysfunction in syndromic congenital anomalies of the kidney and urinary tract (CAKUT). *medRxiv*. Mar 22 2023;doi:10.1101/2023.03.21.23287206

16. Schymkowitz J, Borg J, Stricher F, Nys R, Rousseau F, Serrano L. The FoldX web server: an online force field. *Nucleic Acids Res*. Jul 1 2005;33(Web Server issue):W382-8. doi:10.1093/nar/gki387

17. Rodrigues CHM, Pires DEV, Ascher DB. DynaMut2: Assessing changes in stability and flexibility upon single and multiple point missense mutations. *Protein Sci*. Jan 2021;30(1):60-69. doi:10.1002/pro.3942

18. Savojardo C, Fariselli P, Martelli PL, Casadio R. INPS-MD: a web server to predict stability of protein variants from sequence and structure. *Bioinformatics*. Aug 15 2016;32(16):2542-4. doi:10.1093/bioinformatics/btw192

19. Chen Y, Lu H, Zhang N, Zhu Z, Wang S, Li M. PremPS: Predicting the impact of missense mutations on protein stability. *PLoS Comput Biol*. Dec 2020;16(12):e1008543. doi:10.1371/journal.pcbi.1008543

20. Gailus-Durner V, Fuchs H, Becker L, et al. Introducing the German Mouse Clinic: open access platform for standardized phenotyping. *Nat Methods*. Jun 2005;2(6):403-4. doi:10.1038/nmeth0605-403

21. Fuchs H, Aguilar-Pimentel JA, Amarie OV, et al. Understanding gene functions and disease mechanisms: Phenotyping pipelines in the German Mouse Clinic. *Behav Brain Res*. Oct 15 2018;352:187-196. doi:10.1016/j.bbr.2017.09.048

22. Haeussler M, Schonig K, Eckert H, et al. Evaluation of off-target and on-target scoring algorithms and integration into the guide RNA selection tool CRISPOR. *Genome Biol*. Jul 5 2016;17(1):148. doi:10.1186/s13059-016-1012-2

23. Fuchs H, Gailus-Durner V, Adler T, et al. The German Mouse Clinic: a platform for systemic phenotype analysis of mouse models. *Curr Pharm Biotechnol*. Feb 2009;10(2):236-43. doi:10.2174/138920109787315051

24. Rathkolb B, Hans W, Prehn C, et al. Clinical Chemistry and Other Laboratory Tests on Mouse Plasma or Serum. *Curr Protoc Mouse Biol*. Jun 1 2013;3(2):69-100. doi:10.1002/9780470942390.mo130043

25. Fuchs H, Gailus-Durner V, Adler T, et al. Mouse phenotyping. *Methods*. Feb 2011;53(2):120-35. doi:10.1016/j.ymeth.2010.08.006

26. Puk O, de Angelis MH, Graw J. Longitudinal fundus and retinal studies with SD-OCT: a comparison of five mouse inbred strains. *Mamm Genome*. Jun 2013;24(5-6):198-205. doi:10.1007/s00335-013-9457-z

27. Holter SM, Garrett L, Einicke J, et al. Assessing Cognition in Mice. *Curr Protoc Mouse Biol*. Dec 2 2015;5(4):331-358. doi:10.1002/9780470942390.mo150068

28. Holter SM, Einicke J, Sperling B, et al. Tests for Anxiety-Related Behavior in Mice. *Curr Protoc Mouse Biol*. Dec 2 2015;5(4):291-309. doi:10.1002/9780470942390.mo150010

29. Bankhead P, Loughrey MB, Fernandez JA, et al. QuPath: Open source software for digital pathology image analysis. *Sci Rep*. Dec 4 2017;7(1):16878. doi:10.1038/s41598-017-17204-5

30. Oud MM, Latour BL, Bakey Z, et al. Cellular ciliary phenotyping indicates pathogenicity of novel variants in IFT140 and confirms a Mainzer-Saldino syndrome diagnosis. *Cilia*. 2018;7:1. doi:10.1186/s13630-018-0055-2

31. Mohammed SG, Arjona FJ, Verschuren EHJ, et al. Primary cilia-regulated transcriptome in the renal collecting duct. *FASEB J*. Jul 2018;32(7):3653-3668. doi:10.1096/fj.201701228R

32. Tosic J, Kim GJ, Pavlovic M, et al. Eomes and Brachyury control pluripotency exit and germ-layer segregation by changing the chromatin state. *Nat Cell Biol*. Dec 2019;21(12):1518-1531. doi:10.1038/s41556-019-0423-1

33. Loges NT, Antony D, Maver A, et al. Recessive DNAH9 Loss-of-Function Mutations Cause Laterality Defects and Subtle Respiratory Ciliary-Beating Defects. *Am J Hum Genet*. Dec 6 2018;103(6):995-1008. doi:10.1016/j.ajhg.2018.10.020

34. Schwartz GJ, Munoz A, Schneider MF, et al. New equations to estimate GFR in children with CKD. *J Am Soc Nephrol*. Mar 2009;20(3):629-37. doi:10.1681/ASN.2008030287
